# Novel N7-Arylmethyl Substituted Dinucleotide mRNA 5′ cap Analogs: Synthesis and Evaluation as Modulators of Translation †

**DOI:** 10.3390/pharmaceutics13111941

**Published:** 2021-11-16

**Authors:** Radoslaw Wojcik, Marek R. Baranowski, Lukasz Markiewicz, Dorota Kubacka, Marcelina Bednarczyk, Natalia Baran, Anna Wojtczak, Pawel J. Sikorski, Joanna Zuberek, Joanna Kowalska, Jacek Jemielity

**Affiliations:** 1 Centre of New Technologies, University of Warsaw, 02097 Warsaw, Poland; r.wojcik7@student.uw.edu.pl (R.W.); l.markiewicz@cent.uw.edu.pl (L.M.); m.bednarczyk@cent.uw.edu.pl (M.B.); n.baran@student.uw.edu.pl (N.B.); p.sikorski@cent.uw.edu.pl (P.J.S.); 2 Division of Biophysics, Institute of Experimental Physics, Faculty of Physics, University of Warsaw, 02093 Warsaw, Poland; marek.baranowski@fuw.edu.pl (M.R.B.); dorota.kubacka@fuw.edu.pl (D.K.); anna.wojtczak@fuw.edu.pl (A.W.); joanna.zuberek@fuw.edu.pl (J.Z.)

**Keywords:** transcription, translation, cap analogs, mRNA, eIF4E, DcpS

## Abstract

Dinucleotide analogs of the messenger RNA cap (m^7^GpppN) are useful research tools and have potential applications as translational inhibitors or reagents for modification of in vitro transcribed mRNAs. It has been previously reported that replacing the methyl group at the N7-position with benzyl (Bn) produces a dinucleotide cap with superior properties. Here, we followed up on this finding by synthesizing 17 novel Bn^7^GpppG analogs and determining their structure–activity relationship regarding translation and translational inhibition. The compounds were prepared in two steps, including selective N7-alkylation of guanosine 5′-monophosphate by arylmethyl bromide followed by coupling with imidazole-activated GDP, with total yields varying from 22% to 62%. The compounds were then evaluated by determining their affinity for eukaryotic translation initiation factor 4E (eIF4E), testing their susceptibility to decapping pyrophosphatase, DcpS—which is most likely the major cellular enzyme targeting this type of compound—and determining their translation inhibitory properties in vitro. We also synthesized mRNAs capped with the evaluated compounds and tested their translational properties in A549 cells. Our studies identified N7-(4-halogenbenzyl) substituents as promising modifications in the contexts of either mRNA translation or translational inhibition. Finally, to gain more insight into the consequences at the molecular level of N7-benzylation of the mRNA cap, we determined the crystal structures of three compounds with eIF4E.

## 1. Introduction

Eukaryotic mRNAs carry at their 5′ ends a protective cap. A typical cap consists of 7-methylguanosine linked to the first transcribed nucleotide in mRNA by a 5′,5′-triphopshate chain. The 7-methylguanosine cap can only be removed from the mRNA 5′ end by specialized decapping enzymes, and as such, protects mRNA from premature degradation by 5′ exonucleases. In addition, the cap fulfills numerous other biological functions that are mediated by specific cap-binding proteins and include maturation, transport, and eIF4E-mediated translation initiation [1,2,3]. Consequently, chemical modifications of the 5′ cap have the potential to modulate the properties of the whole mRNA, including its stability and translational efficiency. mRNAs modified within the 5′ cap can be obtained by incorporating chemically synthesized dinucleotide cap analogs into mRNA during in vitro transcription (so-called co-transcriptional capping) [4,5,6]. Modifications conferring superior biological properties to mRNAs are not only interesting objects for biological studies, but may also advance the use of mRNA as a therapeutic modality [7,8,9].

Dinucleotide cap analogs have also been investigated as small molecule inhibitors of cap-dependent translation, which could counteract elevated eIF4E levels in cancer cells [10,11,12,13,14]. Recently, we showed that conjugating dinucleotide cap analogs with transporting vehicles produces cell permeable compounds that induce apoptosis of cancer cells [15]. The potency of cap analogs as translation inhibitors can be further enhanced by increasing their affinity for eIF4E and conferring susceptibility to decapping pyrophosphatase, DcpS. 

Over the years, many dinucleotide cap analogs have been developed, both as reagents for mRNA 5′ end modification [16,17,18] and as small-molecule translational inhibitors [12,19,20]. One of the interesting examples is a dinucleotide cap analog carrying a benzyl group instead of methyl at the N7-position (Bn^7^GpppG) [11,21]. The modification slightly increased affinity for eIF4E, and when incorporated into mRNA, conferred almost twofold higher translation efficiency in vitro relative to m^7^GpppG-capped mRNAs, making it a good candidate for augmenting the properties of synthetic mRNAs. This effect was attributed to a combination of an increased affinity for eIF4E and a higher degree of correct orientation during incorporation of the cap into mRNA [11]. In another study, Bn^7^GpppG was shown to have decreased susceptibility to DcpS pyrophosphatase, which makes it also an interesting starting point for the development of small-molecule translational inhibitors [22]. 

**Figure 1:**
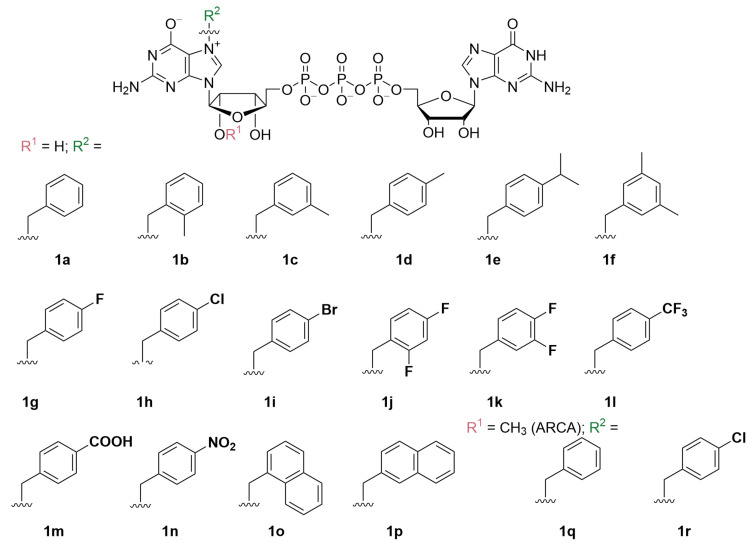
Structures of dinucleotide cap analogs synthesized and studied in this work. ARCA: anti-reverse cap analog.

Here, we sought to follow up on these findings and performed a detailed structure–activity relationship study for a series of derivatives of a N7-benzyl substituted cap analog **1a** (Figure 1). As a consequence, we report the synthesis and biological properties of seventeen novel N7-arylmethyl substituted dinucleotides, including two anti-reverse cap analogs (Figure 1) [23]. The compounds were evaluated using a broad range of biochemical, biophysical, and cellular assays both as reagents for modification of the RNA 5′ end and as small-molecule translation inhibitors, in order to identify several compounds with promising biological properties.

## 2. Results and Discussion

### 2.1. Synthesis of N7-Arylmethyl Cap Analogs

**Scheme 1:**
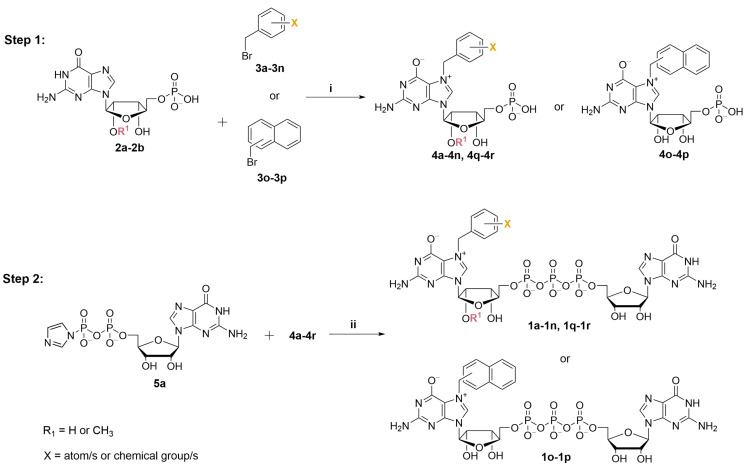
Synthetic approaches towards N7-arylmethyl cap analogs. Conditions: (i) DMSO, 45 °C, 24 h; (ii) DMSO, ZnCl_2_, rt, 24 h.

To obtain N7-substituted guanosine dinucleotides, we followed a two-step synthetic pathway (Scheme 1). First, an appropriate guanosine 5′-monophopshate (GMP) or 2′-*O*-methylguanosine 5′-monophosphate (2′-*O*-Me-GMP) was converted into an appropriate N7-arylmethyl derivative (**4a**–**4p**, Scheme 1, step 1), followed by coupling the product with imidazole-activated guanosine 5′-diphosphate (GDP-Im, **5a**) to produce the target cap analog (**1a**–**1p**; Scheme 1, Step 2).

Selective alkylation and arylmethylation of N7 position of GMP has been previously reported, and we decided to follow one of the already known protocols with minor modifications [24]. Differently substituted benzyl bromides were used as alkylating reagents (Figure S1). We hypothesized that substituents at the phenyl ring with different electronic and spatial properties might affect the ability of the benzyl group to interact with hydrophobic amino acids in the cap binding pockets of eIF4E. Therefore, we tested substituents differing in size, polarity, and electron withdrawing properties, e.g., nonpolar groups such as -methyl (**4b**–**4f**) and -isopropyl (**4e**); halogen atoms such as fluorine (**4g**, **4j**, **4k**), chlorine (**4h**, **4q**), and bromine (**4i**); nonpolar electron withdrawing groups such as -trifluoromethyl (**4l**) as well as highly polar or ionic groups such as -carboxyl (**4m**) and -nitro (**4n**). Moreover, using the same chemical approach, we synthesized two compounds with naphthalene moieties (**4o**, **4p**). To obtain N7-arylmethyl GMP derivatives in high yields we performed optimization studies, which revealed that the highest conversions (65–93% by HPLC) were achieved when the reactions were performed in DMSO at 45 °C for at least 24 h under vigorous stirring. Using the developed procedure followed by ion-exchange chromatography purification, the desired products were isolated at yields ranging from 14% to 82% (50% on average), wherein the highest yields were obtained for **4n** (82%) and **4g** (73%), and the lowest for **4b** (40%) and **4p** (14%), which may be caused by their relatively poor solubility in water and consequent higher losses during purification, as the observed HPLC conversions were substantially higher (Table S1). Besides standard cap analogs, we also synthesized two anti-reverse cap analogs (ARCAs) that contained 2′-*O*-Me groups following the same synthetic pathway (compounds **4q** and **4r**). Altogether, eighteen N7-arylmethylated guanosine monophosphate analogs were synthesized (**4a**–**4r**) with 40–82% yield (Table S1).

**Figure 2:**
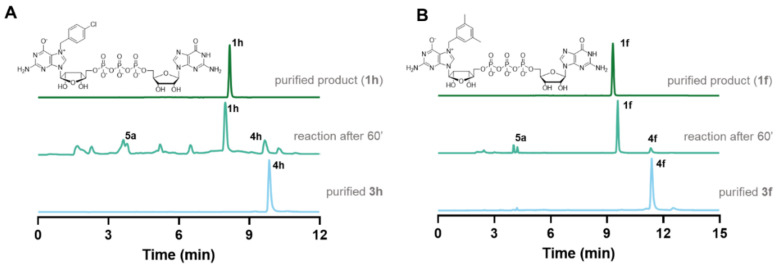
Representative RP HPLC profiles from the synthesis of **1h** and **1f.** (**A**) Synthesis of **1h**. Bottom: purified **4h** (4-Cl-Bn^7^GMP), middle: reaction mixture after 1 h, top: the final product (**1h**) after HPLC purification. (**B**) Synthesis of **1f**. Bottom: purified **4f** (3,5-di-MeBn^7^GMP), middle: reaction mixture after 1 h, top: the final product (**1f**) after HPLC purification.

Next, the synthesized N7-substituted GMP analogs were coupled with imidazole-activated 5′-guanosine 5′-diphosphate (**5a**; Scheme 1) [18]. The pyrophosphate bond formation reactions were performed in DMSO in the presence of excess ZnCl_2_ as a reaction mediator. The reaction progress was monitored using reverse-phase HPLC to reveal the rapid formation of the desired products, as exemplified by the reaction of **5a** and **4h** or **4f** which afforded the desired products **1h** or **1f**, respectively, within 1 h (Figure 2A,B). After purification by preparative HPLC compounds, **1h** and **1f** were isolated as ammonium salts in 22% and 62% yields, respectively (Figure 2A). An analogous straightforward procedure was applied to synthesize all dinucleotide analogs at 22–62% yields (Table 1). As a result, a series of eighteen N7-arylmethylated dinucleoside 5′,5′-triphopshates (**1a**–**1r**) was obtained. The compound structures and homogeneities were confirmed by HRMS, ^1^H NMR, ^31^P NMR, ^19^F NMR and RP HPLC (see Supplementary Materials).

### 2.2. Interaction with eIF4E and Susceptibility to Hydrolysis by DcpS

#### 2.2.1. Interaction with eIF4E

**Figure 3:**
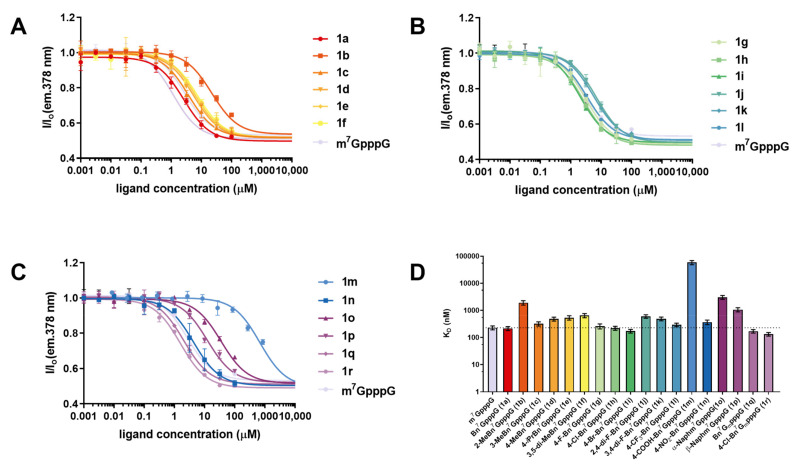
(**A**–**C**) Dose–response curves for N7-arylmethyl cap analogs and m^7^GpppG reference; (**D**) Comparison of the calculated *K*_D_ values. Data are mean values ± SD from triplicate experiments. The *K*_D_ values are summarized in Table 2. G_m_ denotes 2′-*O-*Me-Guanosine.

High affinity for eIF4E is a desirable feature of cap analogs designed for modulating translation, either as small molecules or as a part of mRNA. Thus, we studied how different substituents at the N7-position affected eIF4E-cap interaction. To this end, we first determined the affinity of m^7^GpppG and Bn^7^GpppG (**1a**) using time-synchronized fluorescence quenching titration, a very precise and well-established, but low-throughput method. Next, to evaluate the whole set of the synthesized compounds (**1a**–**1r**), we used a high-throughput competitive-binding assay based on a pyrene-labeled fluorescent probe [25]. From this assay we determined relative affinities of the compounds represented by half-effective concentrations (*EC*_50_) (Figure 3A–C), which were then used to calculate dissociation constant (*K*_D_) values for cap analog–eIF4E complexes using previously reported equations (Table 2, Figure 3D) [26].

Comparing the binding constant values *K*_D_ of compound **1a** with the native cap structure (m^7^GpppG), we found that replacing the methyl group at the N7 position of guanosine slightly stabilized the complex with eIF4E, which is in agreement with previous findings (Table 1) [11]. The *K*_D_ values determined from the high-throughput assay were in qualitative agreement with data from time-synchronized fluorescence quenching titration, justifying the use of the former for evaluation of the whole set of caps. Most of the studied modifications of the benzyl group did not affect the binding affinity significantly, which indicated that they were well accommodated in the cap binding pocket. Methyl groups were fairly well accepted when introduced at the meta and para positions of the benzyl moiety (**1c** and **1d**), but decreased the binding affinity by eight-fold when introduced at the ortho position (**1b**). Single halogen substitutions at the para position (**1g**, **1h**, **1i**) did not disrupt and even slightly stabilized the complex, whereas double substitutions (**1j**, **1k**) destabilized the complex, probably due to steric hindrance. Similar effect of steric hindrance was also observed for the isopropyl group introduced at the para position (**1e**), for which the *K*_D_ increased more than double in comparison to m^7^GpppG and **1a**. The presence of an electron withdrawing nitro group (**1n**) at the para position was accepted by the protein, whereas the negatively charged carboxyl group (**1m**) completely disrupted the binding. Replacing benzyl with naphthylmethyl (**1o** and **1p**) disrupted the binding to a similar extent as 2-methylbenzyl. Interestingly, for two ARCA analogs, a twofold increase in cap-binding affinity to eIF4E was observed compared to unmodified counterparts (**1q** with **1a** and **1r** with **1h**, respectively), despite previous studies that revealed that the addition of 2′-*O*-methylgroup to 7-methylguanosine did not affect the interaction with eIF4E. To additionally verify the binding affinity data (especially for **1q** and **1r**, as mentioned above) [11], we determined the binding affinities for all analogs using another independent assay, based on fluorescence anisotropy [27]. The *K*_D_ data determined by this method correlated very well with the results reported above (Table S2, Figure S2).

#### 2.2.2. Susceptibility to the Human DcpS Enzyme

**Figure 4:**
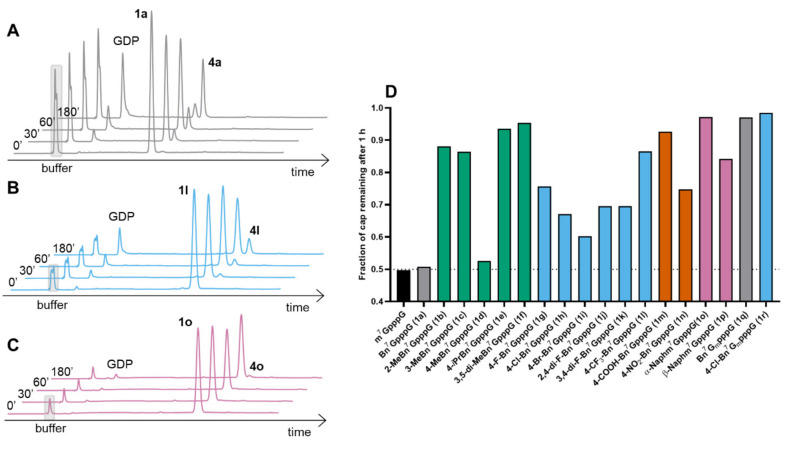
Susceptibility of cap analogs to the hDcpS enzyme studied via HPLC assay. Representative HPLC profiles from reaction mixtures containing Bn^7^GpppG, (**1a**, (**A**)), 4-CF_3_-Bn^7^GpppG, (**1l**, (**B**)) and α-Naphm^7^GpppG, (**1o**, (**C**)). (**D**) The fraction of cap analog remaining after 1 h. The numerical values are summarized in Table 2. G_m_: 2′-*O-*Me-Guanosine.

Susceptibility to decapping scavenger (DcpS) is one of the factors that could potentially influence the cellular stability of mono- and dinucleotide cap analogs designed as translational inhibitors. Therefore, we studied the susceptibility of arylmethyl-modified dinucleotides to DcpS. The compounds (at 100 μM concentration each) were incubated with 20 nM of hDcpS, and the hydrolysis progress at different time points was analyzed via RP HPLC and compared to m^7^GpppG (Figure 4 and Figure S3). Under these conditions the conversion of m^7^GpppG and Bn^7^GpppG (**1a**) was very similar (approximately 50% after 1 h of reaction; Figure 4A). In both cases the cleavage products were GDP and corresponding N7-substituted guanosine 5′-monophospate (m^7^GMP and Bn^7^GMP respectively), which suggested that the benzyl group did not protect against degradation from hDcpS, and the natural degradation pathway between β-γ position of triphosphate bridge was conserved, despite the previously reported data that suggested some protective effect of the N7-benzyl group [22]. Some substituents within the benzyl group resulted in decreased susceptibility of cap analogs to hDcpS. Analogs carrying CH_3_ at orto (**1b**) or meta (**1c**) positions of the phenyl ring were very slowly hydrolyzed, while analog **1d** with a methyl group at the para position was almost as good a substrate as Bn^7^GpppG (**1a**). Dinucleotides possessing fluoro (**1g**, **1j**, **1k**), chloro (**1h**), bromo (**1i**) and nitro (**1n**) substituents were also susceptible to hDcpS, and their conversion after 1 h of incubation with enzyme neared 70% (Figure 4D). Lower susceptibility to hydrolysis was observed for dinucleotides possessing bulkier substituents including isopropyl (**1e**), two methyl groups at the meta positions (**1f**), a trifluoromethyl group (**1l**, Figure 4B) or a negatively charged carboxyl group (**1m**). Two naphthalene-bearing analogs (**1o**, **1p**) (Figure 4C) were also characterized by lower susceptibility to hDcpS than m^7^GpppG. Interestingly, combination of benzyl (**1q**) or p-chlorobenzyl (**1r**) substituents with a 2′-*O*-methyl group at the ribose (i.e., ARCA modification) afforded compounds that were resistant to hDcpS under the experiment conditions (Table 2), which is probably a result of combining two modifications that slow down the rate of hydrolysis [20]. 

### 2.3. Inhibition of Translation by Cap Analogs in a RRL System

After determining the affinity for eIF4E and susceptibility to DcpS, we evaluated the analogs as inhibitors of protein translation in rabbit reticulocyte lysates (RRL). To enable better assessment of how these two factors influenced inhibitory properties, we performed two sets of experiments. In the first set, cap analogs were added to RRL along with luciferase-encoding reporter mRNA followed by a protein activity (luminescence) readout after 1 h, whereas in the second set cap analogs were additionally preincubated in RRL for 1 h prior to addition of mRNA. Based on previously reported findings [28], we expected that in the second experimental setup, cap analogs susceptible to DcpS would lose their inhibitory potency, whereas compounds resistant to DcpS would remain similarly active.

**Figure 5:**
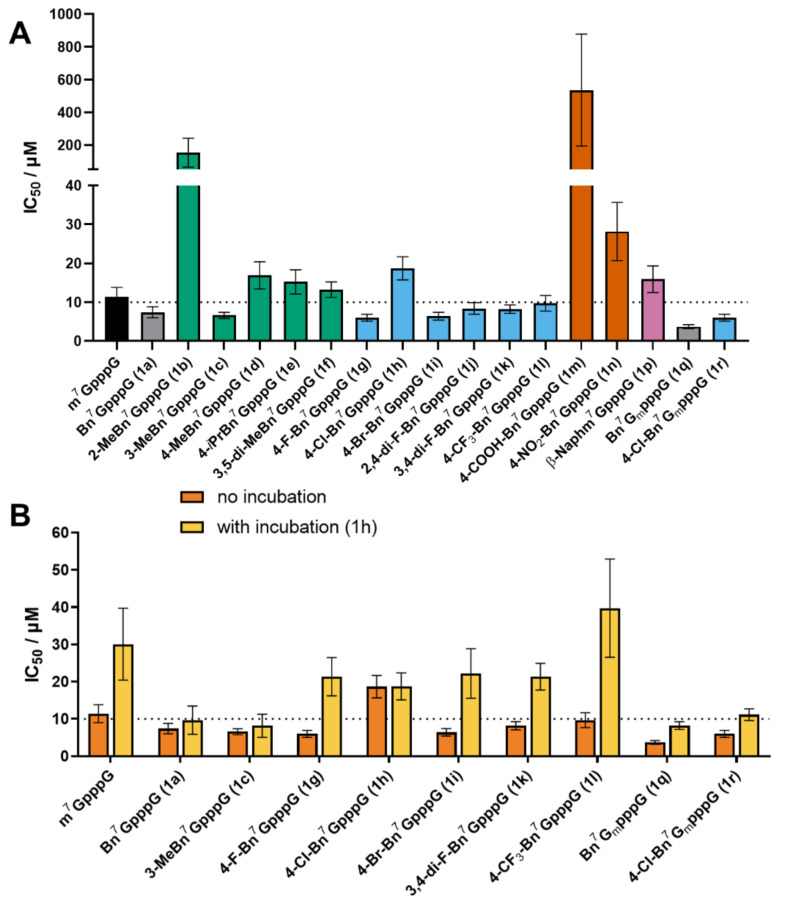
Translation inhibition by novel cap analogs in RRL. (**A**) IC_50_ values of all tested caps measured in conditions without cap pre-incubation; (**B**) Comparison of IC_50_ values obtained from two experimental variants (w/o cap pre-incubation and with 1 h incubation). The numerical values are summarized in Table 2. G_m_: 2′-*O-*Me-Guanosine.

In the first experimental setup, most of the analogs showed good translation inhibitory properties, with the potency qualitatively correlating with *K*_D_ values for eIF4E. Compounds **1g**, **1i**, **1c**, and **1a** were the most potent translation inhibitors (IC_50_ values 6.0 ± 0.9, 6.4 ± 1.0, 6.6 ± 0.8, 7.4 ± 1.4 μM, respectively) among unmethylated cap derivatives, and more potent than m^7^GpppG (IC_50_ 11.4 ± 2.4 μM). Compounds which showed weak or no affinity for eIF4E (**1b**, **1m**, **1n**) were also weak inhibitors or did not inhibit translation (Figure 5A). The addition of the methyl group at the 2′-*O*-position of 7-methylguanosine (**1q** and **1r**) additionally enhanced inhibitory properties, compared to unmethylated counterparts (**1a** and **1h**, respectively). The inhibitory properties of cap analogs incubated for 1 h decreased significantly in the case of m^7^GpppG, **1a**, **1g**, **1i**, **1k** and **1l**, which moderately correlates with the susceptibility to hDcpS in vitro (Figure 5B). However, for some of the hDcpS-susceptible compounds (e.g., compounds **1a** and **1h**), the inhibitory properties were retained after 1 h incubation despite the susceptibility to hDcpS. We speculate that in the case of these compounds, the degradation products (bn^7^GMP derivatives) may also exhibit notable inhibitory properties. The analogs that retained high translation inhibitory activity after incubation in RRL (compounds **1a**, **1c**, **1q**, **1r**) are good candidates for future development of cell-permeable cap analogs as translational inhibitors targeting eIF4E.

### 2.4. Incorporation into RNA and Translational Efficiency

**Figure 6:**
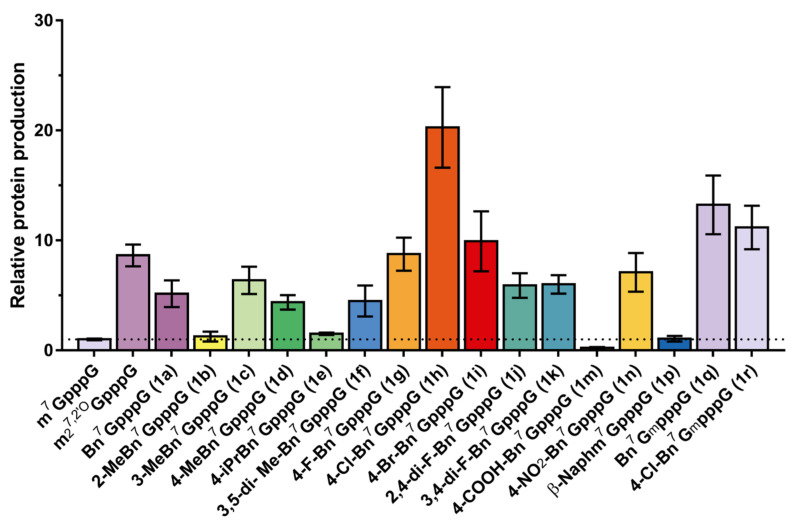
The influence of N7-benzylated dinucleotide 5′ cap analogues on the translational properties of HPLC-purified IVT mRNA. Total protein produced over 72 h by A549 cells transfected with HPLC-purified, capped mRNAs. Data shown are mean cumulative protein expression values from triplicate experiments (each consisting of three independent transfections; Figure S4). Bars are mean value normalized to m^7^GpppG RNA ± S.D.

Finally, we tested the properties of arylmethyl cap analogs as reagents for co-transcriptional mRNA capping. Previously, it was shown that Bn^7^GpppG has properties superior to m^7^GpppG in terms of both translational efficiency in RRL and mRNA capping [11]. Here, we sought to evaluate the translational properties of mRNAs capped with N7-arylmethyl analogs in cultured cells. To that end, the translation properties of transcripts capped with selected analogs were assessed in A549 cells. In vitro transcribed mRNAs encoding Gaussia luciferase, capped with compounds **1a**–**1k**, **1m**–**1n**, and **1p**–**1r** or with reference analogues m^7^GpppG and ARCA (m^7^G_m_pppG), were purified via RP HPLC, transfected into A549 cells, and the protein activity (luminescence) was measured using a reporter assay at 72 h post-transfection. The total amount of protein produced over 72 h from each capped RNA was normalized to m^7^GpppG RNA (Figure 6 and Figure S4). We found that the beneficial effect of N7-benzyl previously reported for an in vitro system (RRL) [11] was even more pronounced in living cells. Namely, replacing the N7-methyl group with a benzyl moiety (**1a**) increased protein production over fivefold in A549 cells compared to m^7^GpppG-capped mRNA (Figure 6). Interestingly, three of the studied modifications of the benzyl group additionally increased protein expression from capped mRNA. mRNA bearing cap analog **1h** (i.e., containing 4-chlorobenzyl moiety) increased protein production more than 22-fold when compared to m^7^GpppG-capped mRNA and 2.5-fold compared to ARCA. Surprisingly, the combination of the benzyl (**1q**) with a 2′-*O*-methyl group at the ribose (i.e., ARCA modification) provided an additional twofold increase compared to **1a**, whereas combination of ofp-chlorobenzyl (**1r**) with ARCA decreased the protein expression by twofold compared to **1h** (Figure 6, Table 2). This may suggest that the T7 RNA Pol-mediated incorporation into mRNA of N7-arylmethyl cap analogs with substituents bulkier than benzyl occurs only in the correct orientation, making the application of ARCA modification redundant or even disadvantageous in their case. 

**Table 2:** Summary of the biochemical properties of N7-benzyl-containing cap analogs and RNAs co-transcriptionally capped with these compounds.

Cap Analog	*K*_D_ Cap-eIF4E ± SD [nM] ^[a]^	hDcpS Assay ^[c]^	Translation Inhibition [μM]	Rel. Protein Expression ^[e]^
m^7^GpppG	229 ± 37 (160.0 ± 2.6) ^[b]^	0.49	11.4 ± 2.4 (30.1 ± 9.7) ^[d]^	1.0
m^7^G_m_pppG	n.d.	n.d.	n.d.	8.6 ± 1.0
Bn^7^GpppG (**1a**)	217 ± 36 (107.1 ± 4.7) ^[b]^	0.51	7.4 ± 1.4 (9.6 ± 3.8) ^[d]^	5.2 ± 1.2
2-MeBn^7^GpppG (**1b**)	1910 ± 360	0.88	154 ± 88	1.3 ± 0.4
3-MeBn^7^GpppG (**1c**)	319 ± 55	0.84	6.6 ± 0.8 (8.2 ± 3.1) ^[d]^	6.4 ± 1.2
4-MeBn^7^GpppG (**1d**)	482 ± 81	0.53	16.9 ± 3.5	4.4 ± 0.7
4-*i*PrBn^7^GpppG (**1e**)	534 ± 100	0.94	15.2 ± 3.1	1.5 ± 0.1
3,5-di-MeBn^7^GpppG (**1f**)	646 ± 114	0.95	13.2 ± 2.0	4.5 ± 1.4
4-F-Bn^7^GpppG (**1g**)	261 ± 54	0.76	6.0 ± 0.9 (21.4 ± 5.1) ^[d]^	8.7 ± 1.5
4-Cl-Bn^7^GpppG (**1h**)	221 ± 37	0.67	18.7 ± 3.0 (18.7 ± 3.6) ^[d]^	20.3 ± 3.7
4-Br-Bn^7^GpppG (**1i**)	172 ± 26	0.60	6.4 ± 1.0 (22.2 ± 6.7) ^[d]^	9.9 ± 2.7
2,4-di*-*F-Bn^7^GpppG (**1j**)	598 ± 87	0.70	8.4 ± 1.5	5.9 ± 1.1
3,4-di-F-Bn^7^GpppG (**1k**)	489 ± 77	0.70	8.2 ± 1.1 (21.4 ± 3.6) ^[d]^	6.0 ± 0.8
4-CF_3_-Bn^7^GpppG (**1l**)	293 ± 43	0.87	9.7 ± 2.0 (40 ± 13) ^[d]^	n.d.
4-COOH-Bn^7^GpppG (**1m**)	>50,000	0.93	>500	0.2 ± 0.1
4-NO_2_-Bn^7^GpppG (**1n**)	367 ± 71	0.75	28.2 ± 7.5	7.1 ± 1.8
α-Naphm^7^GpppG (**1o**)	3040 ± 490	0.97	n.d.	n.d.
β-Naphm^7^GpppG (**1p**)	1060 ± 190	0.84	15.9 ± 3.4	1.0 ± 0.3
Bn^7^G_m_pppG (**1q**) ^c^	171 ± 25	0.97	3.7 ± 0.5 (8.2 ± 1.0) ^[d]^	13.2 ± 2.7
4-Cl-Bn^7^G_m_pppG (**1r**)	132 ± 20	0.99	6.0 ± 0.9	11.2 ± 2.0

^[a]^ data from pyrene-based high-throughput competition assay. Measurements were carried out in 10 mM MOPS (pH 7.0), 100 mM KOAc, 2 mM MgCl_2_, 0.3 mM MnCl_2_, 2 mM DTT and 0.1% BSA at 30 °C. Fluorescence was excited at 345 nm and observed at 378 nm; ^[b]^ data from fluorescence quenching titration assay. Measurements were carried out in 50 mM Hepes (pH 7.2), 134.5 mM KCl, 0.5 mM EDTA, 1 mM DTT (I-150 mM) at 20 °C. Fluorescence was excited at 280 nm and observed at 340 nm; ^[c]^ Fraction of the cap remaining after 1 h incubation with DcpS; ^[d]^ Values in brackets are IC_50_ determined after incubating cap analogs in RRL for 1 h prior to adding mRNA; ^[e]^ Gaussia luciferase activity in A549 cells 72 h post-transfection. SD: standard deviation.

### 2.5. Crystallization and Structural Characterization of Murine eIF4E Complexes with Bn^7^GpppG, 3-MeBn^7^GpppG and 4-Cl-Bn^7^GpppG

**Figure 7:**
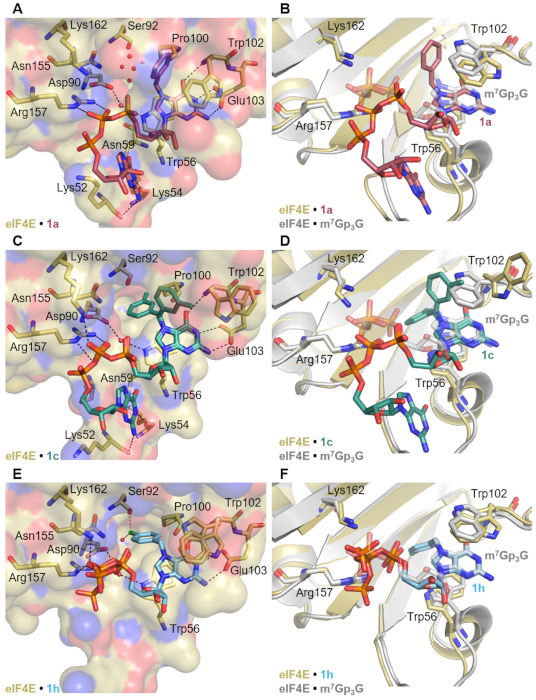
Crystal structure of (**A**) **1a**, (**C**) **1c**, and (**E**) **1h** in complex with murine eIF4E. Key residues involved in cap analog binding are shown as sticks. Hydrogen bonds are shown as black dotted lines, while polar contacts are marked in red dotted lines. The structural water molecules are indicated as red non-bonded spheres only in the cave formed by Asp90, Ser92, Asn155, Pro100, Trp 102 and Trp 56 for clarity. (**B**–**F**) Overlay of three solved structures of eIF4E (yellow carbon) in complex with **1a** (rose carbon), **1c** (teal carbon) and **1h** (cyan carbon), with the eIF4E (grey carbon) complex with m^7^GpppG (grey carbon) (PDB id: 1L8B, [29]), respectively. Relevant residues are shown in sticks, where dark blue is nitrogen, red oxygen, orange phosphate and light green chlorine.

Recognition of mRNA by eIF4E is important both for mRNA translation and for translational inhibition applications of the cap analogs. To understand the molecular basis of N7-arylmethyl cap analog recognition by eIF4E, we attempted to determine the crystal structures of the protein in complex with selected compounds. We obtained good quality crystals for Bn^7^GpppG (**1a**), 3-MeBn^7^GpppG (**1c**), and 4-Cl-Bn^7^GpppG (**1h**). Crystal structures were refined using diffraction data to the resolution of 2.2 Å for **1a**, 2.67 Å for **1c**, and 2.66 Å for **1h** (Table S3). The electron density map in the binding pocket was defined well enough to allow for the modeling of each cap analog (Figure S5). The overall protein fold for solved structures was virtually identical to that previously determined for m^7^GpppG (PDB ID: 1L8B; rmsd of 0.40 Å for complex with **1a**, 0.37 Å for complex with **1c** and 0.42 Å for complex with **1h**; all atoms were included). The conformation of almost all side chains for the residues forming the cap binding pocket (i.e., at ~3.5 Å distance around the cap analog) was also unchanged. The only pronounced differences were observed for the side chain of Trp102 present in the hydrophobic core of the cap-binding pocket (Figure 7A). In the **1a**–eIF4E complex the residue was flipped by 180° compared to the m^7^GpppG–eIF4E complex, but still participated in stacking interactions with modified guanine ring (the distance between the Bn^7^G plane and the parallelly aligned aromatic ring of Trp102 was approximately 3.5 Å) (Figure 7A,B). In contrast, in the complex with the 3-methylbenzyl dinucleotide (**1c**), Trp102 was pushed out from the binding pocket, disrupting the stacking interaction, which was most likely the cause of decreased binding affinity for this compound (Figure 7C,D). Finally, the electron density for the complex with the 4-chlorobenzyl dinucleotide (**1h**) indicated that the Trp102 could adapt two conformations—the one similar to that in the complex with m^7^GpppG, with the nitrogen atom directed towards the cap binding site, and the second flipped by 180° with the nitrogen atom directed towards the solution (similar to that observed for **1c**) (Figure 7E,F). The guanosine of 4-Cl-Bn^7^GpppG was not visible in the crystal structure, while the guanosines of Bn^7^GpppG and 3-MeBn^7^GpppG were stabilized by interactions with amino acids from the loop comprised of residues 50–59. Specifically, backbone carbonyl oxygen atoms of lysine 54 and 52 formed hydrogen bonds with N1 and N2 of guanine, respectively, and amide group (NH_2_) of Asn59 was a donor in a hydrogen bond with O6 of guanosine. In the reference m^7^GpppG-eIF4E structure, the ligand was oriented in the binding site such that the N7-methyl group was directed towards the cave filled with structural water molecules. The cave was partly polar (Asp90, Ser92 and Asn155) and partly hydrophobic (Val153, Phe94, Pro100, Leu60, Phe48, Trp166 and Trp56). Upon binding the cap, the eIF4E protein changed its conformation from open to closed, bringing the Trp102 and Trp56 closer to the cap, and together with the structural water molecules, stapling the ligand with the protein structure [29,30,31,32]. For the ligands carrying N7-benzylguanosine or its derivative, some of the interactions were lost or weakened. In the case of the benzyl modified cap **1a**, for which the benzyl ring was directed upwards, most of the structural water molecules were conserved in the cavity, while for 3-MeBn^7^GpppG and 4-Cl-Bn^7^GpppG the benzyl rings were directed downwards into the cavity, and the structural water molecules were replaced by 3-methylbenzyl rings or 4-chlorobenzyl substituents (Figure 7A,C,E). In the eIF4E complex with **1h** one structural water molecule was conserved, which together with the hydroxyl group of Ser92 appeared to form polar contacts with the chlorine atom. Overall, the comparison of these three crystal structures indicated that different benzyl substituents can be accommodated in the cap binding pocket due to (i) the conformational flexibility of Trp102, which was also previously observed for m^7^GMP analogs [33,34], and (ii) the presence of structural water molecules in the N7-methyl-binding cavity, which could be replaced upon introduction of larger hydrophobic substituents. The presence of a N7-benzyl substituent in the dinucleotide cap also influenced the conformation of the 5′,5′-triphosphate chain and its flexibility within the complex (Figure 7). For the eIF4E complex with 4-Cl-Bn^7^GpppG (**1h**), in which 4-chlorobenzyl appeared to be stabilized via polar contacts in the cavity, the phosphate chain could adopt two conformations, wherein one imitated the phosphate chain of m^7^GpppG (Figure 7F), while in the Bn^7^GpppG (**1a**) and 3-MeBn^7^GpppG (**1c**) complexes, γ and β phosphates were shifted out of the pocket by 2–3 Å (distances measured between corresponding phosphorus atoms, Figure 7B,D). Consequently, this weakened the interaction of the phosphate chain with the positively charged side chains of Arg157 and Lys162, mitigating the potential stabilization introduced by additional hydrophobic interactions of the benzyl group. The phosphate chain of compound **1a** was stabilized in complex with eIF4E only by interaction with the guanidyl group of Arg157 via two hydrogen bonds (hydrogen bond lengths 2.9–3.2 Å). The phosphate chains of **1c** and **1h** formed hydrogen bonds with side chains of Arg157 and Lys162 (hydrogen bond lengths of 3.1–3.5 Å); however, these interactions were weaker than that observed in the eIF4E complex with m^7^GpppG (PDB ID: 1L8B; hydrogen bond lengths 2.7–2.9 Å). Overall, the comparison of these three crystal structures indicated that even minor substitutions at the N7-benzyl moiety might affect both the Trp102 orientation and cap alignment in the cap-binding pocket, which highlights potential difficulties in studying this system through computational methods.

## 3. Conclusions

In this work we aimed to determine the structure–activity relationship for a series of N7-arylmethyl substituted cap analogs in the context of mRNA translation and translational inhibition. As a result, we identified several modifications of the benzyl group that were accepted by the cap-binding translation initiation factor (eIF4E) and differently affected susceptibility to decapping pyrophosphatase (DcpS). These modifications included bromine, fluorine and chlorine substituents as well as methyl groups (as *meta* and *para* substituents of benzene ring) and bulkier naphthalene moieties. We also evaluated if those analogs could act as small molecule inhibitors of translation using an in vitro (RRL) system, or promote cap-dependent translation when incorporated at the 5′ end of mRNA. Among analogs that showed high translation inhibitory activity in RLL and retained their properties after incubation in RRL were compounds **1a** (Bn^7^GpppG), **1c** (3-MeBn^7^GpppG), **1q** (Bn^7^G_m_pppG), and **1r** (4-Cl-Bn^7^G_m_pppG). These compounds are good candidates for the future development of cell-permeable cap analogs as translational inhibitors targeting eIF4E. 

In turn, compounds carrying 4-halogenobenzyl substituents at the N7 position, 4-chloro (**1h**) in particular, were identified as promising tools for 5′ end modification of in vitro transcribed mRNAs due to enhanced protein expression in A549 cells. Moreover, crystallographic structures showed that the higher potency of **1h** may be a result of additional polar interactions of the chlorine atoms with water molecules and serine92, and because of conserved conformation of the triphosphate bridge. The knowledge gained from crystallographic data may be used for further exploration of N7-benzyl derivatives to reveal additional compositions with properties beneficial for applications in mRNA-based therapeutics. 

## 4. Materials and Methods

### 4.1. Starting Materials, Chemical Reagents, Analytical Procedures

Solvents, chemical reagents, and starting materials, including nucleotides GMP (disodium salt) and 2′-*O*Me-GMP (disodium salt), were obtained from commercial sources. Commercially available GMP disodium and 2′-*O*Me-GMP disodium salt were converted into triethylammonium salt by passing an aqueous solution of GMP disodium salt through Dowex 50W-X8 cationite resin in the triethylammonium form. The collected eluate was concentrated under reduced pressure with repeated additions of ethanol, and the residue was dried in a vacuum over P_4_O_10_ to produce a white solid triethylammonium salt. GDP-Im (**5a**) was synthesized as described in the literature [18].

#### 4.1.1. Nucleotide Purification via Ion-Exchange Chromatography

The synthesized nucleotides were purified via ion-exchange chromatography on a DEAE Sephadex A-25 (HCO_3_^−^ form) column. The column was loaded with reaction mixture and washed thoroughly with water (until the eluate did not precipitate within a AgNO_3_ solution) to elute solvents and reagents that did not bind to the column. Then, nucleotides were eluted using 0−0.7 M and 0−1.0 M gradients of TEAB in deionized water for nucleoside mono- and triphosphates, respectively. Collected fractions were analyzed spectrophotometrically at 260 nm, and fractions containing the desired nucleotide were analyzed via RP HPLC and poured together. The yields were calculated on the basis of optical density milliunit measurements (mOD = absorbance of the solution × volume in mL) of isolated products and corresponding starting materials (nucleotides or nucleotide P-imidazolide derivatives). Optical unit measurements were performed in 0.1 M phosphate buffer pH 7.0 at 260 nm for all dinucleotides and in 0.1 M phosphate buffer pH 6.0 at 260 nm for mononucleotides. After evaporation under reduced pressure with repeated additions of 96% and 99.8% ethanol (to decompose TEAB and remove residual water, respectively) and acetonitrile, nucleotides were isolated as triethylammonium (TEA) salts. Mononucleotides were used as TEA salts in further synthesis and dinucleotides were converted into ammonium via purification on semipreparative RP HPLC (as described in Section 4.1.2). In the latter case, the products were isolated after repeated freeze-drying of the collected HPLC fractions.

#### 4.1.2. Analytical and Preparative Reversed-Phase (RP) HPLC

Analytical HPLC was performed using a Supelcosil LC-18-T HPLC column (4.6 × 250 mm, 5μm, flow rate 1.3 mL/min) with a linear gradient of 0−100% of buffer B (buffer A:methanol, 1:1) in 0.05 M ammonium acetate buffer pH 5.9 (buffer A) for 7.5 min. Semipreparative HPLC was performed on a Discovery RP Amide C-16 HPLC column (25 cm × 2.12 mm, 5 μm, flow rate 5.0 mL/min) or Dr. Maisch VisionHT C18 HL (25 cm × 2 cm, 10 μm, flow rate 5.0 mL/min) with linear gradients of acetonitrile in buffer A (from 0% to 50% of ACN for 60 min). In both cases UV detection at 254 nm was used. After HPLC purification, the desired product was freeze-dried three times to remove excess acetate and acetonitrile.

#### 4.1.3. Spectroscopic Analysis of the Synthesized Compounds

The structure and homogeneity of each final dinucleotide cap analog was confirmed via RP HPLC, high-resolution mass spectrometry using negative electrospray ionization (HR MS(−)ES), and ^1^H NMR, ^31^P NMR, and ^19^F NMR spectroscopy. Intermediate compounds were characterized using RP HPLC and low resolution MS(−)ES. High-resolution mass spectra were recorded on an LTQ Orbitrap. NMR spectra were recorded on a Bruker Avance IIIHD 500 MHz spectrometer equipped with a high stability temperature unit using a 5 mm PABBO BB/ 19F-1H/ D Z-GRD probe, at 500.24 MHz (^1^H NMR), 470.67 MHz (^19^F NMR), and 202.49 MHz (^31^P NMR), and at 25 °C if not stated otherwise. The ^1^H NMR chemical shifts were referenced to sodium 3-(trimethylsilyl)-2,2′,3,3′-tetradeuteropropionate (TSP) (*δ*_H_ = 0 ppm) as an internal standard. The ^31^P NMR chemical shifts were referenced to 20% phosphorus acid in D_2_O (*δ*_P_ = 0 ppm) as an external standard. The ^19^F NMR chemical shifts were referenced to 10 mM NaF in D_2_O (*δ*_F_ = −121.5 ppm) as an external standard. Typical parameters for proton spectra were: pulse width 9.7 μs, acquisition time 3.28 s, equilibration delay 1.0 s, sweep width 10k, 65k points, resolution 0.30 Hz, number of transients 128–512. Typical parameters for phosphorus spectra were: pulse width 20 μs, acquisition time 0.4 s, equilibration delay 2 s, sweep width 8k, 65k points, resolution 2.5 Hz, number of transients 512–1024. Typical parameters for fluorine spectra were: pulse width 20.5 μs, acquisition time 0.6 s, equilibration delay 1.0 s, sweep width 114k, 131k points, resolution 1.7 Hz, number of transients 128–256.

### 4.2. Dinucleotide Stock Solutions

All dinucleotides (2–5 mg) were dissolved in distilled water (100–200 μL) and their concentration was calculated based on absorption at 260 nm in 0.1 M phosphate buffer pH 6.0 (mononucleotides) or pH 7.0 (dinucleotides) and using the molar extinction coefficients of 11.4 mM^−1^ cm^−1^ for mononucleotides and 22.6 mM^−1^ cm^−1^ for dinucleotides. The UV–Vis absorption spectra were recorded on a Shimadzu UV1800 spectrometer in a quartz cuvette with 1.0 cm path length in a range of 220.0 nm to 400.0 nm, step 1.0 nm, at 25 °C. The stock solutions were stored at −20 °C.

### 4.3. General Procedure A (GP-A): Synthesis of N7-Substituted Guanosine 5′-Monophosphate Analogs (X-Bn-GMP)

Triethylammonium salt of guanosine 5′-monophosphate (1 eq) was suspended in DMSO (0.5 mL/15 mg of nucleotide), and appropriate benzyl bromide was added (8–10 eq). The mixture was shaken vigorously at 45 °C for 24h. The reaction progress was monitored using RP HPLC. Then, the reaction was diluted with water (approx. 10-fold excess) and extracted with diethyl ether (3 × 10 mL). The organic fraction was rejected and residuals of organic solvent from the water phase were evaporated using a rotary evaporator under reduced pressure. The solution in a total volume of 10–15 mL was applied onto DEAE-Sephadex resin as described earlier. The final product was isolated as triethylammonium salt and its structure was confirmed by low-resolution mass spectrometry (ESI).

### 4.4. General Procedure B (GP-B): Synthesis of Novel Cap Analogs (X-Bn-GpppG)

To a mixture of 5′-diphosphate guanosine P-imidazolide (GDP-Im/Na, 1 eq) were added X-Bn-GMP (1 eq) triethylammonium salt, DMSO (1 mL per 1.5 mg of P-imidazolide; higher amounts of DMSO were used in order to improve the solubility of benzyl GMP analogs) and ZnCl_2_ (8–12 eq) and the mixture was stirred for 24 h. The reaction progress was monitored using RP HPLC until substrates were converted to a product with satisfactory yield. Then, the reaction was quenched by addition of a mixture of Na_2_EDTA (8 eq) and NaHCO_3_ (half the mass of Na_2_EDTA) dissolved in deionized water. The product was purified via DEAE-Sephadex chromatography followed by additional purification using RP HPLC as described in Section 4.1.1 and Section 4.1.2.

### 4.5. Crystallization

The murine eukaryotic translation initiation factor 4E (eIF4E, residues 28–217) was expressed and purified as described previously [35]. After the final purification step on a gel filtration column, eIF4E was stored in a buffer containing 20 mM HEPES pH 7.2, 100 mM KCl, 0.5 mM EDTA, and 2 mM DTT. For crystallization, aliquots of the protein concentrated to 4.75 mg/mL (Amicon 10 kDa cut-off Ultra Centrifugal Filter Device) were incubated with 1 mM of the cap analogs **1a**, **1c** and **1h** at rt for approximately 15 min. Crystallization trials were performed at 18 °C using the sitting-drop vapor diffusion method. Initial crystallization hits were identified in PEGRx Screen (Hampton Research). After the optimization of crystal growth conditions using the hanging-drop vapor diffusion method, the best diffracting crystals were obtained in: 0.1 M Bis-Tris propane pH 9.0, 0.1 M NaCl, 25% *w*/*v* PEG 1500 for complex eIF4E–**1a** (Bn^7^GpppG); 0.1 M sodium citrate tribasic pH 5.5, 20% PEG 3350 for complex eIF4E–**1c** (3-MeBn^7^GpppG); 0.1 M Bicine pH 8.5, 0.2 M sodium formate, 19% PEGMME 5000 for complex eIF4E–**1h** (4-Cl-Bn^7^GpppG). Crystals were cryoprotected in 30% glycerol and flash frozen in liquid nitrogen.

### 4.6. Structure Determination and Refinement

X-ray diffraction data sets were collected at 100K with Bessy II (Helmholtz-Zentrum Berlin, Germany) Beamline 14.1 using a Dectris PILATUS 6M detector and processed using XDS with XDSAPP GUI [36]. Structures were solved to 2.2 Å for eIF4E–**1a**, to 2.67 Å for eIF4E–**1c** and 2.66 Å for eIF4E–**1h**. Phases were determined using molecular replacement in the Phaser-MR module of Phenix [37] and the structure of the eIF4E–m7GpppG complex (PDB id 1L8B, chain B) as the search model [29]. Ligand descriptions were generated using the ProDrg tool [38]. The models were improved by multiple rounds of manual building in Coot [39] followed by refinement using Phenix.refine [40]. Structural validation was performed using MolProbity [41]. Data collection and refinement statistics are summarized in Table S3.

### 4.7. Susceptibility to DcpS Hydrolysis

The enzymatic activity of DcpS was assayed at 37 °C in 50 mM Tris∙HCl, pH 7.6 containing 200 mM KCl, 0.5 mM EDTA. DcpS cleavage assays were carried out with 100 μM of the tested compound and 20 nM (monomer) of the hDcpS. Enzymatic reaction progress was examined after 30, 60 and 180 min. As a control, a sample was examined before adding enzyme. Aliquots of 15 μL were terminated by heat inactivation of the enzyme for 3 min at 98 °C. The samples were analyzed via RP HPLC equipped with a Supelcosil LC-18-T column (4.6 × 250 mm, 5 μm, flow rate 1.3 mL/min).

### 4.8. Inhibition of Translation by Cap Analogues in a RRL System

The ability of the N7-benzyl cap analogues to inhibit cap-dependent translation was assayed in a rabbit reticulocyte lysate (RRL) system. Two sets of experiments were performed. In both variants, in vitro translation reactions (10 μL total volume) contained a tested cap analog (inhibitor) and an in vitro transcribed, ARCA-capped mRNA encoding *Gaussia* luciferase at 1 μg/mL final concentration. In experimental variant A, cap analogues at concentrations in the range of 0 to 64 μM and mRNA were added to the reaction mixture at the same time. Reactions were incubated at 30 °C for 60 min and then stopped by freezing in liquid nitrogen. In experimental variant B, the cap analogues were added at concentrations in the range of 0 to 160 μM. Reactions were pre-incubated at 30 °C for 60 min prior to addition of mRNA; after addition of mRNA incubation was continuous at 30 °C for 60 min and then stopped by freezing in liquid nitrogen. To detect luminescence from *Gaussia* luciferase, 50 μL of 10 ng/mL h-coelenterazine (NanoLight, Norman, OK, USA) in PBS was added to 10 μL of RRL and the luminescence was measured with a Synergy H1 (BioTek, Winooski, VT, USA) microplate reader.

### 4.9. Determination of the Dissociation Constants When Studying Cap Analog–eIF4E Interactions

To determine the dissociation constants of the library of N7-benzylated dinucleotide 5′ cap analogs for translation initiation factor 4E, a fluorescent competitive-binding assay (PyFLINT-B) was applied as previously described [25]. The experiments were performed on a Synergy™ H1 plate reader in 96-well, black, non-binding assay plates. Each well contained an appropriate buffer (50 mM Hepes/KOH pH 7.2, 100 mM KCl and 0.5 mM EDTA), pyrene probe m^7^Gp_5_C_4_Py (10 nM), substrate (in half-logarithmic dilutions from 850 μM to 0.03 μM for compound **1m** and from 100 μM to 0.003 μM for m^7^GpppG and the rest of the compounds) and protein eIF4E (75 nM). The m^7^Gp_5_C_4_Py was synthesized as describer earlier [25]. The pyrene probe was dissolved in water and concentration was determined spectrometrically at 345 nm using the extinction coefficient (21,000 M^−1^ cm^−1^). The eIF4E protein was centrifuged after defrosting (5 min, 4 °C, 10k rcf) and its concentration was determined spectrometrically at 280 nm using the extinction coefficient (52,940 M^−1^ cm^−1^). The reaction mixtures were preincubated (15 min, 300 rpm, 30 °C). All measurements were performed at 30 °C and the fluorescence detection at 345 nm excitation and 378 nm emission was recorded.

To determine *EC*_50_ values, GraphPad Prism 9.1.2 software was used. To fit the curves to the obtained data we used the following formula:

(1) $$Y=Bottom+\;\frac{Top-Bottom}{1+\;\frac{X}{EC_{50}}}$$

where: *X*, cap analog (ligand) concentration; *EC_50_*, concentration of the cap analog resulting in half-maximal response from the fluorescent probe–protein complex; *Bottom*, minimal signal from the negative control, which contains only the free probe; *Top*, maximal signal from the positive control, the mixture of the protein and the fluorescent probe.

The dissociation constants *K*_D_ were then calculated using the equation described by Nikolovska-Coleska et al. [26]:

(2) $$K_{D}=\frac{EC_{50}-\left[P_{t}\right]+{\left[PL\right]}_{50}\left(\frac{K_{D\_probe}}{{\left[L\right]}_{50}}+1\right)}{\frac{{\left[L\right]}_{50}}{K_{D}\_probe}+\frac{{\left[P\right]}_{0}}{K_{D\_probe}}+1}$$

where: *EC*_50_, concentration of the cap analog resulting in half-maximal response from the fluorescent probe–protein complex; [*P_t_*]*,* total protein concentration; [*PL*]_50_, concentration of the probe–protein complex at 50% inhibition; *K*_D_probe_, dissociation constant of the complex between the fluorescent probe and the protein; [*L*]_50_, probe concentration at 50% inhibition; [*P*]_0_, free protein concentration at 0% inhibition.

Fractions of the compounds were independently tested using a different method based on fluorescence anisotropy (FA) [27]. These experiments were performed on a Synergy™ H1 plate reader in 96-well, black, non-binding assay plates. Each well contained appropriate buffer (50 mM HEPES/KOH pH 7.2, 100 mM KCl, 0.5 mM EDTA i 1 mM DTT), the fluorescent probe m^7^GTP-triazol-FAM (10 nM), substrate (in a 2.08-fold dilution, starting from 60 μM) and protein eIF4E (100 nM). The m^7^GTP-triazol-FAM was synthesized as describer earlier [27]. All measurements were performed at 25 °C. Determined *EC*_50_ values were means ± SD of three independent experiments. The dissociation constants *K*_D_ were then calculated using the equation described by Nikolovska-Coleska as described earlier. These values are also summarized in Table S2.

### 4.10. Determination of Translational Efficiency

The influence of N7-arylmethylated dinucleotide 5′ cap analogues on the translational properties of HPLC-purified IVT mRNA is shown in Figure S4, Measurements are total protein produced over 72 h by A549 cells transfected with capped mRNAs. Each plot in Figure S4 represents a single experiment consisting of three independent transfections of the cells at the same passage. Bars show mean value normalized to m^7^G_m_pppG-RNA ± SD. In the main manuscript, all nine data points from three different cell passages were combined to present cumulative protein expression.

## Figures and Tables

**Table 1:** Reaction yields for cap analogs **1a**–**1r**.

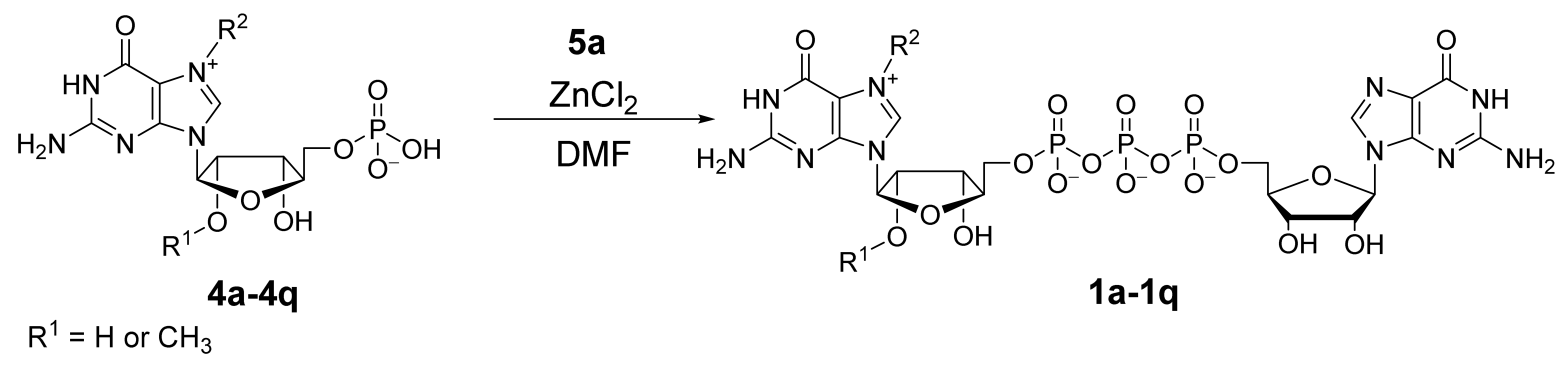				
Entry	Starting Material	Product	R^2^	Yield % ^a^ (%) ^b^
m^7^GpppG analogs (R^1^ = H)				
1	**4a**	**1a**	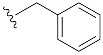	51 (83)
2	**4b**	**1b**	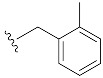	39 (82)
3	**4c**	**1c**	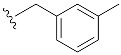	36 (58)
4	**4d**	**1d**	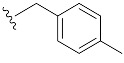	58 (76)
5	**4e**	**1e**	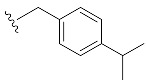	42 (74)
6	**4f**	**1f**	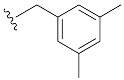	62 (84)
7	**4g**	**1g**	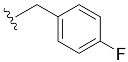	44 (82)
8	**4h**	**1h**	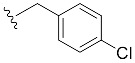	22 (75)
9	**4i**	**1i**	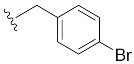	56 (88)
10	**4j**	**1j**	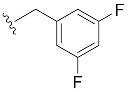	36 (80)
11	**4k**	**1k**	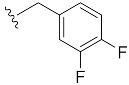	41 (66)
12	**4l**	**1l**	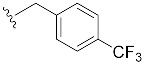	27 (70)
13	**4m**	**1m**	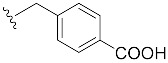	22 (81)
14	**4n**	**1n**	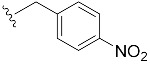	43 (90)
15	**4o**	**1o**	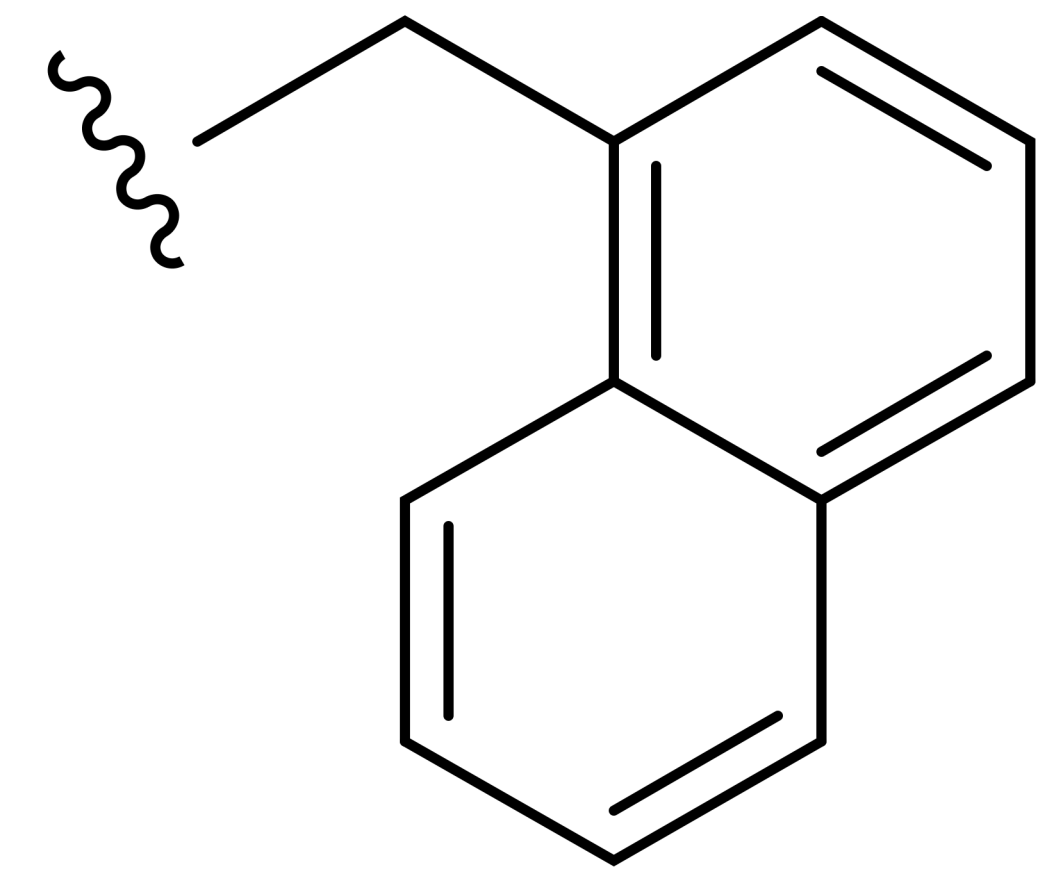	57 (89)
16	**4p**	**1p**	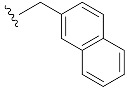	45 (81)
Anti-reverse cap analogs (ARCAs) (R^1^ = CH_3_)				
17	**4q**	**1q**	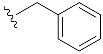	33 (49)
18	**4r**	**1r**	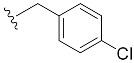	42 (86)

^a^ Isolated yield, ^b^ Conversion determined by HPLC.

## Data Availability

The atomic coordinates and structural factors have been deposited in the Protein Data Bank under accession codes 6YLR (eIF4E–Bn^7^GpppG (1a)), 6YLT (eIF4E–3-MeBn^7^GpppG (1c)), and 6YLV (eIF4E–4-Cl-Bn^7^GpppG (1h)).

## Supplementary Materials

The following are available online at www.mdpi.com/1999-4923/13/11/1941/s1, Chapter S1: Tables, Table S1: Structures and yields for N7-benzyl guanosine monophosphate analogues, Table S2: Comparison of EC50 and KD values determined by FLINT and Fluorescence Anisotropy methods, Table S3: Data collection and refinement statistics for crystal structures, Chapter S2: Figures, Figure S1: Structures of benzyl bromides used in this work, Figure S2: Correlation between the EC50 values determined from PyFLINT-B versus FA assays, Figure S3: Analysis of cap analog susceptibility to hydrolysis by DcpS using RP-HPLC, Figure S4: Relative total protein expression in three independents experiments. Data are normalized to m7GmpppG-RNA ± SD, Figure S5: Simulated annealing omit map (F0-Fc) contoured at 3σ around compound 1a (A), 1c (B), 1h (C) and Trp102 from the solved X-ray structures of eIF4E in complex with cap analog, Chapter S3: Chemical synthesis of novel Gp3G cap analogs, Chapter S4: Spectroscopic data: 1H, 31P, 19F NMR, HRMS and UV/VIS spectra.
