# Supplementary material for: Novel N7-Arylmethyl Substituted Dinucleotide mRNA 5′ cap Analogs: Synthesis and Evaluation as Modulators of Translation
Source: Pharmaceutics. 2021 Nov 16;13(11):1941. doi: 10.3390/pharmaceutics13111941 (PMC8623273; doi:10.3390/pharmaceutics13111941)
Supplement: Supplementary file 1 [file pharmaceutics-13-01941-s001.zip › pharmaceutics-1441065-supplementary.pdf]

pharmaceutics

Supplementary Materials: Novel N7-Arylmethyl Substituted Dinucleotide mRNA 5′cap Analogs: Synthesis and Evaluation as Modulators of Translation

Radoslaw Wojcik, Marek R. Baranowski, Lukasz Markiewicz, Dorota Kubacka, Marcelina Bednarczyk, Natalia Baran, Anna Wojtcza, Pawel J. Sikorski, Joanna Zuberek, Joanna Kowalska, and Jacek Jemielity

Supplementary Information

Table of contents

S1. Tables 2

S2. Figures 7

S3. Chemical synthesis of novel Gp_3G cap analogs 12

S4. Spectroscopic data: ^1H, ^{31}P, ^{19}F NMR, HRMS and UV/VIS spectra 21

S1. Tables

Table S1. Structures and yields for N7-benzyl guanosine monophosphate analogues.

**N7-benzylated guanosine monophosphate analogues**			
E.	Starting material	Product	Yield %[a] (%)[b]
1	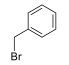 **3a**	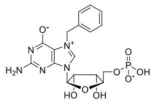 **4a**	66 (84)
2	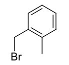 **3b**	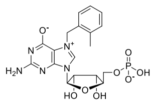 **4b**	40 (65)
3	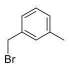 **3c**	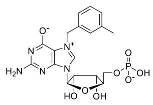 **4c**	49 (86)
4	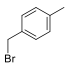 **3d**	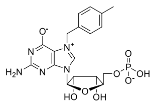 **4d**	64 (80)
5	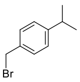 **3e**	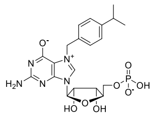 **4e**	58 (82)
6	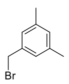 **3f**	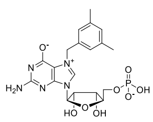 **4f**	47 (88)
7	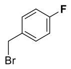 **3g**	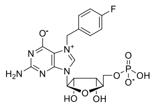 **4g**	73 (79)
8	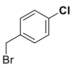 **3h**	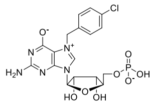 **4h**	45 (70)

9	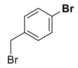 **3i**	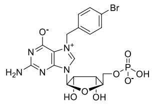 **4i**	42 (83)
10	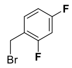 **3j**	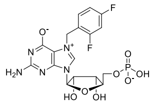 **4j**	52 (72)
11	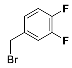 **3k**	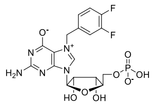 **4k**	52 (80)
12	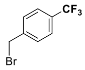 **3l**	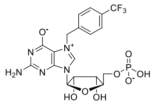 **4l**	44 (83)
13	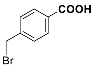 **3m**	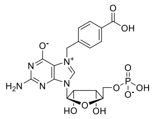 **4m**	63 (69)
14	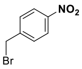 **3n**	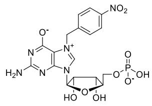 **4n**	82 (93)
15	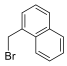 **3o**	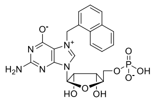 **4o**	71 (88)
16	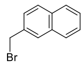 **3p**	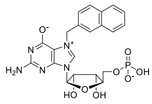 **4p**	14 (66)
**N7-benzylated 2′-*O*Me-guanosine monophosphate analogues**			
17	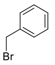 **3a**	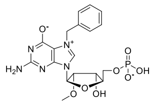 **4q**	51 (74)

18	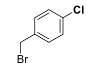 **3h**	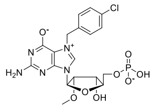 **4r**	48 (80)

[a]Isolated yield, [b]Conversion based on RP HPLC reaction profile

Table S2. Comparison of EC_{50} and K_D values determined by FLINT and Fluorescence Anisotropy methods.

			PyFLINT-B assay		FA assay	
**No.**	**Abbr.**	**Compound**	EC_{50} **(μM)**	K_D **(nM)**	EC_{50} **(μM)**	K_D **(nM)**
**1**		m^{7}GpppG	2.7 ± 0.3	229 ± 37	0.41 ± 0.01	127 ± 5
**2**	1a	Bn^{7}GpppG	$(2.2 \pm 0.3)\cdot 10^{1}$	217 ± 36	0.27 ± 0.02	74 ± 23
**3**	1b	2-MeBn^{7}GpppG	3.7 ± 0.4	$(191 \pm 36)\cdot 10^{1}$	3.1 ± 0.1	$(112 \pm 4.9)\cdot 10^{1}$
**4**	1c	3-MeBn^{7}GpppG	5.6 ± 0.6	319 ± 55	0.67 ± 0.02	222 ± 8
**5**	1d	4-MeBn^{7}GpppG	6.2 ± 0.8	482 ± 81	0.86 ± 0.03	295 ± 13
**6**	1e	4-*i*PrBn^{7}GpppG	7.5 ± 0.9	534 ± 100	1.3 ± 0.09	444 ± 33
**7**	1f	3,5-di-MeBn^{7}GpppG	3.1 ± 0.5	646 ± 114	1.1 ± 0.1	384 ± 41
**8**	1g	4-F-Bn^{7}GpppG	2.6 ± 0.3	261 ± 54	0.24 ± 0.08	64 ± 31
**9**	1h	4-Cl-Bn^{7}GpppG	2.0 ± 0.2	221 ± 37	0.34 ± 0.14	102 ± 53
**10**	1i	4-Br-Bn^{7}GpppG	6.9 ± 0.5	172 ± 26	0.21 ± 0.01	52 ± 3
**11**	1j	2,4-di-F-Bn^{7}GpppG	5.7 ± 0.5	598 ± 87	0.87 ± 0.05	298 ± 18
**12**	1k	3,4-di-F-Bn^{7}GpppG	3.4 ± 0.2	489 ± 77	0.88 ± 0.08	301 ± 30
**13**	1l	4-CF_3-Bn^{7}GpppG	$(6.7 \pm 0.8)\cdot 10^{2}$	293 ± 43	0.59 ± 0.04	194 ± 17
**14**	1m	4-COOH-Bn^{7}GpppG	4.3 ± 0.6	$(586 \pm 102)\cdot 10^{2}$	n.d	n.d
**15**	1n	4-NO_2-Bn^{7}GpppG	$(3.5 \pm 0.3)\cdot 10^{1}$	367 ± 71	0.52 ± 0.12	168 ± 43
**16**	1o	α-Naphm^{7}GpppG	$(1.2 \pm 0.1)\cdot 10^{1}$	$(304 \pm 49)\cdot 10^{1}$	2.1 ± 0.1	739 ± 49
**17**	1p	β-Naphm^{7}GpppG	2.0 ± 0.1	$(106 \pm 19)\cdot 10^{1}$	n.d	n.d
**18**	1q	Bn^{7}G$_m$pppG	1.6 ± 0.1	171 ± 25	0.18 ± 0.03	40 ± 11
**19**	1r	4-Cl-Bn^{7}G$_m$pppG	2.7 ± 0.3	132 ± 20	0.19 ± 0.02	45 ± 7

Table S3. Data collection and refinement statistics for crystal structures.

	**bn⁷GpppG (1a)**	**3Me-bn⁷GpppG (1c)**	**4-Cl-bn⁷GpppG (1h)**
**PDB code**	6YLR	6YLT	6YLV
**Wavelength (Å)**	0.9184	0.9184	0.9184
**Resolution range (Å)**	42.06 - 2.20 (2.27 - 2.20)	49.31 - 2.67 (2.77 - 2.67)	37.4 - 2.66 (2.76 - 2.66)
**Space group**	P 43	P 1	P 1
**Cell dimensions**	94.0 94.0 42.4 90 90 90	38.3 38.3 148.8 84.0 87.8 76.8	38.4 38.4 148.1 85.3 88.4 77.2
**Total reflections**	134095 (13102)	88377 (7780)	103757 (4113)
**Unique reflections**	19244 (1869)	23009 (2275)	22090 (1453)
**Multiplicity**	7.0 (7.0)	3.8 (3.4)	4.7 (2.8)
**Completeness (%)**	99.4 (96.9)	98.9 (96.8)	93.6 (60.4)
**Mean I/sigma(I)**	11.0 (2.0)	9.8 (2.0)	10.3 (2.0)
**Wilson B-factor (Å²)**	37.5	33.8	37.7
**R-merge**	0.147 (0.865)	0.134 (0.616)	0.112 (0.571)
**R-meas**	0.160 (0.934)	0.155 (0.730)	0.126 (0.680)
**$CC_{1/2}$**	0.995 (0.805)	0.99 (0.673)	0.991 (0.764)
**CC***	0.999 (0.944)	0.998 (0.897)	0.998 (0.931)
**Reflections used in refinement**	19182 (1824)	22996 (2274)	22080 (1453)
**Reflections used for R-free**	961 (92)	849 (84)	814 (54)
**R-work**	0.224 (0.357)	0.192 (0.240)	0.201 (0.253)
**R-free**	0.270 (0.359)	0.243 (0.316)	0.255 (0.325)
**CC(work)**	0.943 (0.724)	0.938 (0.823)	0.936 (0.837)
**CC(free)**	0.915 (0.682)	0.885 (0.851)	0.906 (0.759)
**Number of non-hydrogen atoms**	3046	6344	6292
**macromolecules**	2761	5685	5798
**ligands**	93	356	252
**solvent**	192	303	242
**Protein residues**	354	726	725
**RMS(bonds) (Å)**	0.003	0.008	0.006
**RMS(angles) (deg)**	0.76	0.97	0.76
**Ramachandran favored (%)**	98.0	98.5	98.0
**Ramachandran allowed (%)**	2.0	1.5	2.0
**Ramachandran outliers (%)**	0	0	0
**Rotamer outliers (%)**	0.0	0.2	0.2
**Clashscore**	3.5	6.4	7.2
**Average B-factor**	58.3	42.0	44.7
**macromolecules**	59.2	41.5	44.5
**ligands**	56.9	53.3	58.5
**solvent**	47.2	37.9	35.6

**Number of TLS groups**	12	25	25

Statistics for the highest-resolution shell are shown in parentheses.

S2. Figures

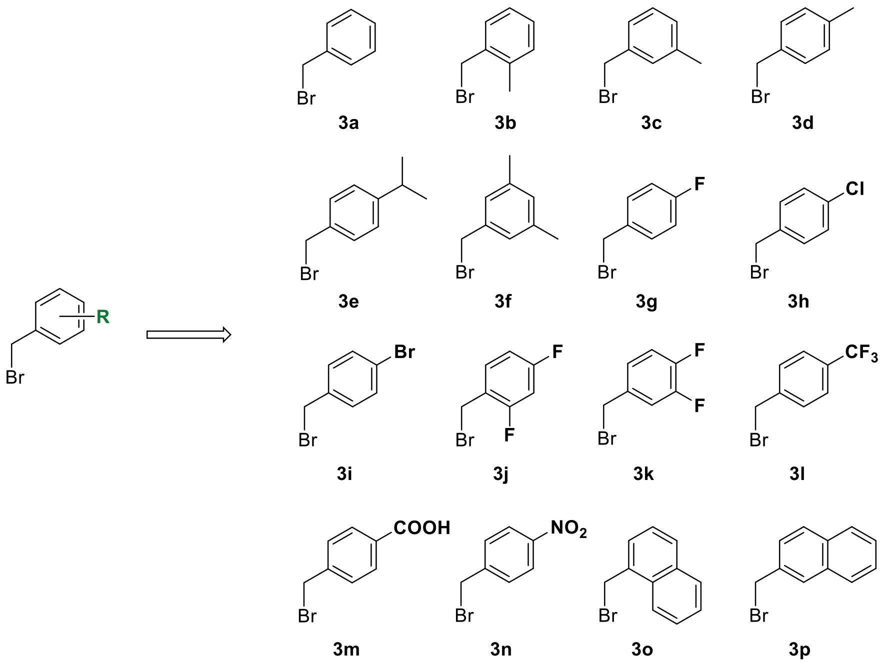

Figure S1. Structures of benzyl bromides used in this work.

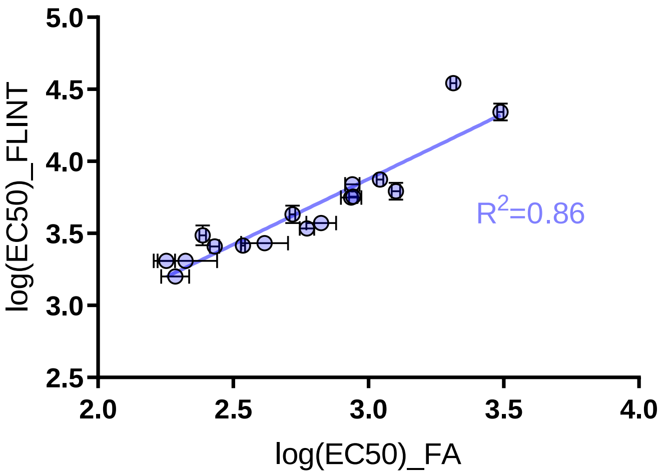

Figure S2. Correlation between the EC_{50} values determined from PyFLINT-B versus fluorescence anisotropy (FA) assay.

m^{7}GpppG

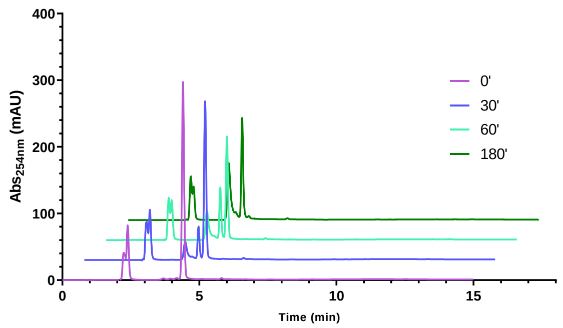

Bn^{7}GpppG (1a)

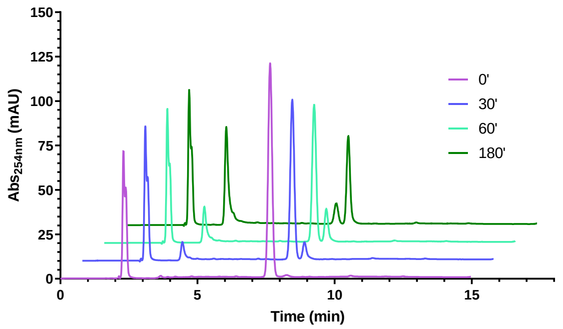

2-MeBn^{7}GpppG (1b)

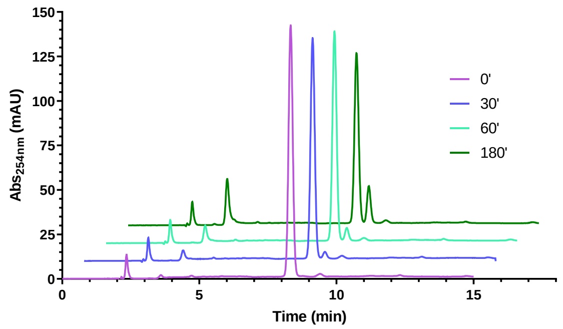

3-MeBn^{7}GpppG (1c)

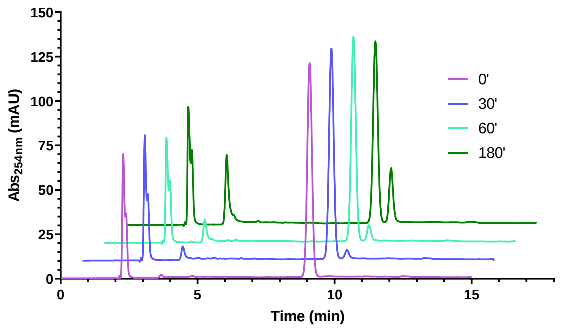

4-MeBn^{7}GpppG (1d)

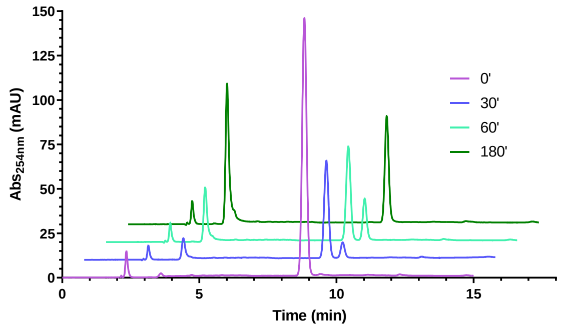

4-*i*PrBn^{7}GpppG (1e)

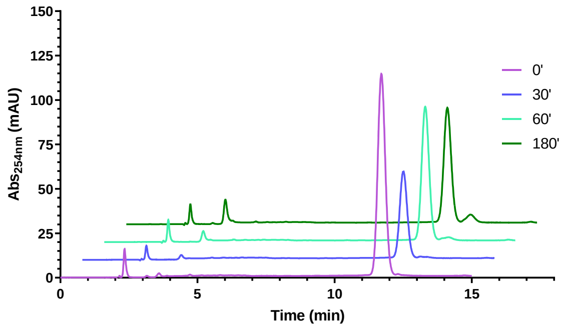

3,5-di-MeBn^{7}GpppG (1f)

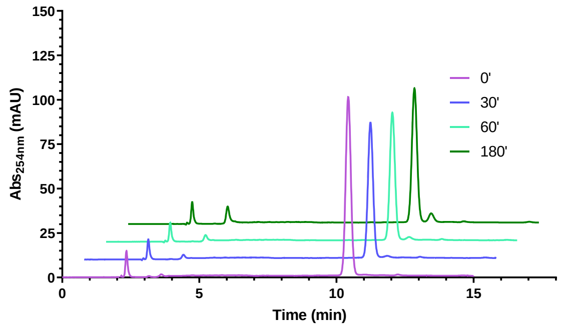

4-F-Bn^{7}GpppG (1g)

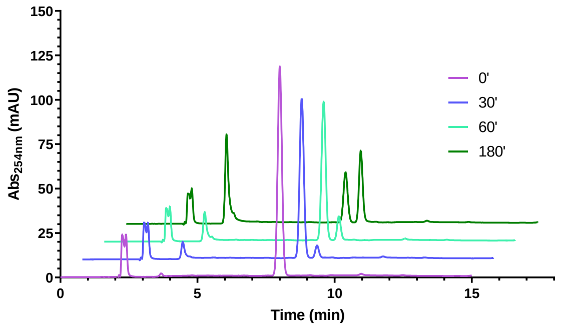

4-Cl-Bn^{7}GpppG (1h)

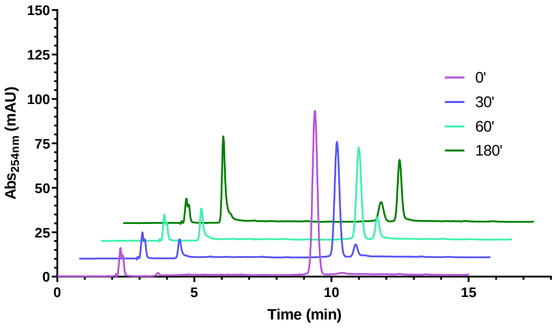

4-Br-Bn^{7}GpppG (1i)

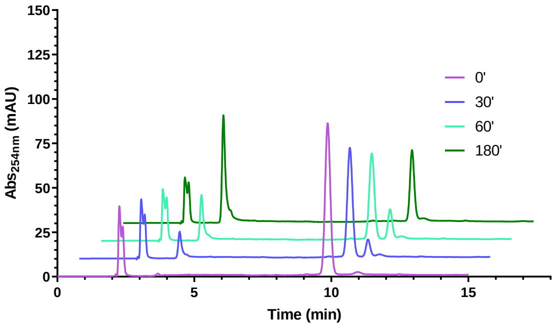

2,4-di-F-Bn^{7}GpppG (1j)

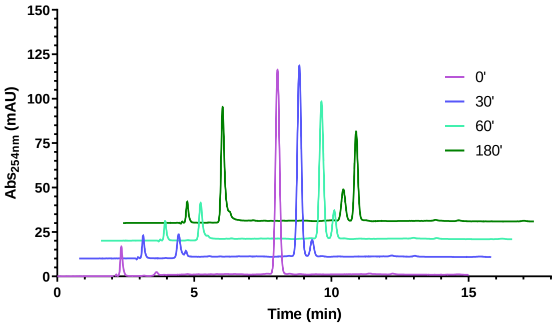

3,4-di-F-Bn^{7}GpppG (1k)

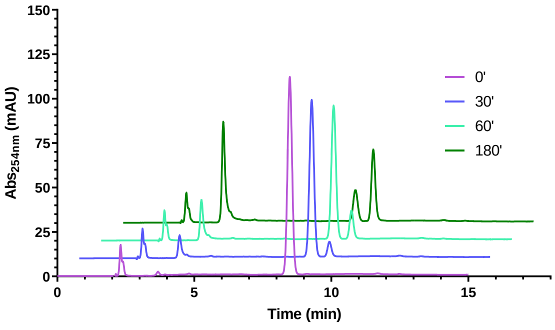

4-CF_3-Bn^{7}GpppG (1l)

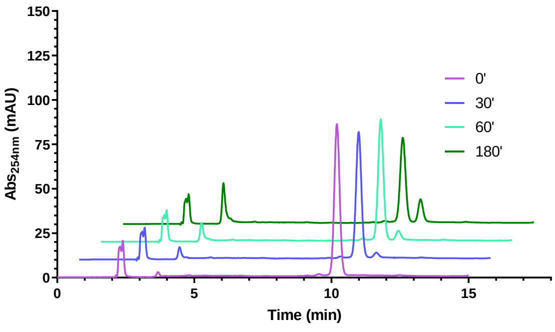

4-COOH-Bn^{7}GpppG (1m)

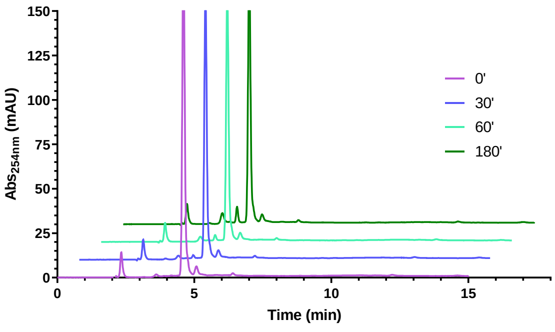

4-NO_2-Bn^{7}GpppG (1n)

α-Naphm^{7}GpppG (1o)

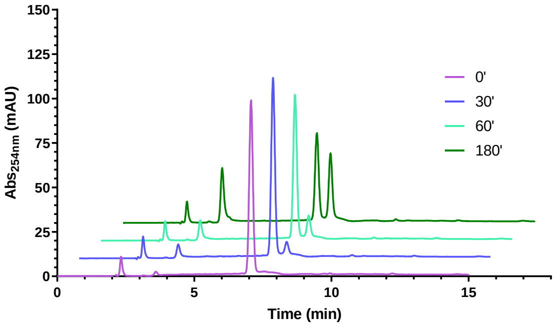

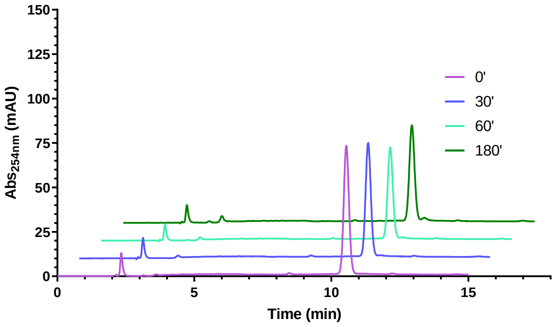

β-Naphm^{7}GpppG (1p)

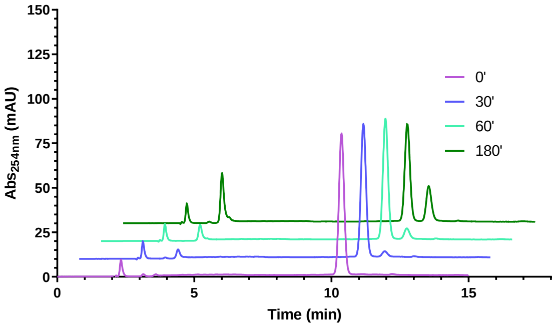

Bn^{7}G$_m$pppG (1q)

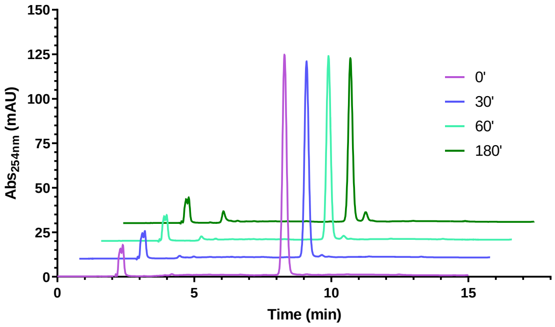

4-Cl-Bn^{7}G$_m$pppG (1r)

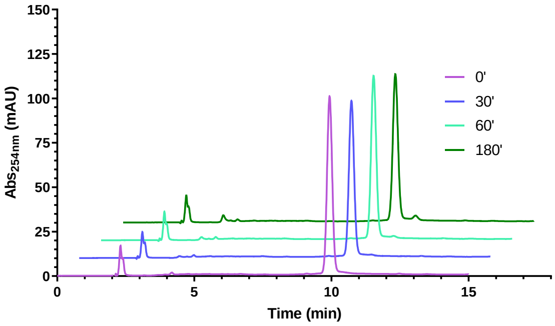

Figure S3. Analysis of cap analog susceptibility to hydrolysis by DcpS using RP-HPLC.

.

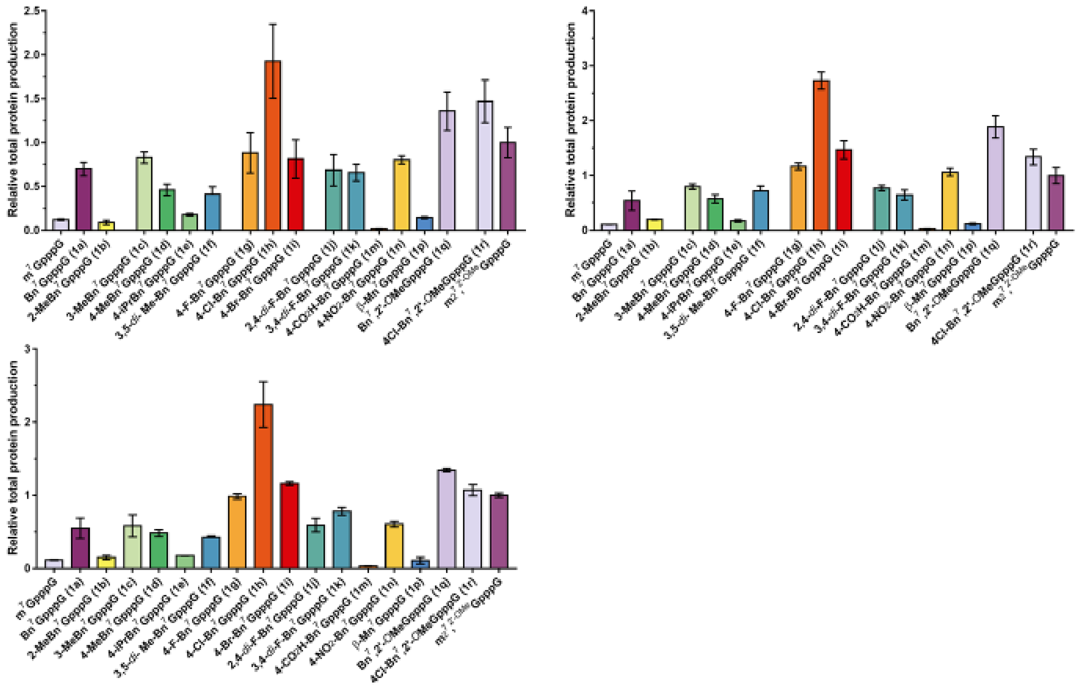

Figure S4. Relative total protein expression in three independents experiments. Data are normalized to m^7G_mpppG-RNA ± SD.

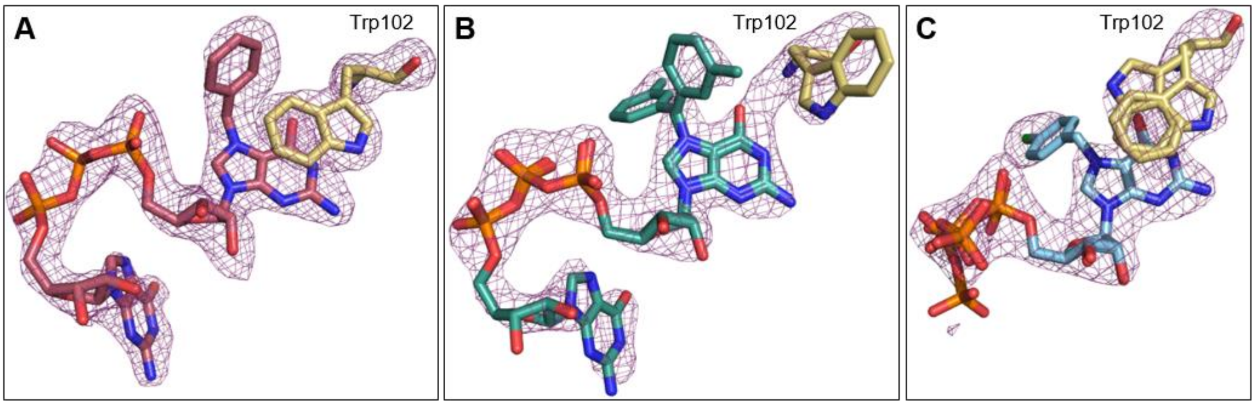

Figure S5. Simulated annealing omit map (F_0-F_c) contoured at 3σ around compound **1a** (A), **1c** (B), **1h** (C) and Trp102 from the solved X-ray structures of eIF4E in complex with cap analog

S3. Chemical synthesis of novel Gp3G cap analogs

1a) Synthesis of Bn7GpppG

P1-(7-benzylguanosin-5′-yl) P3-5′-guanosine

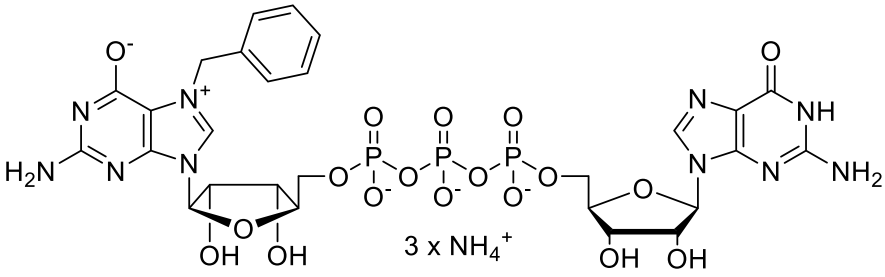

Bn7GpppG (**1a**) was prepared according to GP-B starting from Bn7GMP/TEA+ (**4a**) (109 mg, 500 mOD, 0.046 mmol), DMSO (8.0 ml), GDP-Im/Na+ (**5a**) (23 mg, 500 mOD, 0.043 mmol) and $ZnCl_2$ (47 mg, 0.344 mmol) yielding 14.86 mg of **1a** ammonium salt (507 mOD, 0.022 mmol, 51%). Reaction time: 24h.

^{1}H NMR (500 MHz, D_2O) δ = 9.37 (s, 1H), 8.06 (s, 1H), 7.38 – 7.28 (m, 5H), 5.93 (d, *J* = 3.7 Hz, 1H), 5.78 (d, *J* = 5.9 Hz, 1H), 5.57 (s, 2H), 4.66 (dd, *J* = 5.9, 5.2 Hz, 1H), 4.65 (dd, *J* = 5.0, 3.7 Hz, 1H), 4.49 (t, *J* = 5.0 Hz, 1H), 4.46 (dd, *J* = 5.2, 3.3 Hz, 1H), 4.43 – 4.35 (m, 2H), 4.33 – 4.18 (m, 4H);

^{31}P NMR (202 MHz, D_2O) δ = -10.29 – -10.73 (m, 2P), -22.15 (t, *J* = 19.4 Hz, 1P)

HRMS ESI (-) calcd. m/z for $C_{27}H_{32}N_{10}O_{18}P_3^-$ [M-H]$^-$: 877.11144, found 877.11251

1b) Synthesis of 2-MeBn7GpppG

P1-(7-(2-methylbenzyl)guanosin-5′-yl) P3-5′-guanosine

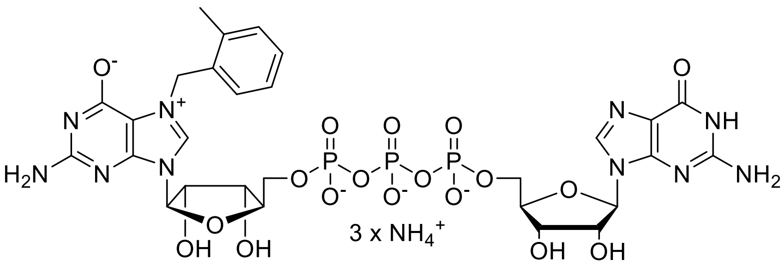

2-MeBn7GpppG (**1b**) was prepared according to GP-B starting from 2-MeBn7GMP/TEA+ (**4b**) (69 mg, 250 mOD, 0.025 mmol), DMSO (10.0 ml), GDP-Im/Na+ (**5a**) (12 mg, 250 mOD, 0.022 mmol) and $ZnCl_2$ (24 mg, 0.176 mmol) yielding 7.07 mg of **1b** ammonium salt (193 mOD, 0.0086 mmol, 39%). Reaction time: 24h.

^{1}H NMR (500 MHz, D_2O) δ = 9.21 (s, 1H), 8.13 (s, 1H), 7.20 – 6.86 (m, 4H), 5.94 (d, *J* = 3.9 Hz, 1H), 5.81 (d, *J* = 5.8 Hz, 1H), 5.58 (s, 2H), 4.71 (dd, *J* = 5.1, 3.9 Hz, 1H), 4.66 (dd, *J* = 5.8, 5.1 Hz, 1H), 4.51 (t, *J* = 5.1, 1H), 4.47 (dd, *J* = 5.1, 3.5 Hz, 1H), 4.42 – 4.32 (m, 2H), 4.32 – 4.17 (m, 4H), 2.31 (s, 3H);

^{31}P NMR (202 MHz, D_2O) δ = -10.32 – -10.72 (m, 2P), -22.72 – -22.47 (s, 1P)

HRMS ESI (-) calcd. m/z for $C_{28}H_{34}N_{10}O_{18}P_3^-$ [M-H]$^-$: 891.12709, found 891.12841

1c) Synthesis of 3-MeBn⁷GpppG

P1-(7-(3-methylbenzyl)guanosin-5′-yl) P3-5′-guanosine

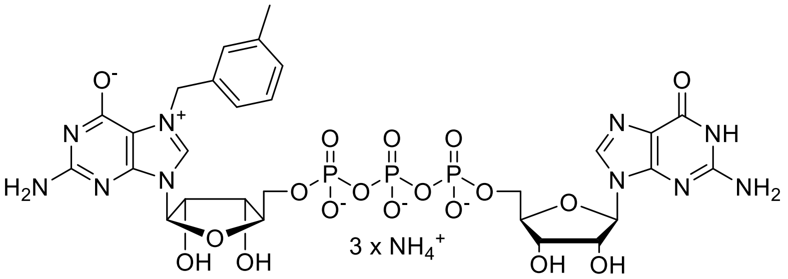

3-MeBn^{7}GpppG (**1c**) was prepared according to GP-B starting from 3-MeBn^{7}GMP/TEA$^+$ (**4c**) (56 mg, 500 mOD, 0.049 mmol), DMSO (8.0 ml), GDP-Im/Na$^+$ (**5a**) (23 mg, 500 mOD, 0.043 mmol) and $ZnCl_2$ (71 mg, 0.516 mmol) yielding 17.33 mg of **1c** ammonium salt (355 mOD, 0.016 mmol, 36%). Reaction time: 24h.

^{1}H NMR (500 MHz, D_2O) δ = 9.38 (s, 1H), 8.11 (s, 1H), 7.18 – 7.02 (m, 4H), 5.94 (d, *J* = 3.7 Hz, 1H), 5.78 (d, *J* = 5.6 Hz, 1H), 5.50 (s, 2H), 4.67 (dd, *J* = 5.0, 3.7 Hz, 1H), 4.65 (dd, *J* = 5.6, 5.1 Hz, 1H, overlapped with 4.67 (dd)), 4.51 (t, *J* = 5.0 Hz, 1H), 4.47 (dd, *J* = 5.0, 3.5 Hz, 1H), 4.42 – 4.37 (m, 2H), 4.32 – 4.19 (m, 4H), 2.22 (s, 3H);

^{31}P NMR (202 MHz, D_2O) δ = -10.25 – -10.65 (m, 2P), -21.66 – -22.55 (m, 1P)

HRMS ESI (-) calcd. m/z for $C_{28}H_{34}N_{10}O_{18}P_3^-$ [M-H]$^-$: 891.12709, found 891.12828

1d) Synthesis of 4-MeBn⁷GpppG

P1-(7-(4-methylbenzyl)guanosin-5′-yl) P3-5′-guanosine

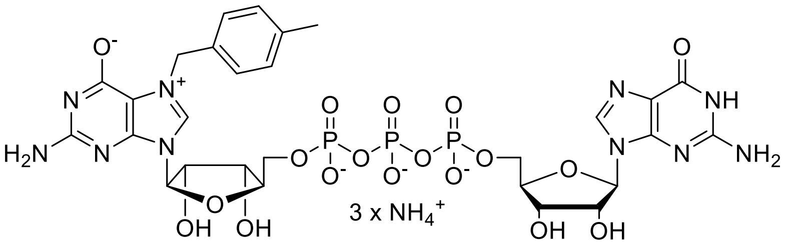

4-MeBn^{7}GpppG (**1d**) was prepared according to GP-B starting from 4-MeBn^{7}GMP/TEA$^+$ (**4d**) (41 mg, 200 mOD, 0.019 mmol), DMSO (8.0 ml), GDP-Im/Na$^+$ (**5a**) (12 mg, 250 mOD, 0.022 mmol) and $ZnCl_2$ (24 mg, 0.176 mmol) yielding 6.75 mg of **1d** ammonium salt (243 mOD, 0.011 mmol, 58%). Reaction time: 24h.

^{1}H NMR (500 MHz, D_2O) δ = 9.37 (s, 1H), 8.08 (s, 1H), 7.21 (d, *J* = 7.8 Hz, 2H), 7.06 (d, *J* = 7.8 Hz, 2H), 5.93 (d, *J* = 3.7 Hz, 1H), 5.76 (d, *J* = 5.7 Hz, 1H), 5.49 (s, 2H), 4.65 (dd, *J* = 5.0, 3.7 Hz, 1H), 4.64 (dd, *J* = 5.7, 5.1 Hz, 1H, overlapped with 4.65 (dd)), 4.50 (dd, *J* = 5.0, 5.0 Hz, 1H), 4.47 (dd, *J* = 5.1, 3.4 Hz, 1H), 4.41 – 4.37 (m, 2H), 4.32 – 4.19 (m, 4H), 2.20 (s, 3H);

^{31}P NMR (202 MHz, D_2O) δ = -10.23 – -10.65 (m, 2P), -21.67 – -22.55 (s, 1P)

HRMS ESI (-) calcd. m/z for $C_{28}H_{34}N_{10}O_{18}P_3^-$ [M-H]$^-$: 891.12709, found 891.12867

1e) Synthesis of 4-*i*PrBn⁷GpppG

P1-(7-(4-isopropylbenzyl)guanosin-5′-yl) P3-5′-guanosine

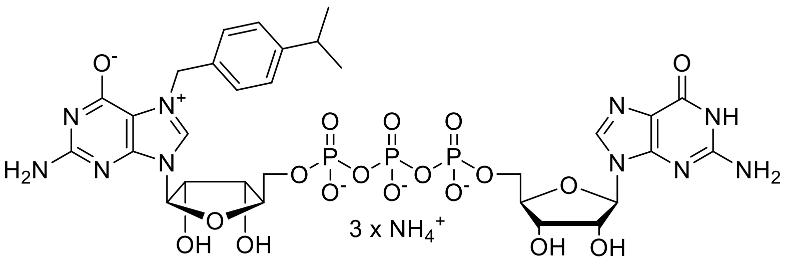

4-iPrBn⁷GpppG (**1e**) was prepared according to GP-B starting from 4-iPrBn⁷GMP/TEA⁺ (**4e**) (37 mg, 250 mOD, 0.023 mmol), DMSO (10.0 ml), GDP-Im/Na⁺ (**5a**) (12 mg, 250 mOD, 0.022 mmol) and $ZnCl_2$ (24 mg, 0.176 mmol) yielding 8.69 mg of **1e** ammonium salt (206 mOD, 0.0092 mmol, 42%). Reaction time: 24h.

^{1}H NMR (500 MHz, D_2O) δ = 9.42 (s, 1H), 8.17 (s, 1H), 7.25 (d, *J* = 8.0 Hz, 2H), 7.08 (d, *J* = 8.0 Hz, 2H), 5.94 (d, *J* = 3.7 Hz, 1H), 5.80 (d, *J* = 5.4 Hz, 1H), 5.53 (s, 2H), 4.65 (dd, *J* = 5.0, 3.7 Hz, 1H), 4.60 (t, *J* = 5.4, 1H), 4.51 (t, *J* = 5.0 Hz, 1H), 4.46 (dd, *J* = 5.3, 3.6 Hz, 1H), 4.42 – 4.37 (m, 2H), 4.32 – 4.20 (m, 4H), 2.72 (hept, *J* = 6.9 Hz, 1H), 1.06 (d, *J* = 6.9 Hz, 3H), 1.05 (d, *J* = 6.9 Hz, 3H, overlapped with 1.06 (d));

^{31}P NMR (202 MHz, D_2O) δ = -10.28 – -10.60 (m, 2P), -22.04 (t, *J* = 19.2 Hz, 1P)

HRMS ESI (-) calcd. m/z for $C_{30}H_{38}N_{10}O_{18}P_3^-$ $[M-H]^-$: 915.15839, found 919.15983

1f) Synthesis of 3,5-di-MeBn⁷GpppG

P1-(7-(3,5-dimethylbenzyl)guanosin-5′-yl) P3-5′-guanosine

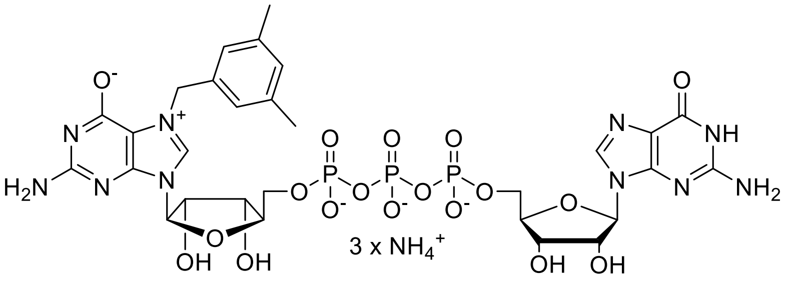

3,5-diMeBn⁷GpppG (**1f**) was prepared according to GP-B starting from 3,5-diMeBn⁷GMP/TEA⁺ (**4f**) (11 mg, 250 mOD, 0.025 mmol), DMSO (10.0 ml), GDP-Im/Na⁺ (**5a**) (12 mg, 250 mOD, 0.022 mmol) and $ZnCl_2$ (24 mg, 0.176 mmol) yielding 14.61 mg of **1f** ammonium salt (307 mOD, 0.014 mmol, 62%). Reaction time: 24h.

^{1}H NMR (500 MHz, D_2O) δ = 9.39 (s, 1H), 8.10 (s, 1H), 6.91 (s, 2H), 6.80 (s, 1H), 5.93 (d, *J* = 3.8 Hz, 1H), 5.77 (d, *J* = 5.6 Hz, 1H), 5.45 (s, 2H), 4.66 (dd, *J* = 4.9, 3.8 Hz, 1H), 4.64 (dd, *J* = 5.6, 5.1 Hz, 1H), 4.51 (t, *J* = 5.0, 5.0 Hz, 1H), 4.47 (dd, *J* = 5.1, 3.5 Hz, 1H), 4.42 – 4.37 (m, 2H), 4.32 – 4.20 (m, 4H), 2.14 (s, 6H);

^{31}P NMR (202 MHz, D_2O) δ = -10.27 – -10.64 (m, 2P), -22.11 (t, *J* = 19.3 Hz, 1P)

HRMS ESI (-) calcd. m/z for $C_{29}H_{36}N_{10}O_{18}P_3^-$ $[M-H]^-$: 905.14274, found 905.14393

1g) Synthesis of 4-F-Bn7GpppG

P1-(7-(4-fluorobenzyl)guanosin-5′-yl) P3-5′-guanosine

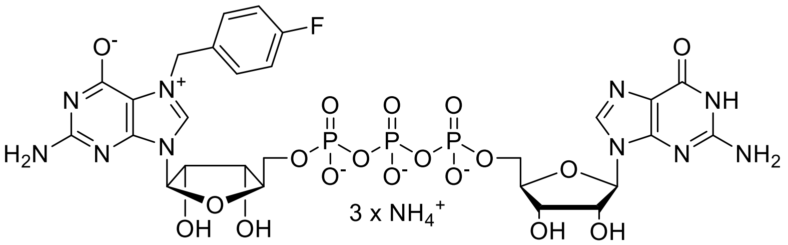

4-F-Bn7GpppG (**1g**) was prepared according to GP-B starting from 4-F-Bn7GMP/TEA+ (**4g**) (77 mg, 500 mOD, 0.046 mmol), DMSO (8.0 ml), GDP-Im/Na+ (**5a**) (23 mg, 500 mOD, 0.043 mmol) and $ZnCl_2$ (47 mg, 0.344 mmol) yielding 16.83 mg of **1g** ammonium salt (432 mOD, 0.019 mmol, 44%). Reaction time: 24h.

^{1}H NMR (500 MHz, D_2O) δ = 9.40 (s, 1H), 8.16 (s, 1H), 7.42 – 7.36 (m, 2H), 7.05 – 6.97 (m, 2H), 5.94 (d, *J* = 3.7 Hz, 1H), 5.79 (d, *J* = 5.7 Hz, 1H), 5.54 (s, 2H), 4.67 (dd, *J* = 5.7, 5.3 Hz, 1H), 4.65 (dd, *J* = 4.9, 3.7 Hz, 1H, overlapped with 4.67 (dd)), 4.50 (dd, *J* = 5.0, 4.9 Hz, 1H), 4.47 (dd, *J* = 5.3, 3.5 Hz, 1H), 4.42 – 4.37 (m, 2H), 4.32 – 4.20 (m, 4H);

^{31}P NMR (202 MHz, D_2O) δ = -10.30 – -10.63 (m, 2P), -22.14 (t, *J* = 19.3 Hz, 1P)

^{19}F NMR (471 MHz, D_2O) δ = -113.35 – -113.43 (m, 1F)

HRMS ESI (-) calcd. m/z for $C_{27}H_{31}FN_{10}O_{18}P_3^-$ [M-H]$^-$: 895.10202, found 895.10312

1h) Synthesis of 4-Cl-Bn7GpppG

P1-(7-(4-chlorobenzyl)guanosin-5′-yl) P3-5′-guanosine

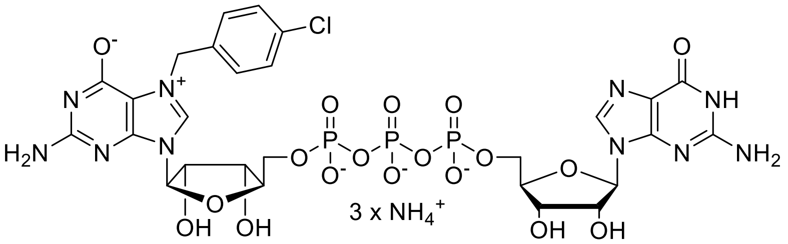

4-Cl-Bn7GpppG (**1h**) was prepared according to GP-B starting from 4-Cl-Bn7GMP/TEA+ (**4h**) (206 mg, 1000 mOD, 0.095 mmol), DMSO (10.0 ml), GDP-Im/Na+ (**5a**) (46 mg, 1000 mOD, 0.086 mmol) and $ZnCl_2$ (94 mg, 0.688 mmol) yielding 31.27 mg of **1h** ammonium salt (442 mOD, 0.020 mmol, 22%). Reaction time: 24h.

^{1}H NMR (500 MHz, D_2O) δ = 9.46 (s, 1H), 8.10 (s, 1H), 7.30 (d, *J* = 8.4 Hz, 2H), 7.22 (d, *J* = 8.4 Hz, 2H), 5.96 (d, *J* = 3.7 Hz, 1H), 5.77 (d, *J* = 5.8 Hz, 1H), 5.54 (s, 2H), 4.67 (dd, *J* = 5.8, 5.1 Hz, 1H), 4.66 (dd, *J* = 5.0, 3.7 Hz, 1H, overlapped with 4.67 (dd)), 4.50 (dd, *J* = 5.0, 5.0 Hz, 1H), 4.46 (dd, *J* = 5.1, 3.4 Hz, 1H), 4.42 – 4.37 (m, 2H), 4.32 – 4.20 (m, 4H);

^{31}P NMR (202 MHz, D_2O) δ = -10.21 – -10.64 (m, 2P), -22.73 – 22.61 (m, 1P)

HRMS ESI (-) calcd. m/z for $C_{27}H_{31}ClN_{10}O_{18}P_3^-$ [M-H]$^-$: 911.07247, found 911.07402

1i) Synthesis of 4-Br-Bn7GpppG

P1-(7-(4-bromobenzyl)guanosin-5′-yl) P3-5′-guanosine

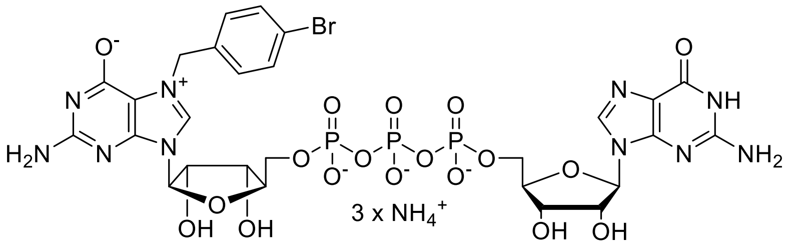

4-Br-Bn7GpppG (**1i**) was prepared according to GP-B starting from 4-Br-Bn7GMP/TEA+ (**4i**) (28 mg, 333 mOD, 0.031 mmol), DMSO (8.0 ml), GDP-Im/Na+ (**5a**) (23 mg, 500 mOD, 0.043 mmol) and $ZnCl_2$ (47 mg, 0.344 mmol) yielding 19.93 mg of **1i** ammonium salt (372 mOD, 0.017 mmol, 56%). Reaction time: 24h.

^{1}H NMR (500 MHz, D_2O) δ = 9.48 (s, 1H), 8.18 (s, 1H), 7.27 (d, *J* = 8.5 Hz, 2H), 7.18 (d, *J* = 8.5 Hz, 2H), 5.97 (d, *J* = 3.5 Hz, 1H), 5.79 (d, *J* = 5.4 Hz, 1H), 5.52 (d, *J* = 15.2 Hz, 1H), 5.48 (d, *J* = 15.2 Hz, 1H), 4.66 (dd, *J* = 4.8, 3.5 Hz, 1H), 4.63 (dd, *J* = 5.4, 5.4 Hz, 1H, overlapped with 4.66 (dd)), 4.51 (t, 4.8 Hz, 1H), 4.46 (dd, *J* = 5.4, 3.8 Hz, 1H), 4.43 – 4.38 (m, 2H), 4.32 – 4.20 (m, 4H);

^{31}P NMR (202 MHz, D_2O) δ = -10.06 – -10.72 (m, 2P), -21.59 – -22.58 (m, 1P)

HRMS ESI (-) calcd. m/z for $C_{27}H_{31}BrN_{10}O_{18}P_3^-$ [M-H]$^-$: 955.02195, found 955.02287

1j) Synthesis of 2,4-di-F-Bn7GpppG

P1-(7-(2,4-difluorobenzyl)guanosin-5′-yl) P3-5′-guanosine

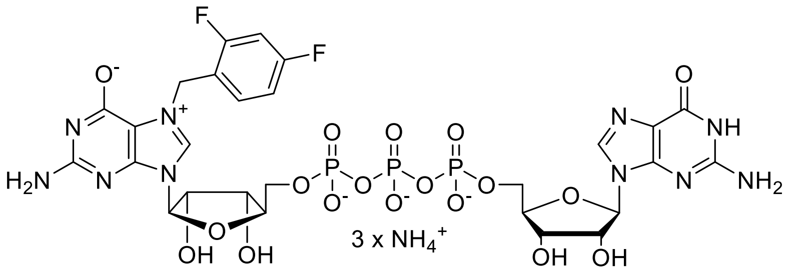

2,4-di-F-Bn7GpppG (**1j**) was prepared according to GP-B starting from 2,4-di-F-Bn7GMP/TEA+ (**4j**) (55 mg, 250 mOD, 0.024 mmol), DMSO (8.0 ml), GDP-Im/Na+ (**5a**) (12 mg, 250 mOD, 0.022 mmol) and $ZnCl_2$ (24 mg, 0.176 mmol) yielding 7.05 mg of **1j** ammonium salt (178 mOD, 0.0079 mmol, 36%). Reaction time: 24h.

^{1}H NMR (500 MHz, D_2O) δ = 9.34 (s, 1H), 8.11 (s, 1H), 7.42 – 7.35 (m, 1H), 6.93 – 6.86 (m, 2H), 5.95 (d, *J* = 3.8 Hz, 1H), 5.81 (d, *J* = 6.0 Hz, 1H), 5.61 (s, 2H), 4.70 (dd, *J* = 6.0, 5.2 Hz, 1H), 4.68 (dd, *J* = 4.8, 3.8 Hz, 1H, overlapped with 4.70 (dd)), 4.51 – 4.48 (m, 2H), 4.41 – 4.36 (m, 2H), 4.32 – 4.20 (m, 4H);

^{31}P NMR (202 MHz, D_2O) δ = -10.23 – -10.73 (m, 2P), -22.18 (t, *J* = 19.3 Hz, 1P)

^{19}F NMR (471 MHz, D_2O) δ = -108.75 – -108.79 (m, 1F), -112.85 – -112.99 (m,1F)

HRMS ESI (-) calcd. m/z for $C_{27}H_{30}F_2N_{10}O_{18}P_3^-$ [M-H]$^-$: 913.09259, found 913.09407

1k) Synthesis of 3,4-di-F-Bn7GpppG

P1-(7-(3,4-difluorobenzyl)guanosin-5′-yl) P3-5′-guanosine

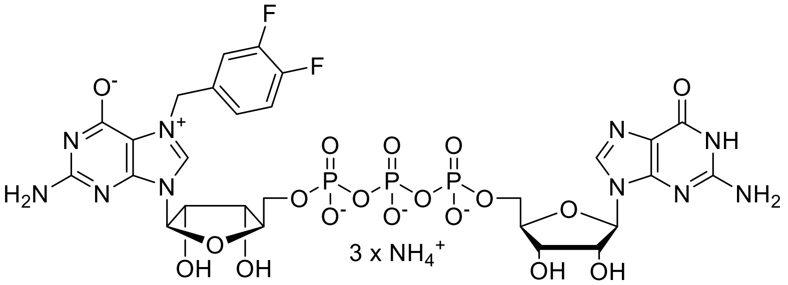

3,4-di-F-Bn7GpppG (**1k**) was prepared according to GP-B starting from 3,4-di-F-Bn7GMP/TEA+ (**4k**) (49 mg, 250 mOD, 0.025 mmol), DMSO (10.0 ml), GDP-Im/Na+ (**5a**) (12 mg, 250 mOD, 0.022 mmol) and $ZnCl_2$ (24 mg, 0.176 mmol) yielding 6.74 mg of **1k** ammonium salt (202 mOD, 0.0090 mmol, 41%). Reaction time: 24h.

^{1}H NMR (500 MHz, D_2O) δ = 9.43 (s, 1H), 8.15 (s, 1H), 7.35 – 7.29 (m, 1H), 7.21 – 7.13 (m, 2H), 5.95 (d, *J* = 3.7 Hz, 1H), 5.79 (d, *J* = 5.7 Hz, 1H), 5.55 (s, 2H), 4.67 (t, *J* = 5.7 Hz, 1H), 4.65 (dd, *J* = 4.9, 3.7 Hz, 1H, overlapped with 4.67 (t)), 4.50 (t, *J* = 4.9 Hz, 1H), 4.47 (dd, *J* = 5.7, 3.5 Hz, 1H, overlapped with 4.50 (t)), 4.42 – 4.37 (m, 2H), 4.31 – 4.20 (m, 4H);

^{31}P NMR (202 MHz, D_2O) δ = -10.04 – -10.69 (m, 2P), -21.63 – -22.66 (m, 1P);

^{19}F NMR (471 MHz, D_2O) δ = -137.58 – -137.71 (m, 1F), -138.23 – -138.35 (m, 1F);

^{19}F NMR {1H} (471 MHz, D_2O) δ = -137.65 (d, *J* = 21.4 Hz, 1F), -138.29 (d, *J* = 21.4 Hz, 1F);

HRMS ESI (-) calcd. m/z for $C_{27}H_{30}F_2N_{10}O_{18}P_3^-$ [M-H]$^-$: 913.09259, found 913.09409

1l) Synthesis of 4-CF3-Bn-GpppG

P1-(7-(4-trifluoromethylbenzyl)guanosin-5′-yl) P3-5′-guanosine

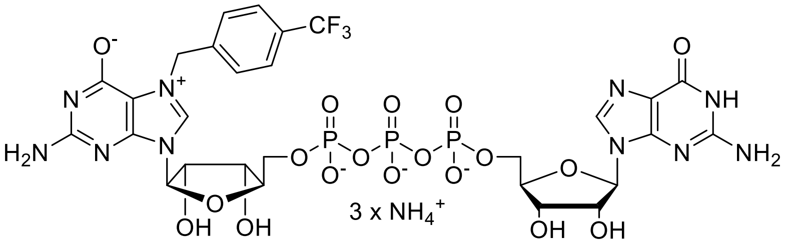

4-CF_3-Bn7GpppG (**1l**) was prepared according to GP-B starting from 4-CF_3-Bn7GMP/TEA+ (**4l**) (10 mg, 54.2 mOD, 0.0048 mmol), DMSO (1.0 ml), GDP-Im/Na+ (**5a**) (2 mg, 45.6 mOD, 0.004 mmol) and $ZnCl_2$ (14 mg, 0.10 mmol) yielding 1.5 mg of **1l** ammonium salt (29.4 mOD, 0.0013 mmol, 27%). Reaction time: 24h.

^{1}H NMR (500 MHz, D_2O) δ 8.01 (s, 1H), 7.63 (d, *J* = 8.2 Hz, 2H), 7.53 (d, *J* = 8.2 Hz, 2H), 5.96 (d, *J* = 4.3 Hz, 1H), 5.75 (d, *J* = 5.7 Hz, 1H), 5.70 (d, *J* = 15.4 Hz, 1H), 5.67 (d, *J* = 15.4 Hz, 1H), 4.68 (t, *J* = 5.7 Hz, 1H), 4.67 (t, *J* = 4.3 Hz, 1H, overlapped with 4.68 (t)), 4.50 (t, *J* = 5.0 Hz, 1H), 4.48 – 4.43 (m, 1H), 4.42 – 4.36 (m, 2H), 4.32 – 4.17 (m, 4H);

^{31}P NMR (202 MHz, D_2O) δ -10.15 – -10.78 (m), -22.20 (t, *J* = 19.7 Hz); ^{19}F NMR (471 MHz, D_2O) δ -62.49.

HRMS ESI (-) calcd. m/z for $C_{28}H_{31}F_3N_{10}O_{18}P_3^-$ [M-H]$^-$: 945.09882, found 945.09990

1m) Synthesis of 4-COOH-Bn⁷GpppG

P1-(7-(4-carboxylbenzyl)guanosin-5′-yl) P3-5′-guanosine

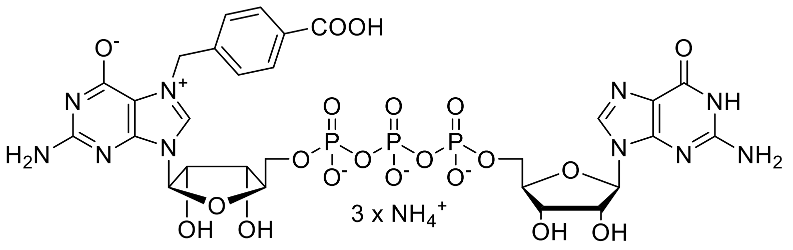

4-CO_2H-Bn^7GpppG (**1m**) was prepared according to GP-B starting from 4-CO_2H-Bn^7GMP/TEA^+ (**4m**) (38 mg, 500 mOD, 0.046 mmol), DMSO (10.0 ml), GDP-Im/Na^+ (**5a**) (23 mg, 500 mOD, 0.043 mmol) and $ZnCl_2$ (47 mg, 0.344 mmol) yielding 10.68 mg of **1m** ammonium salt (222 mOD, 0.010 mmol, 22%). Reaction time: 24h.

^{1}H NMR (500 MHz, D_2O) δ = 9.49 (s, 1H), 8.15 (s, 1H), 7.71 (d, *J* = 8.4 Hz, 2H), 7.32 (d, *J* = 8.4 Hz, 2H), 5.96 (d, *J* = 3.5 Hz, 1H), 5.75 (d, *J* = 5.4 Hz, 1H), 5.64 (d, *J* = 15.3 Hz, 1H), 5.59 (d, *J* = 15.3 Hz, 1H), 4.67 (dd, *J* = 5.0, 3.5 Hz, 1H), 4.61 (t, *J* = 5.4 Hz, 1H), 4.52 (t, *J* = 5.0 Hz, 1H), 4.45 (dd, *J* = 5.4, 3.5 Hz, 1H), 4.43 – 4.39 (m, 2H), 4.33 – 4.20 (m, 4H);

^{31}P NMR (202 MHz, D_2O) δ = -10.24 – -10.61 (m, 2P), -22.06 (t, *J* = 19.2 Hz, 1P)

HRMS ESI (-) calcd. m/z for $C_{28}H_{32}N_{10}O_{20}P_3^-$ $[M-H]^-$: 921.10127, found 921.10277

1n) Synthesis of 4-NO₂-Bn⁷GpppG

P1-(7-(4-nitrobenzyl)guanosin-5′-yl) P3-5′-guanosine

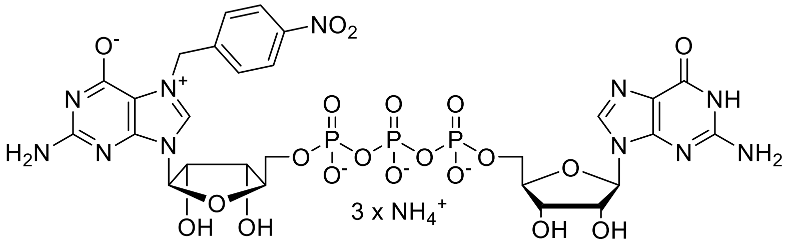

4-NO_2H-Bn^7GpppG (**1n**) was prepared according to GP-B starting from 4-NO_2H-Bn^7GMP/TEA^+ (**4n**) (86 mg, 500 mOD, 0.046 mmol), DMSO (8.0 ml), GDP-Im/Na^+ (**5a**) (23 mg, 500 mOD, 0.043 mmol) and $ZnCl_2$ (47 mg, 0.344 mmol) yielding 19.46 mg of **1n** ammonium salt (426 mOD, 0.019 mmol, 43%). Reaction time: 24h.

^{1}H NMR (500 MHz, D_2O) δ = 9.55 (s, 1H), 8.13 (s, 1H), 8.07 (d, *J* = 8.8 Hz, 2H), 7.54 (d, *J* = 8.8 Hz, 2H), 5.97 (d, *J* = 3.6 Hz, 1H), 5.75 (d, *J* = 5.4 Hz, 2H), 5.74 (d, *J* = 15.4 Hz, 1H), 5.70 (d, *J* = 15.4 Hz, 1H), 4.67 (dd, *J* = 4.9, 3.6 Hz, 1H), 4.65 (t, *J* = 5.4 Hz, 2H), 4.51 (t, *J* = 4.9 Hz, 1H), 4.45 (dd, *J* = 5.4, 3.4 Hz, 1H), 4.43 – 4.38 (m, 2H), 4.33 – 4.18 (m, 4H).

^{31}P NMR (202 MHz, D_2O) δ = -10.22 – -10.63 (m, 2P), -22.10 (t, *J* = 19.6 Hz, 1P)

HRMS ESI (-) calcd. m/z for $C_{27}H_{31}N_{11}O_{20}P_3^-$ $[M-H]^-$: 922.09652, found 922.09742

1o) Synthesis of α-Naphm⁷GpppG

P1-(7-(1-methylnaphthalene)guanosin-5′-yl) P3-5′-guanosine

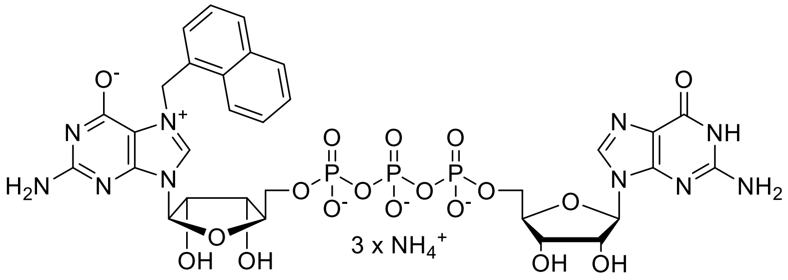

α-Mn⁷GpppG (**1o**) was prepared according to GP-B starting from α-Mn⁷GMP/TEA⁺ (**4o**) (40 mg, 1000 mOD, 0.098 mmol), DMSO (10.0 ml), GDP-Im/Na⁺ (**5a**) (46 mg, 1000 mOD, 0.086 mmol) and $ZnCl_2$ (72 mg, 0.528 mmol) yielding 44.00 mg of **1o** ammonium salt (1129 mOD, 0.050 mmol, 57%). Reaction time: 24h.

^{1}H NMR (500 MHz, D_2O) δ = 9.25 (s, 1H), 7.98 (s, 1H), 7.88 (d, J = 8.0 Hz, 1H), 7.65 (dd, J = 15.2, 8.0 Hz, 2H), 7.50 (t, J = 7.5 Hz, 1H), 7.41 (t, J = 7.5 Hz, 1H), 7.29 (t, J = 7.5 Hz, 1H), 7.19 (d, J = 7.5 Hz, 1H), 5.85 (q, J = 15.2 Hz, 2H), 5.82 (d, J = 3.7 Hz, 1H), 5.65 (d, J = 5.3 Hz, 1H), 4.58 (t, J = 3.7 Hz, 1H), 4.53 (t, J = 5.3 Hz, 1H), 4.48 (t, J = 5.3 Hz, 1H), 4.46 – 4.42 (m, 1H), 4.40 – 4.18 (m, 6H).

^{31}P NMR (202 MHz, D_2O) δ = δ -10.47 (m, 2P), -22.06 (m, 2P).

HRMS ESI (-) calcd. m/z for $C_{31}H_{34}N_{10}O_{18}P_3^-$ [M-H]$^-$: 927.12709, found 927.12841

1p) Synthesis of β-Naphm⁷GpppG

P1-(7-(2-methylnaphthalene)guanosin-5′-yl) P3-5′-guanosine

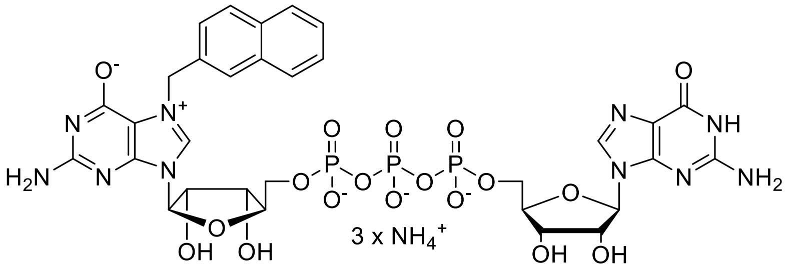

β-Mn⁷GpppG (**1p**) was prepared according to GP-B starting from β-Mn⁷GMP/TEA⁺ (**4p**) (67 mg, 250 mOD, 0.023 mmol), DMSO (8.0 ml), GDP-Im/Na⁺ (**5a**) (12 mg, 250 mOD, 0.022 mmol) and $ZnCl_2$ (24 mg, 0.176 mmol) yielding 6.71 mg of **1p** ammonium salt (223 mOD, 0.010 mmol, 45%). Reaction time: 24h.

^{1}H NMR (500 MHz, D_2O) δ = 9.49 (s, 1H), 8.02 (s, 1H), 7.64 (s, 1H), 7.56 – 7.50 (m, 3H), 7.34 – 7.27 (m, 3H), 5.82 (d, *J* = 3.8 Hz, 1H), 5.62 (d, *J* = 5.0 Hz, 1H), 5.53 (s, 2H), 4.59 (dd, *J* = 5.0, 3.7 Hz, 1H), 4.51 (dd, *J* = 3.8, 5.0 Hz, 1H), 4.48 (dd, *J* = 5.0, 5.0 Hz, 1H), 4.46 – 4.39 (m, 3H), 4.36 – 4.21 (m, 4H);

^{31}P NMR (202 MHz, D_2O) δ = -10.10 – -10.53 (m, 2P), -21.95 (t, *J* = 19.3 Hz, 1P)

HRMS ESI (-) calcd. m/z for $C_{31}H_{34}N_{10}O_{18}P_3^-$ [M-H]$^-$: 927.12709, found 927.12850

1q) Synthesis of Bn^{7}G$_{m}$pppG

P1-(2′O-methyl-7-benzylguanosin-5′-yl) P3-5′-guanosine

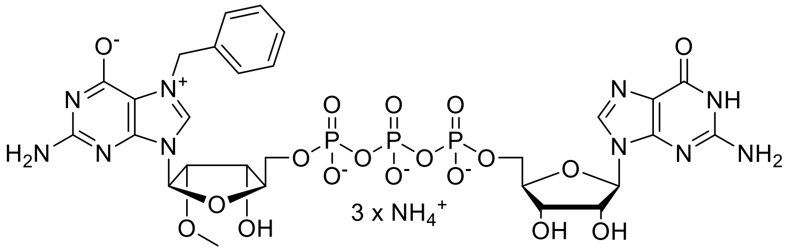

Bn$^{7,2'\text{-OMe}}$GpppG (**1q**) was prepared according to GP-B starting from Bn$^{7,2'\text{-OMe}}$GMP/TEA^{+} (**4q**) (250 mg, 500 mOD, 0.044 mmol), DMSO (8.0 ml), GDP-Im/Na^{+} (**5a**) (23 mg, 500 mOD, 0.043 mmol) and $ZnCl_2$ (71 mg, 0.516 mmol) yielding 16.26 mg of **1q** ammonium salt (328 mOD, 0.015 mmol, 33%). Reaction time: 24h.

^{1}H NMR (500 MHz, D_2O) δ = 9.33 (s, 1H), 8.04 (s, 1H), 7.39 – 7.25 (m, 5H), 5.93 (d, *J* = 2.8 Hz, 1H), 5.74 (d, *J* = 5.7 Hz, 1H), 5.56 (s, 2H), 4.64 (t, *J* = 5.7 Hz, 1H), 4.55 (dd, *J* = 6.3, 5.0 Hz, 1H), 4.45 (dd, *J* = 5.0, 3.0 Hz, 1H), 4.43 – 4.40 (m, 1H), 4.27 (dd, *J* = 5.0, 2.8 Hz, 1H, overlapped with 4.35 – 4.19 (m)), 4.35 – 4.19 (m, 5H), 3.59 (s, 3H);

^{31}P NMR (202 MHz, D_2O) δ = -10.37 – -10.69 (m, 2P), -22.13 (t, *J* = 19.3 Hz, 1P)

HRMS ESI (-) calcd. m/z for $C_{28}H_{34}N_{10}O_{18}P_3^-$ [M-H]$^-$: 891.12709, found 891.12836

1r) Synthesis of 4-Cl-Bn^{7}G$_{m}$pppG

P1-(2′O-methyl-7-(4-chlorobenzyl)guanosin-5′-yl) P3-5′-guanosine

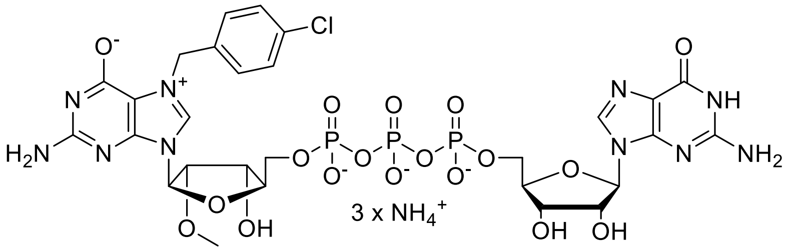

4-Cl-Bn$^{7,2'\text{-OMe}}$GpppG (**1r**) was prepared according to GP-B starting from 4-Cl-Bn$^{7,2'\text{-OMe}}$GMP/TEA^{+} (**4r**) (104 mg, 500 mOD, 0.046 mmol), DMSO (10.0 ml), GDP-Im/Na^{+} (**5a**) (23 mg, 500 mOD, 0.043 mmol) and $ZnCl_2$ (47 mg, 0.344 mmol) yielding 14.75 mg of **1r** ammonium salt (420 mOD, 0.019 mmol, 42%). Reaction time: 24h.

^{1}H NMR (500 MHz, D_2O) δ = 9.43 (s, 1H), 8.25 (s, 1H), 7.28 (d, *J* = 8.5 Hz, 2H), 7.18 (d, *J* = 8.5 Hz, 2H), 6.00 (d, *J* = 2.7 Hz, 1H), 5.78 (d, *J* = 5.3 Hz, 1H), 5.52 (s, 2H), 4.63 (t, *J* = 5.3 Hz, 1H), 4.58 (dd, *J* = 6.4, 5.0 Hz, 1H), 4.45 (dd, *J* = 5.0, 4.2 Hz, 1H), 4.42 (dd, *J* = 4.2, 2.7 Hz, 1H), 4.37 – 4.19 (m, 6H), 3.59 (s, 3H);

^{31}P NMR (202 MHz, D_2O) δ = -10.24 – -10.63 (m, 2P), -22.04 (t, *J* = 19.2 Hz, 1P)

HRMS ESI (-) calcd. m/z for $C_{28}H_{33}ClN_{10}O_{18}P_3^-$ [M-H]$^-$: 925.08812, found 925.08957

S4. Spectroscopic data: ^{1}H, ^{31}P, ^{19}F NMR, HRMS and UV/VIS spectra

Bn^{7}GpppG (**1a**)	
Structure	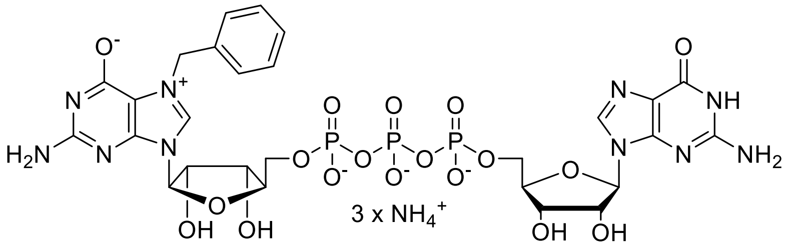
Reaction RP HPLC Profile	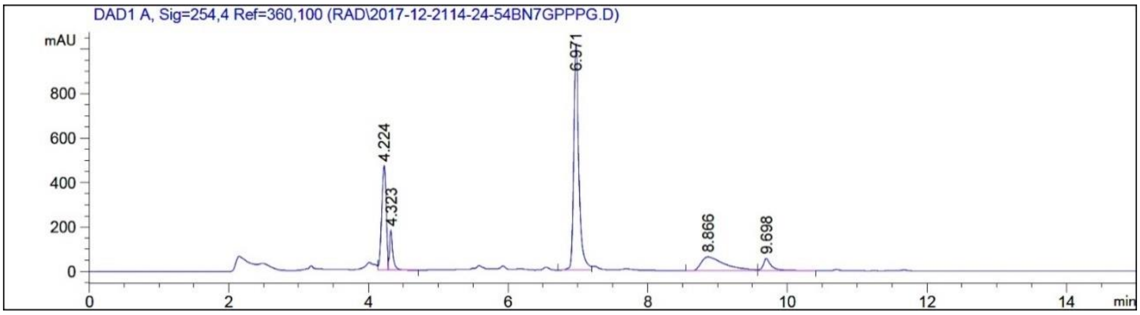
Purified Product RP HPLC Profile	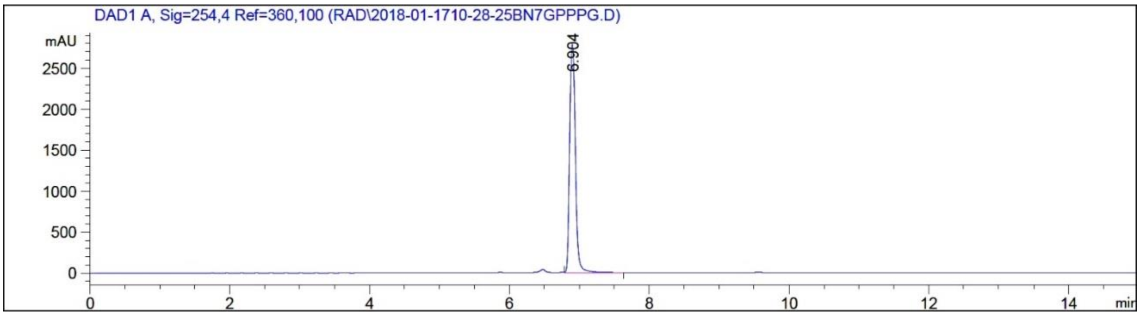

HRMS ES(-)	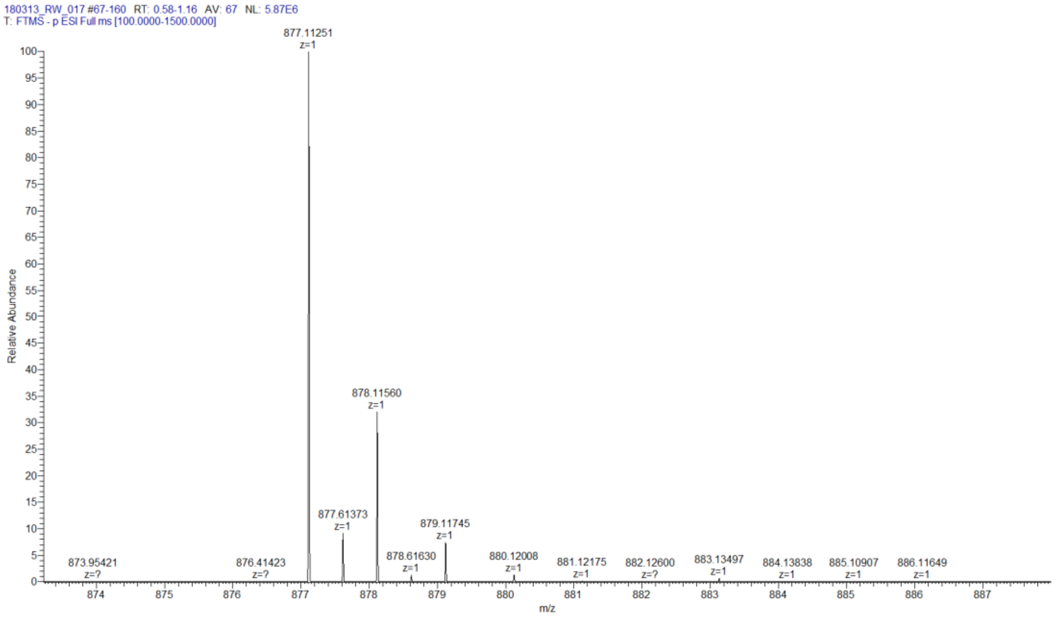
^{1}H NMR (500.24 MHz)	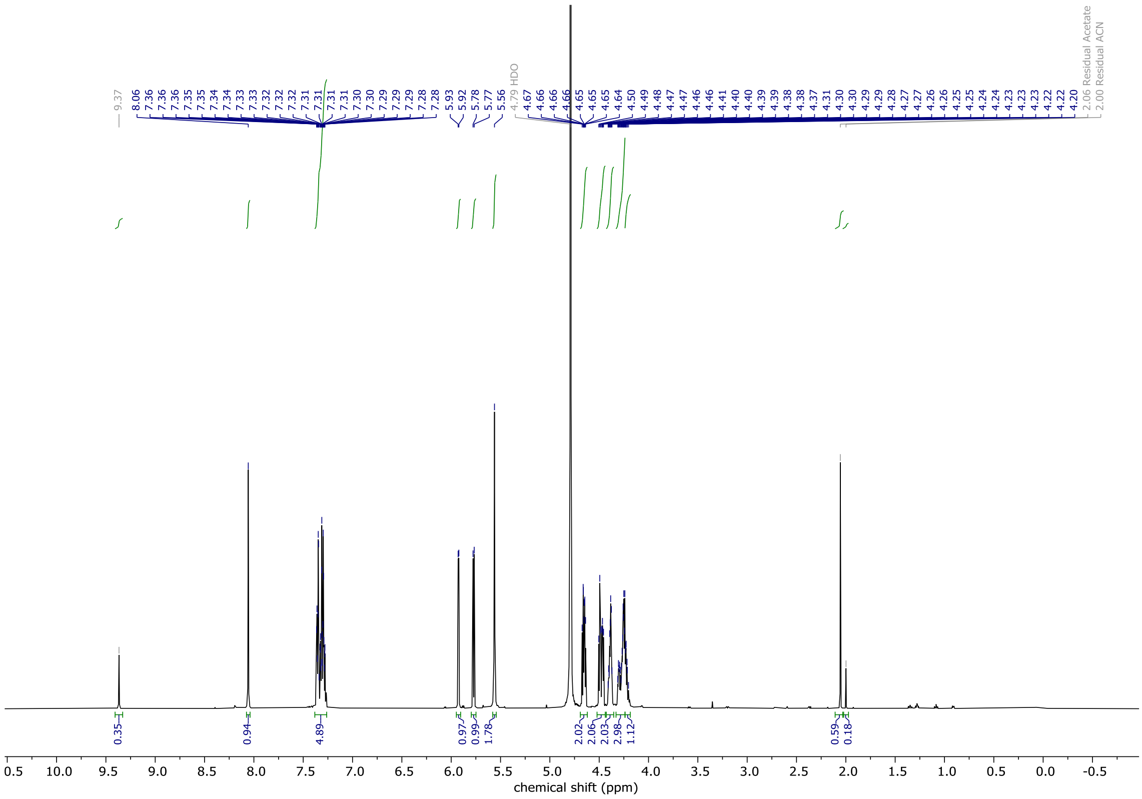

^{31}P NMR (202.49 MHz)	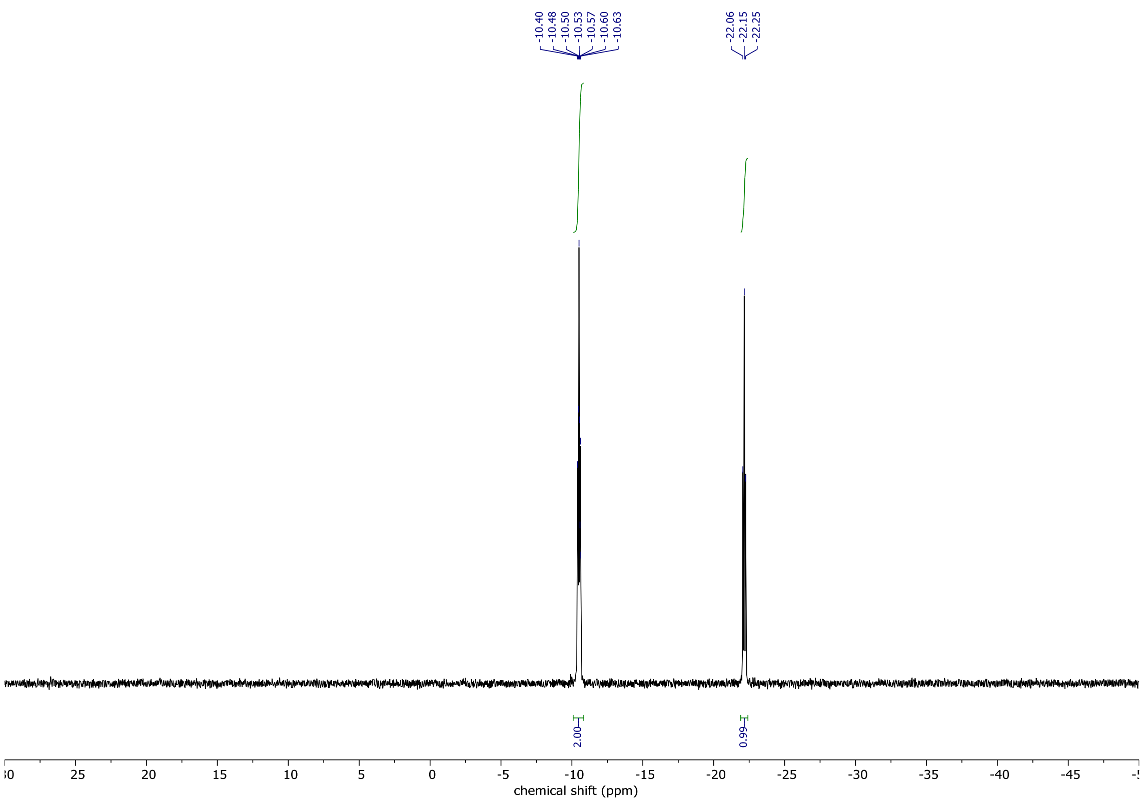
UV-VIS spectrum (recorded in 0.1M phosphate buffer pH	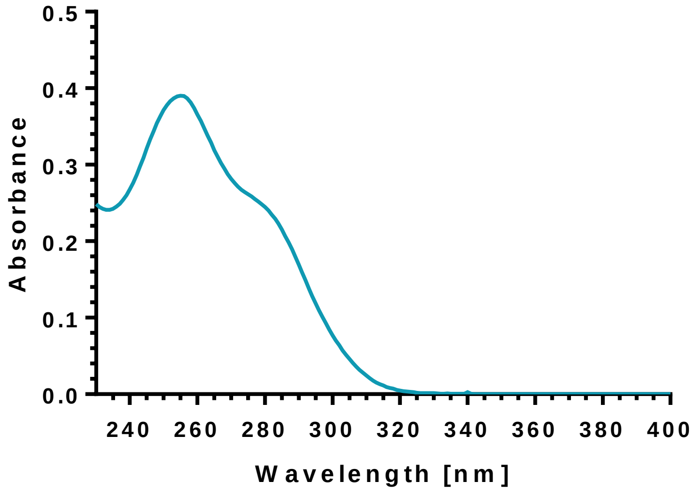

2-MeBn^{7}GpppG (**1b**)	
Structure	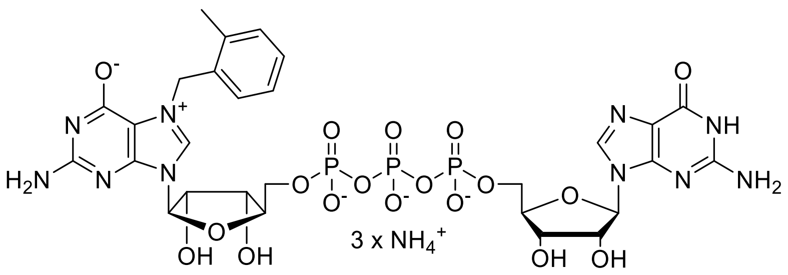
Reaction RP HPLC Profile	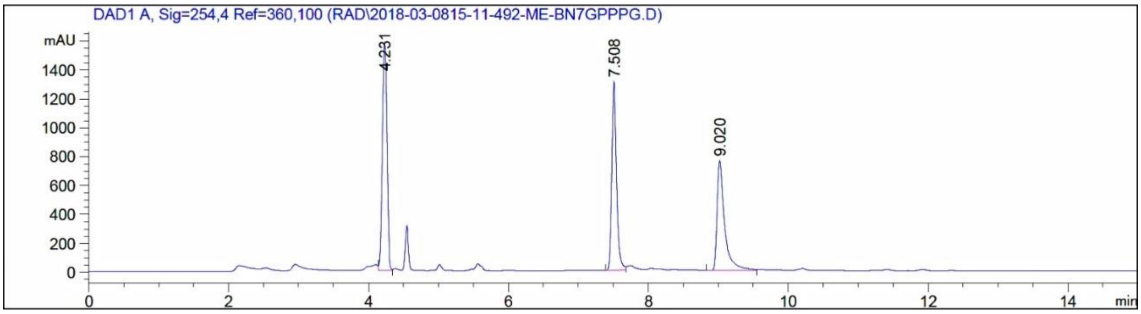
Purified Product RP HPLC Profile	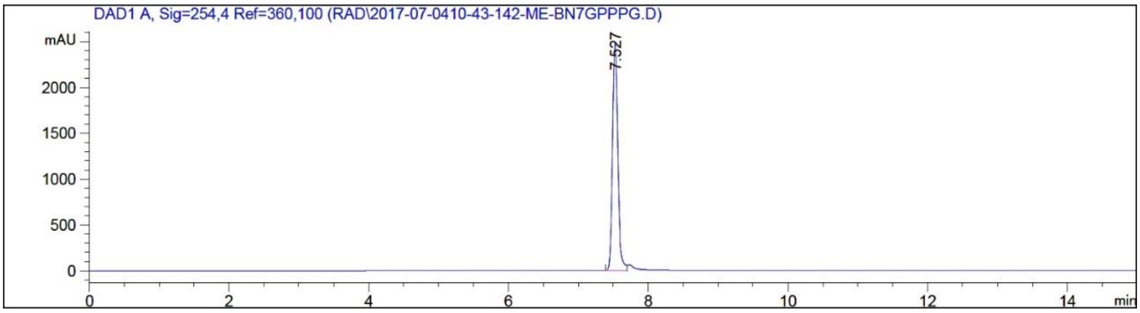
HRMS ES(-)	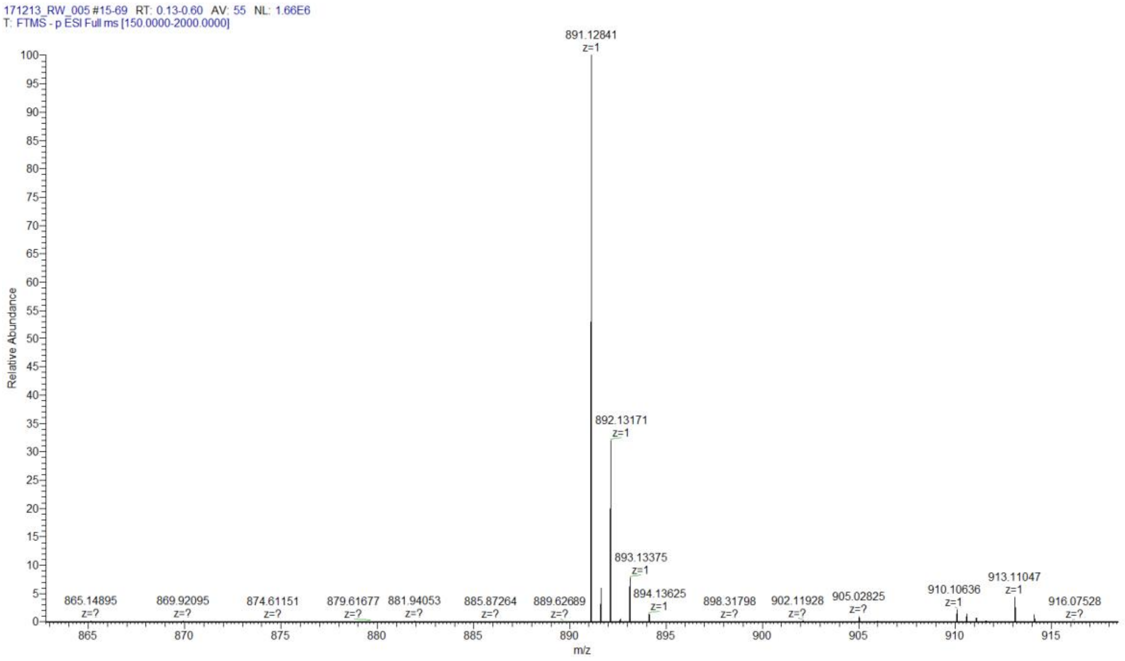

^{1}H NMR (500.24 MHz)

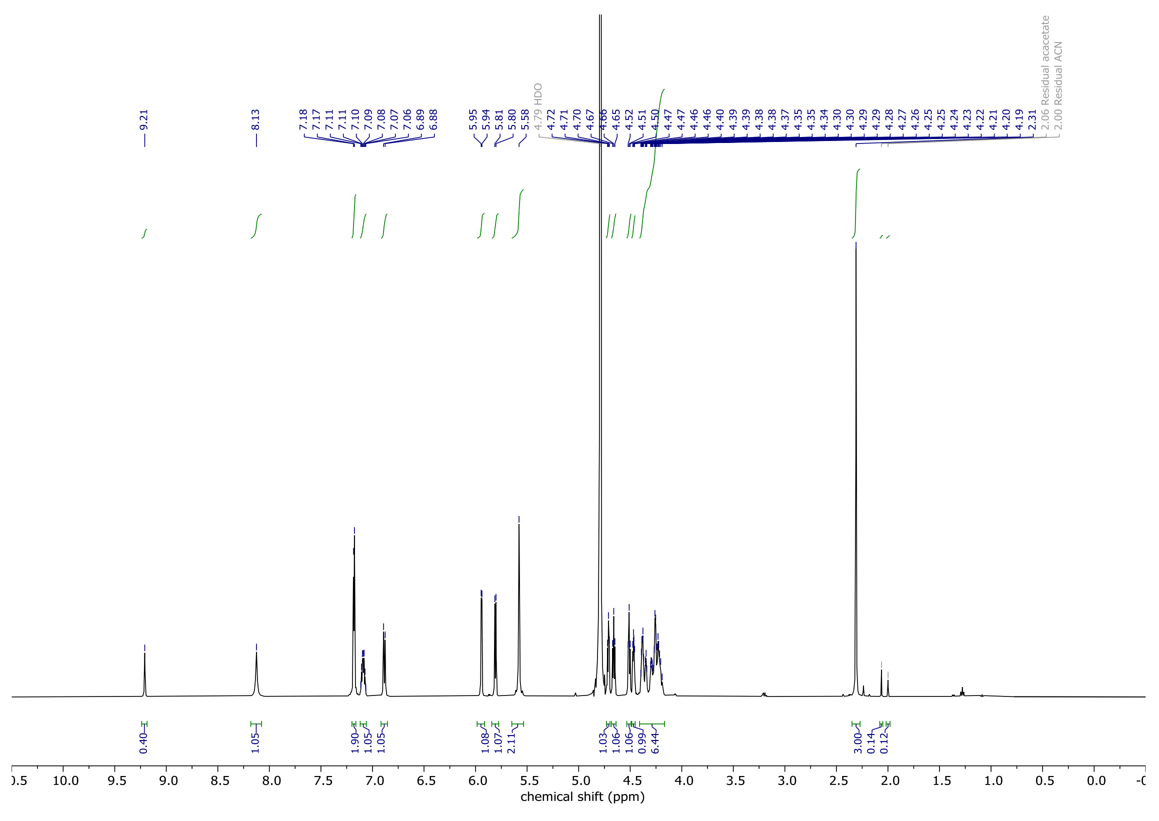

^{31}P NMR (202.49 MHz)

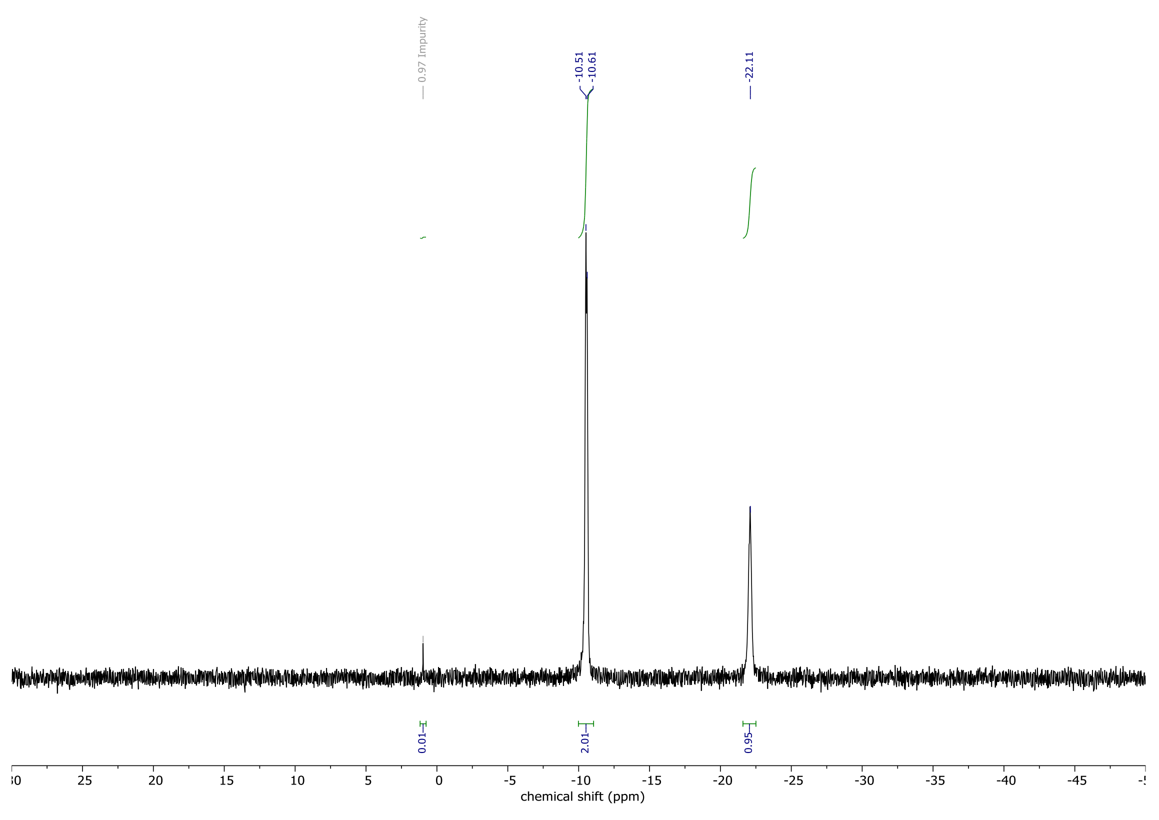

UV-VIS spectrum (recorded in 0.1M phosphate buffer pH	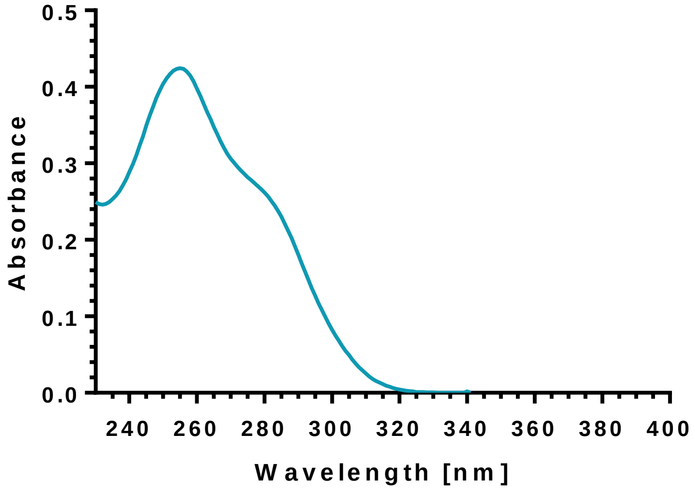

3-MeBn⁷GpppG (**1c**)	
Structure	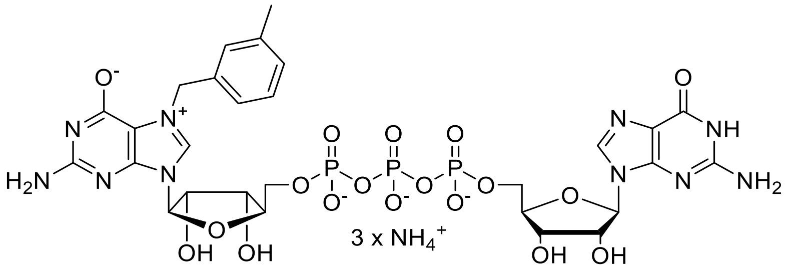
Reaction RP HPLC Profile	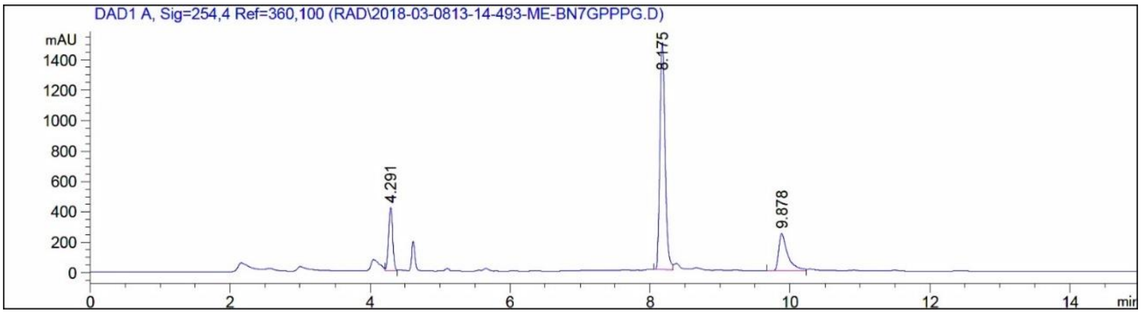
Purified Product RP HPLC Profile	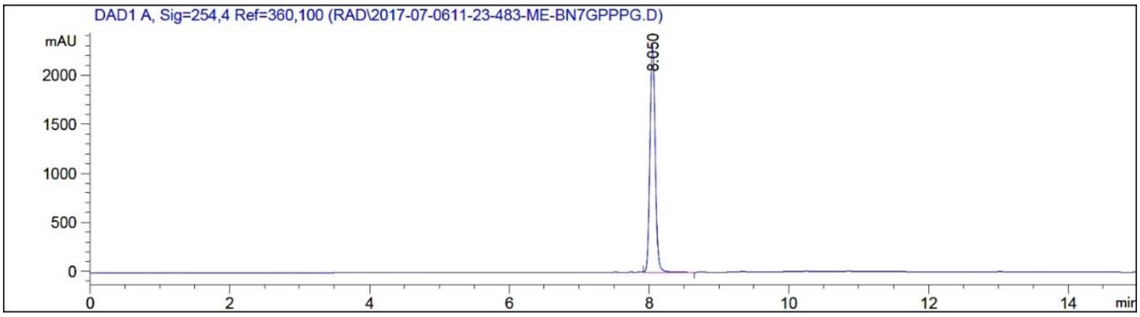
HRMS ES(-)	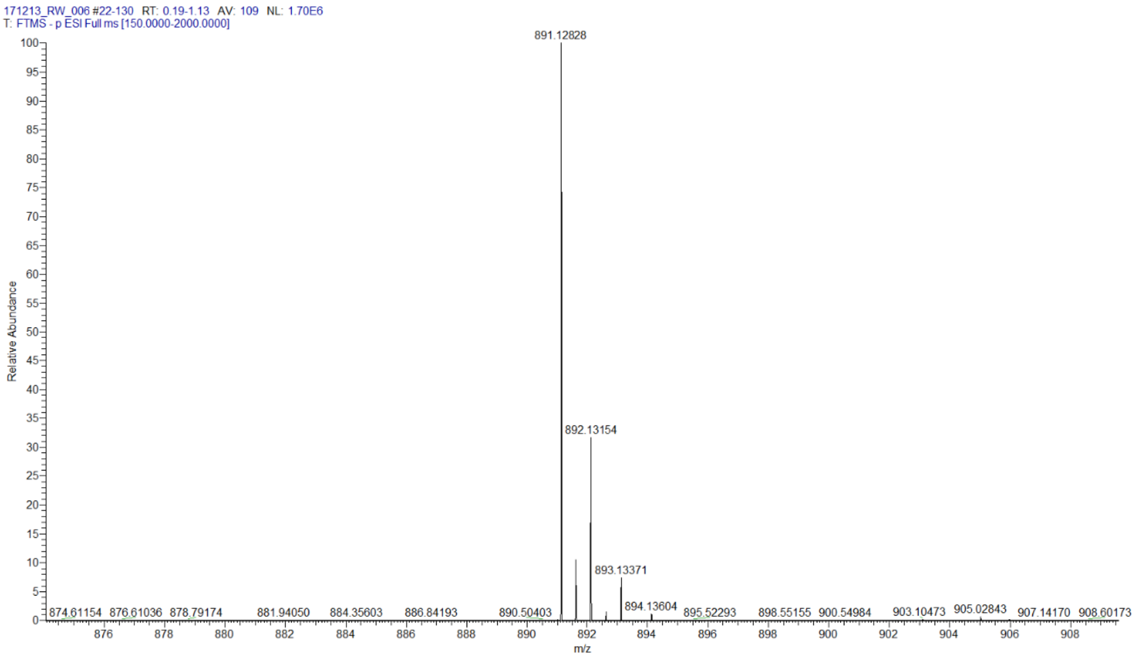

^{1}H NMR (500.24 MHz)	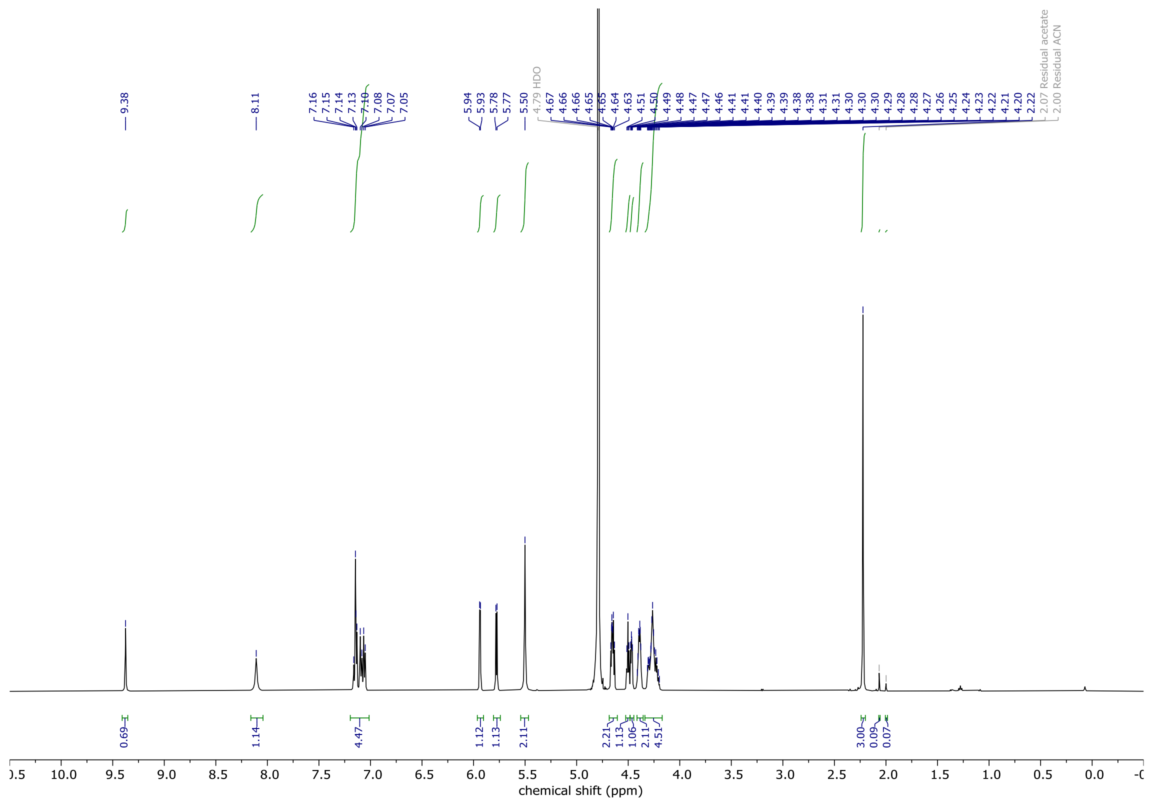
^{31}P NMR (202.49 MHz)	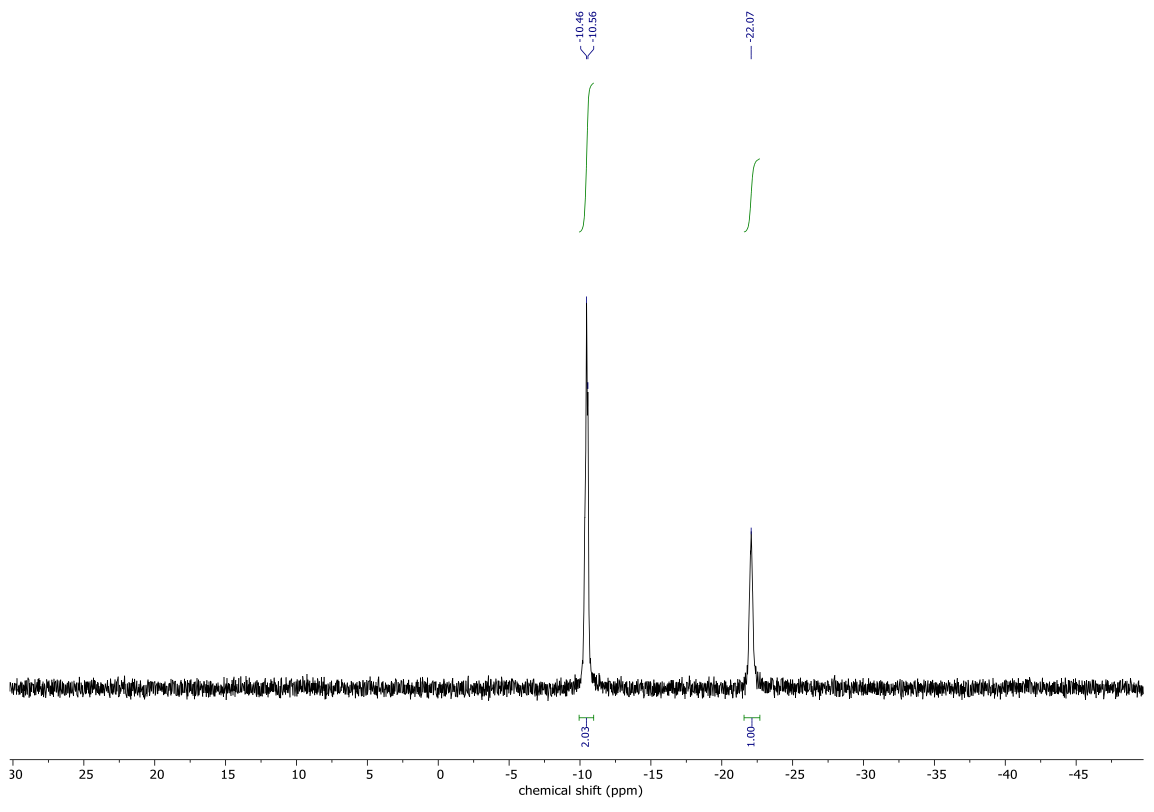

UV-VIS spectrum (recorded in 0.1M phosphate buffer pH 7.0)	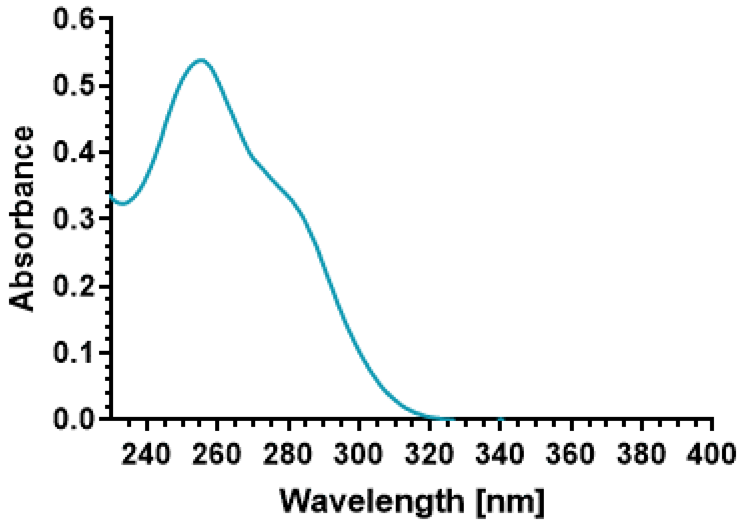

4-MeBn⁷GpppG (**1d**)

Structure

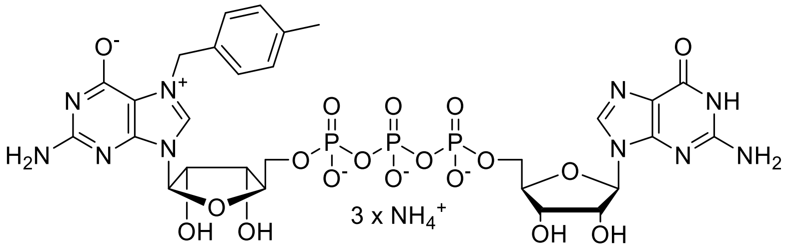

Reaction RP HPLC Profile

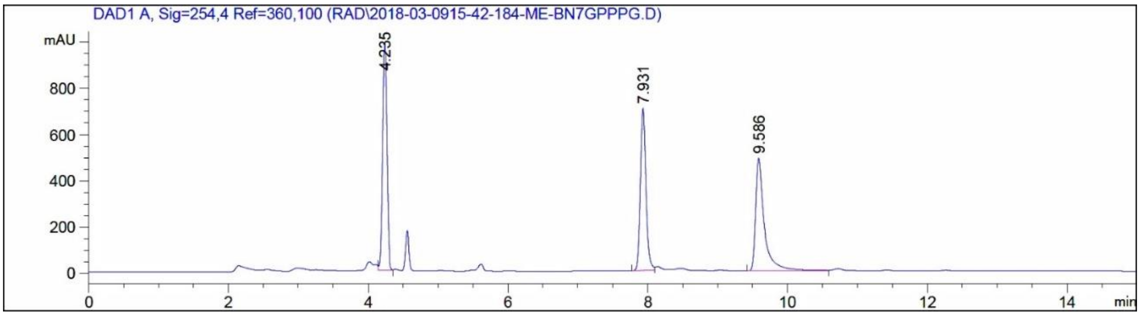

Purified Product RP HPLC Profile

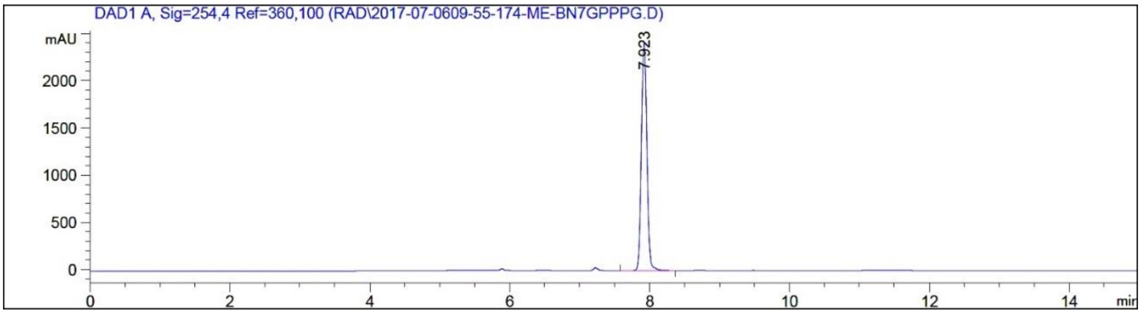

HRMS ES(-)

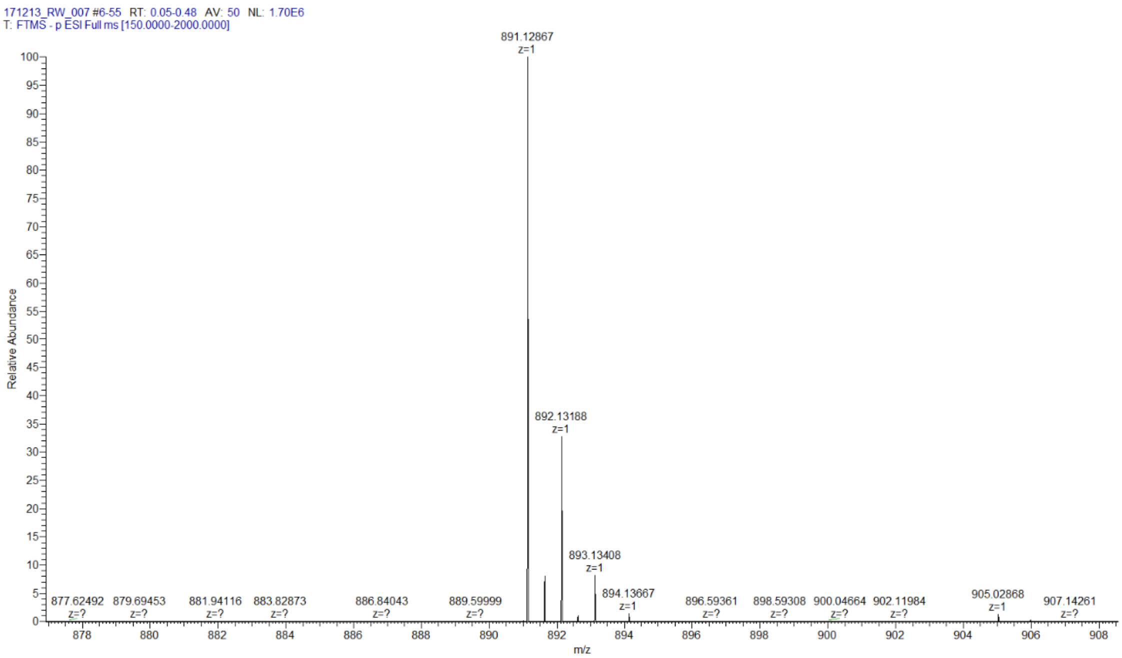

^{1}H NMR (500.24 MHz)	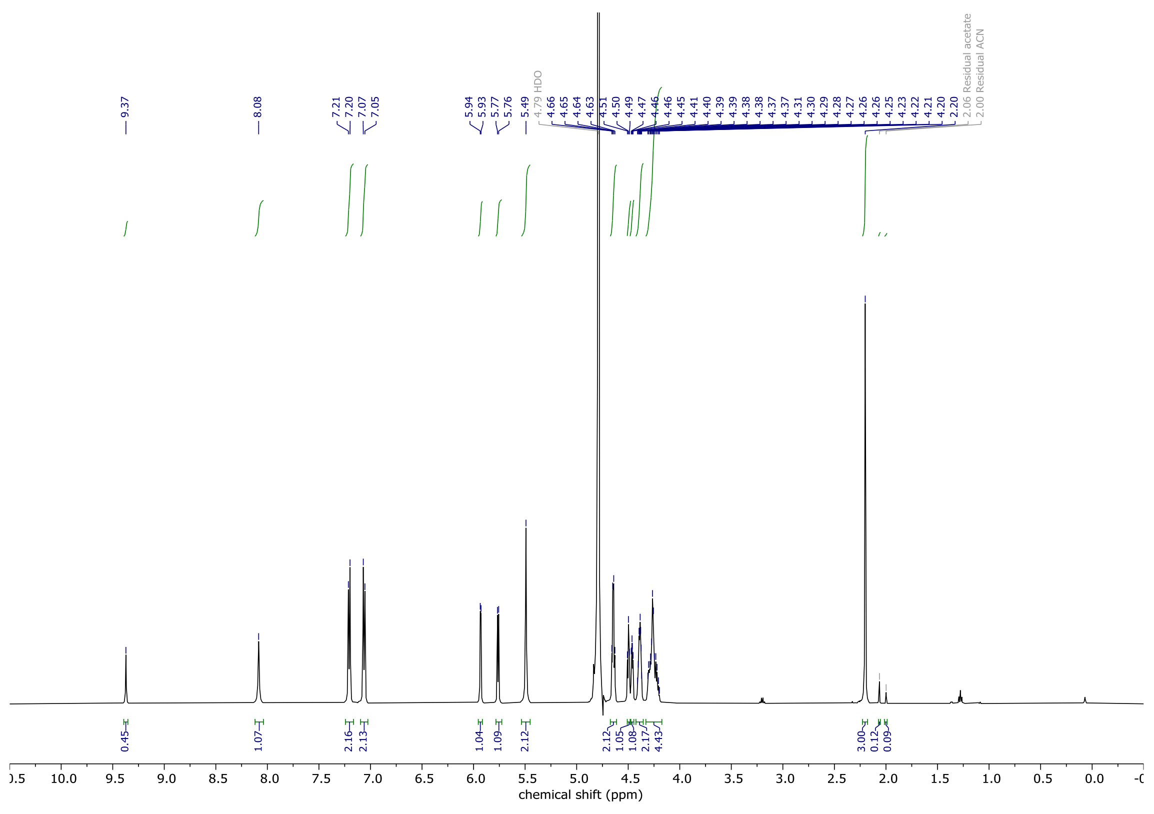
^{31}P NMR (202.49 MHz)	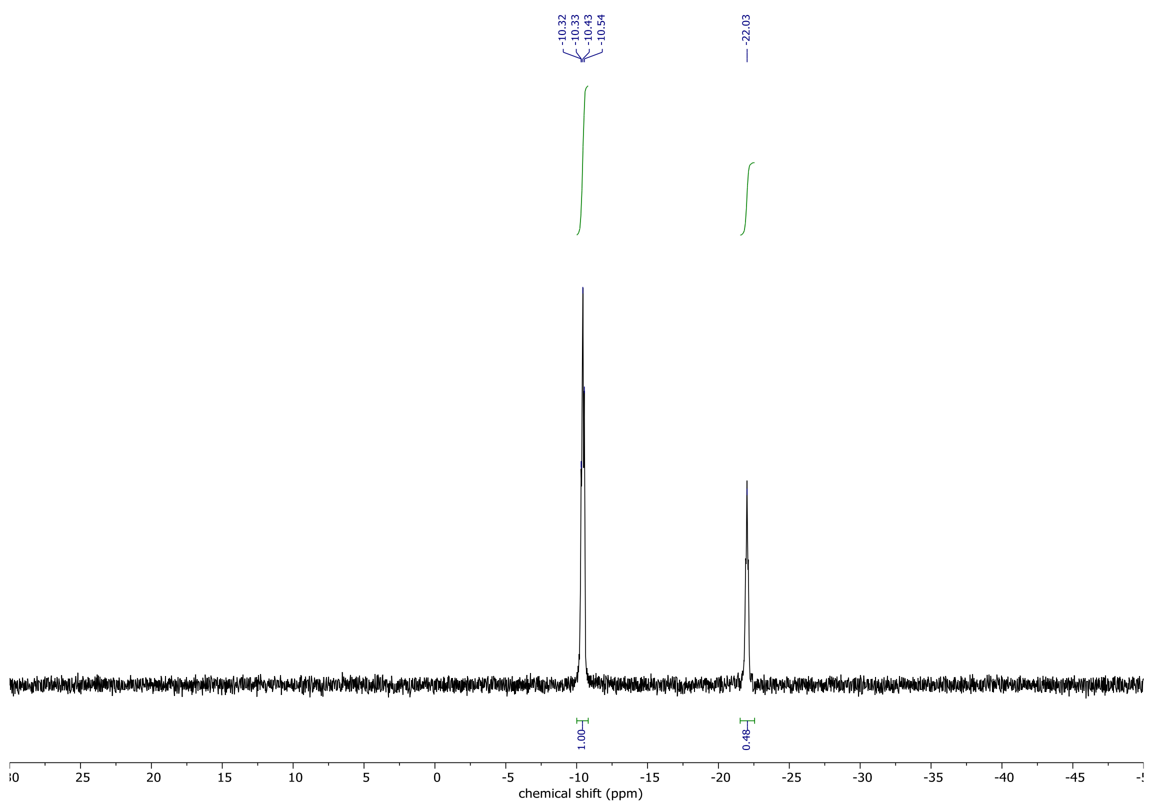

UV-VIS spectrum (recorded in 0.1M phosphate buffer pH 7.0)	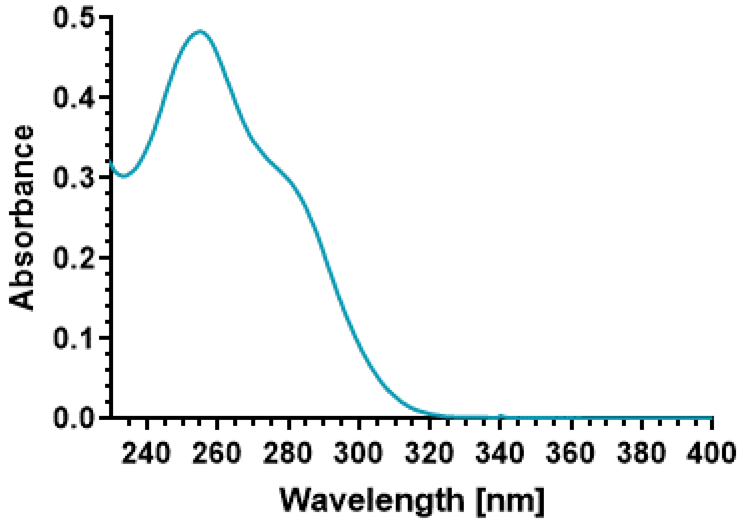

4-*i*PrBn^{7}GpppG (**1e**)	
Structure	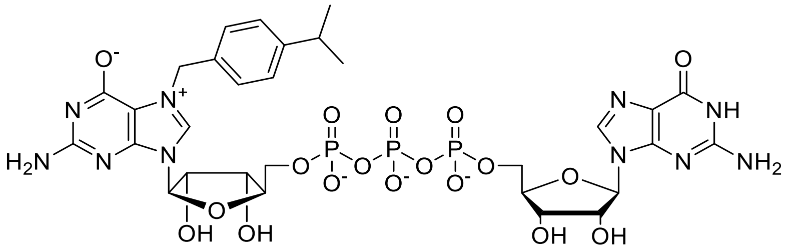
Reaction RP HPLC Profile	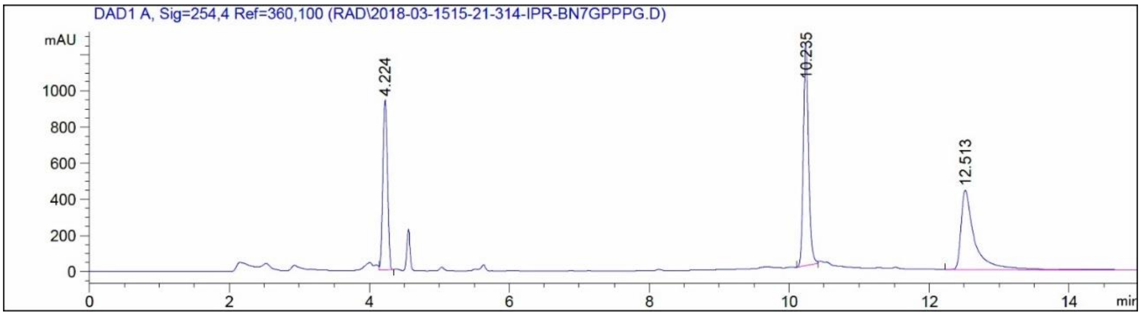
Purified Product RP HPLC Profile	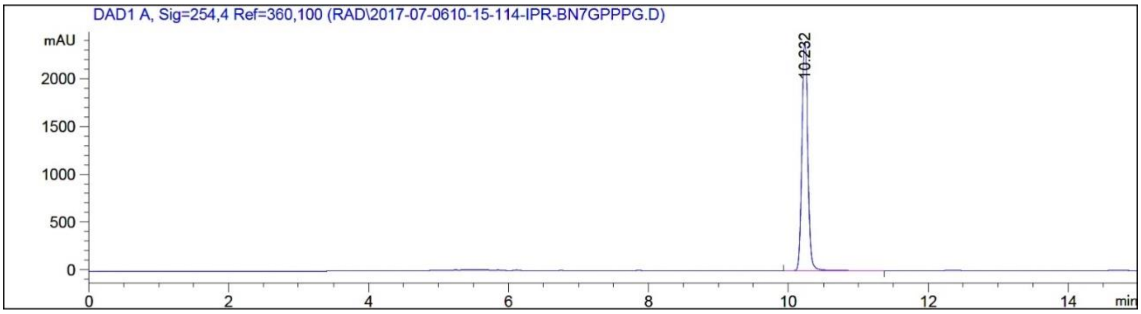
HRMS ES(-)	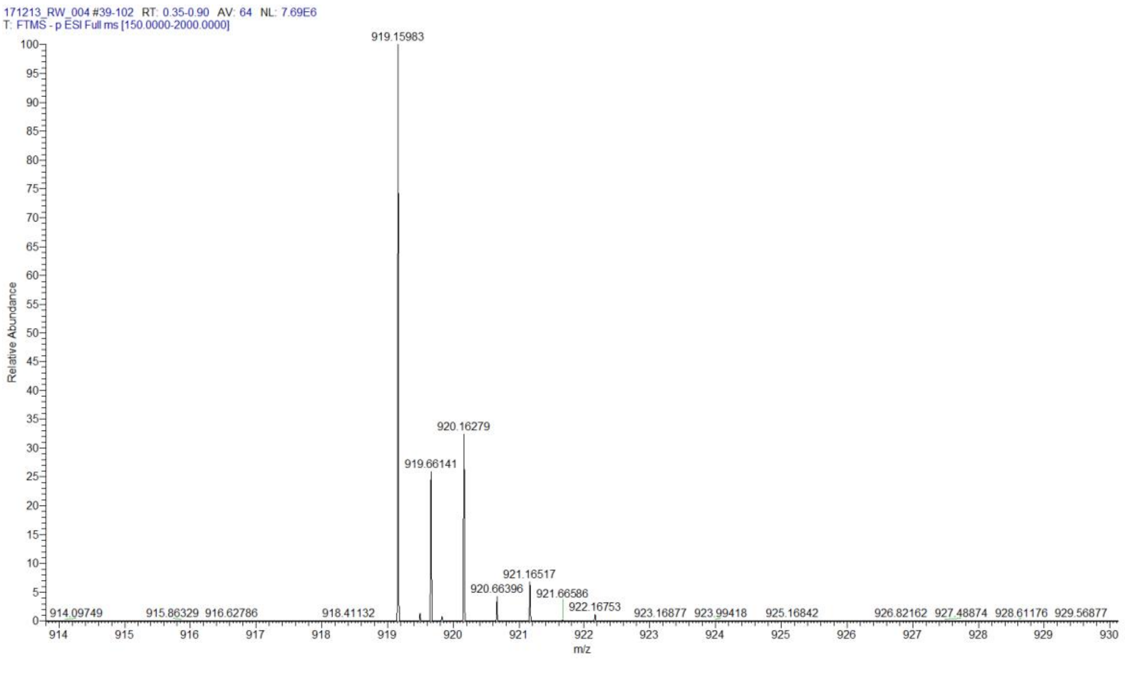

^{1}H NMR (500.24 MHz)	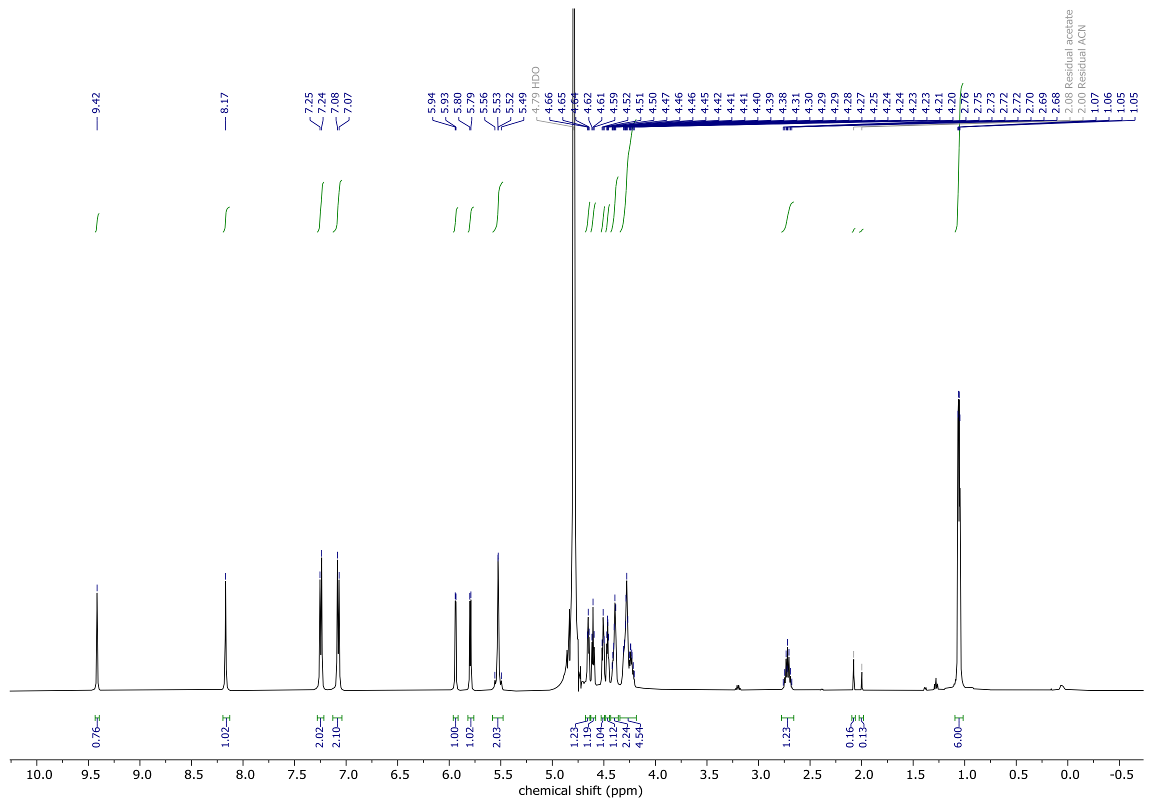
^{31}P NMR (202.49 MHz)	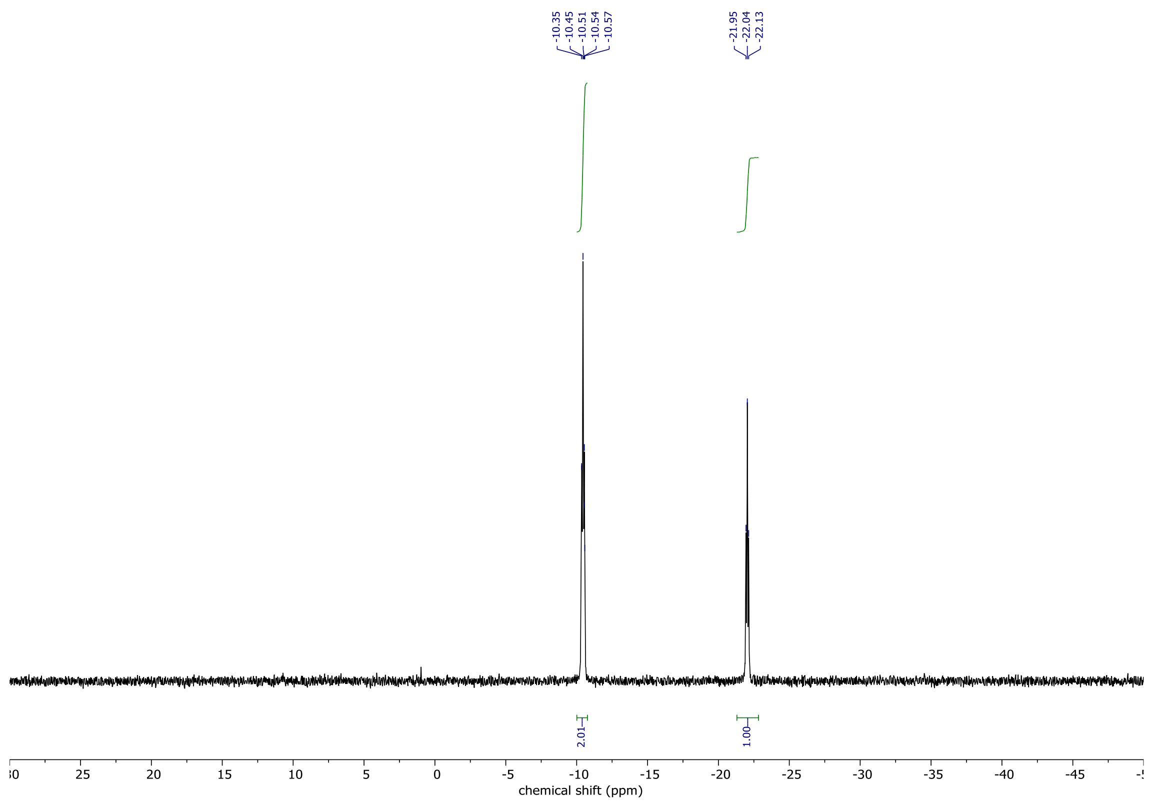

UV-VIS spectrum (recorded in 0.1M phosphate buffer pH 7.0)	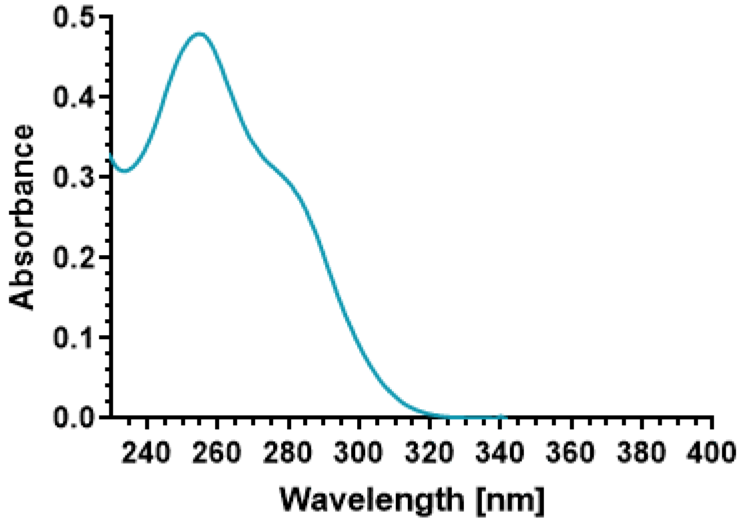

3,5-di-MeBn^{7}GpppG (**1f**)

Structure

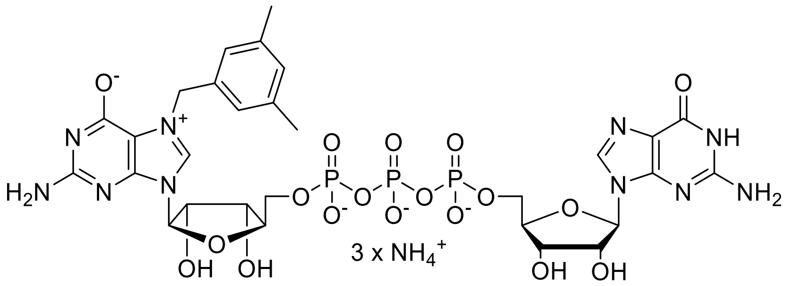

Reaction RP HPLC Profile

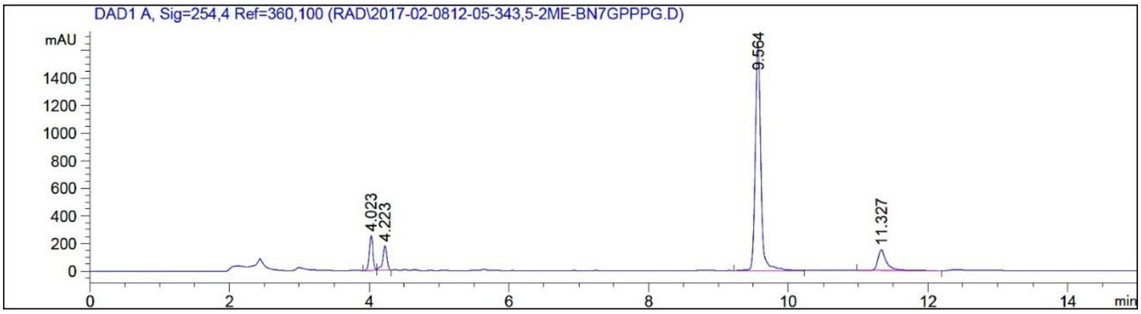

Purified Product RP HPLC Profile

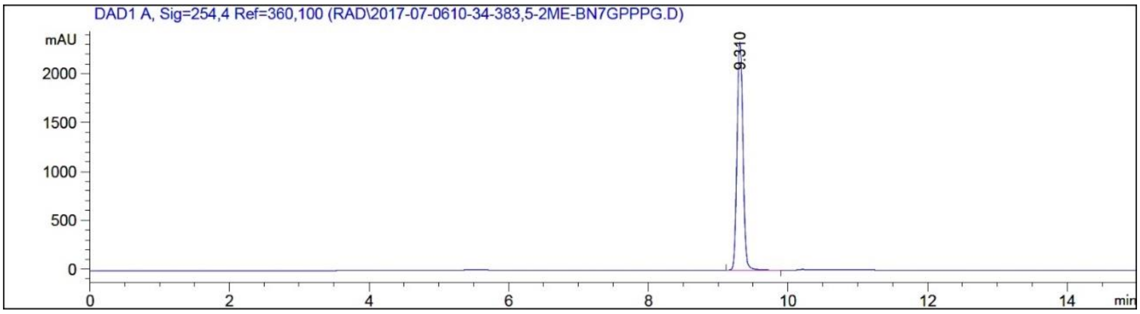

HRMS ES(-)

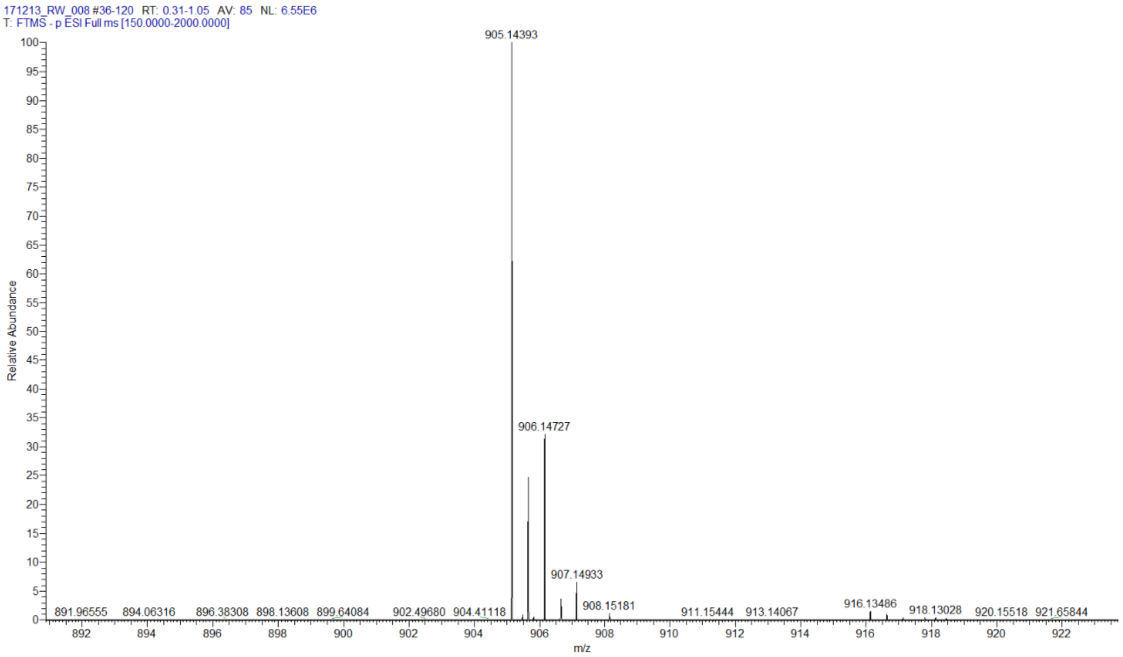

^1H NMR (500.24 MHz)	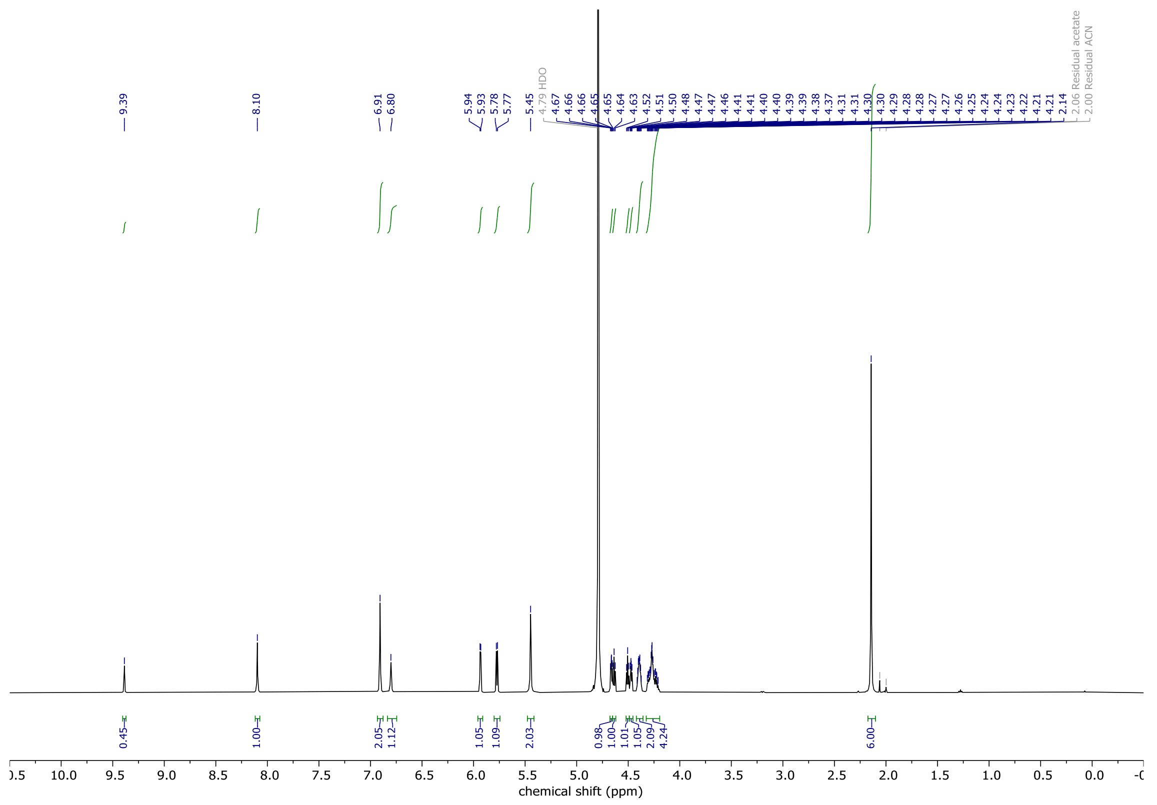
^{31}P NMR (202.49 MHz)	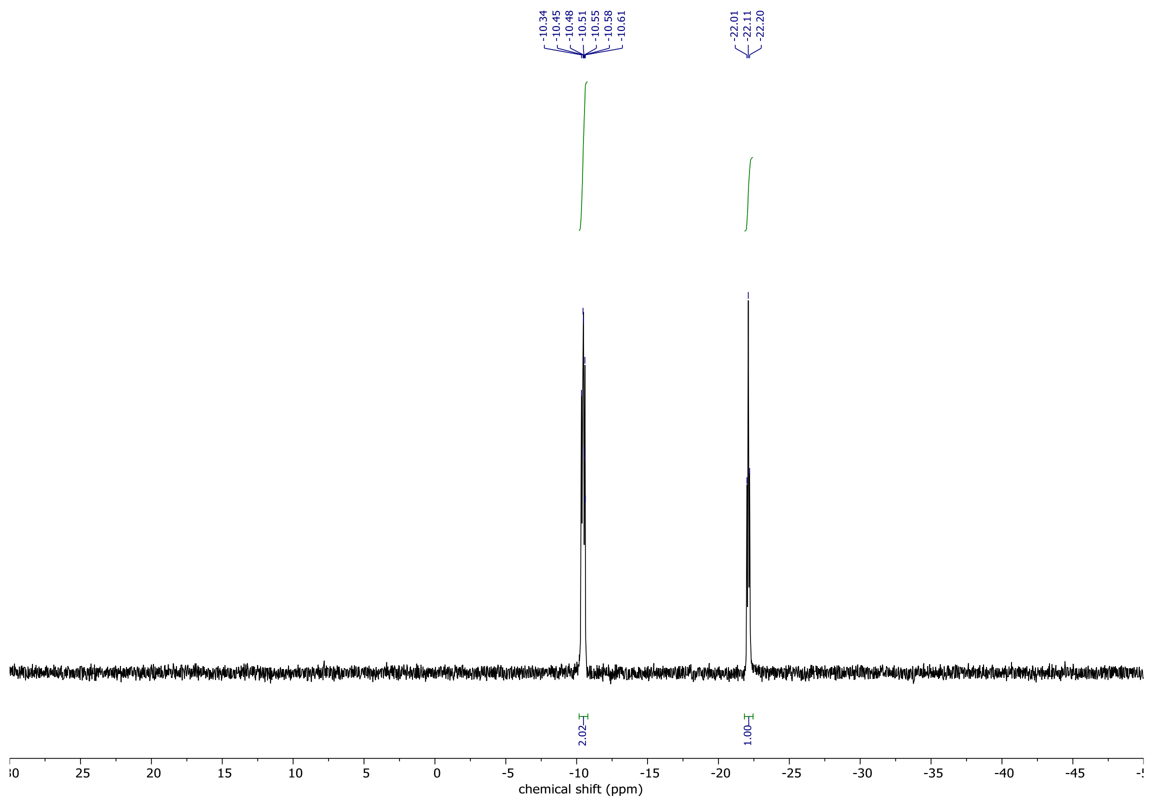

UV-VIS spectrum (recorded in 0.1M phosphate buffer pH	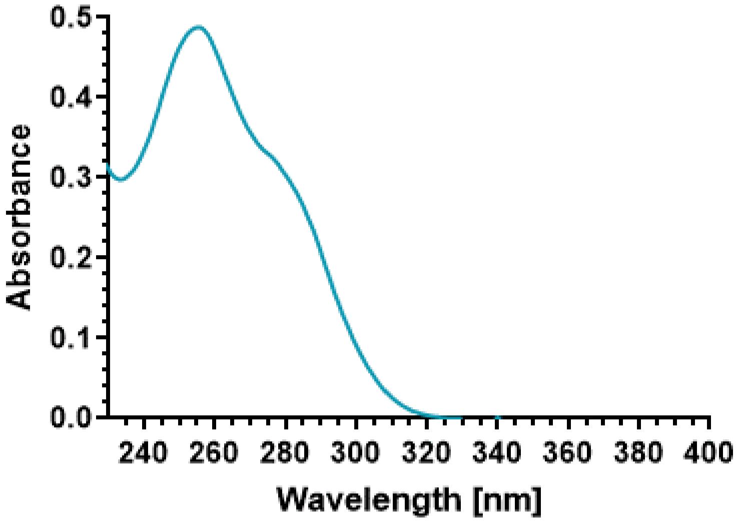

<table>
<tr><th colspan="2">4-F-Bn⁷GpppG (1g)</th></tr>
<tr><td>Structure</td><td>
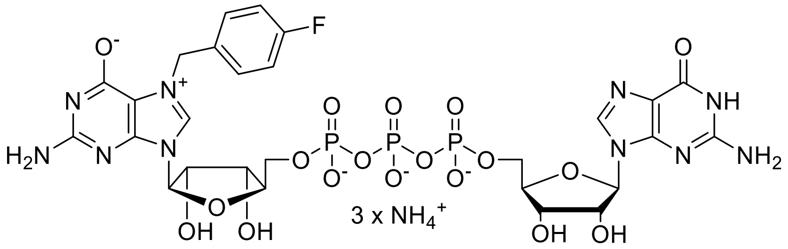

</td></tr>
<tr><td>Reaction RP HPLC Profile</td><td>
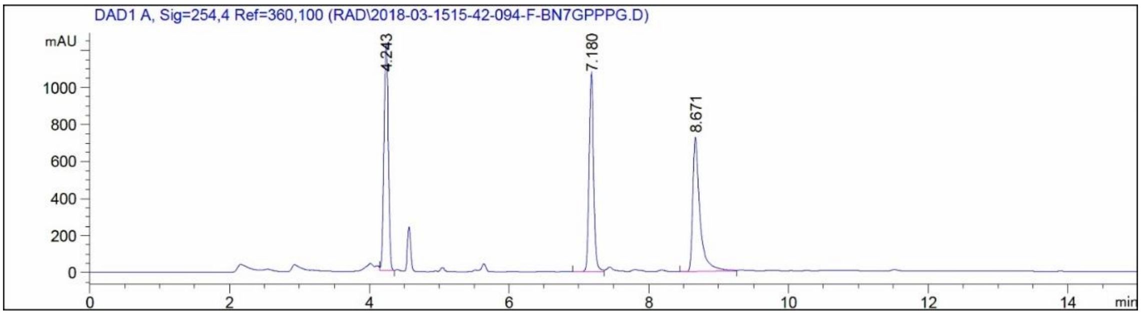

</td></tr>
<tr><td>Purified Product RP HPLC Profile</td><td>
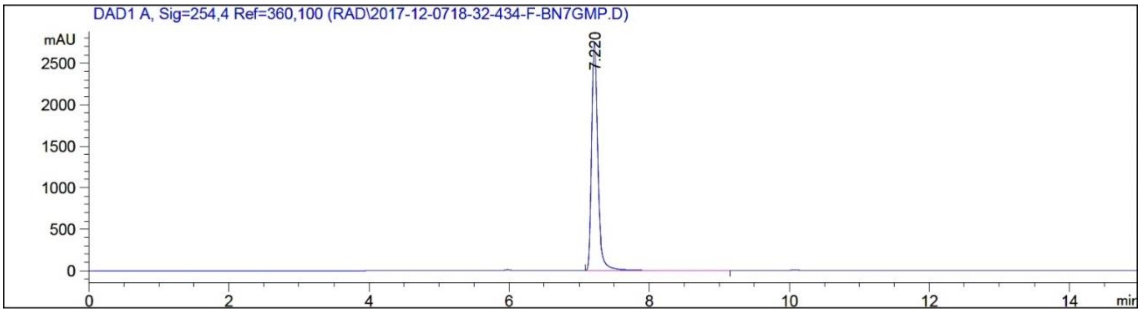

</td></tr>
<tr><td>HRMS ES(-)</td><td>
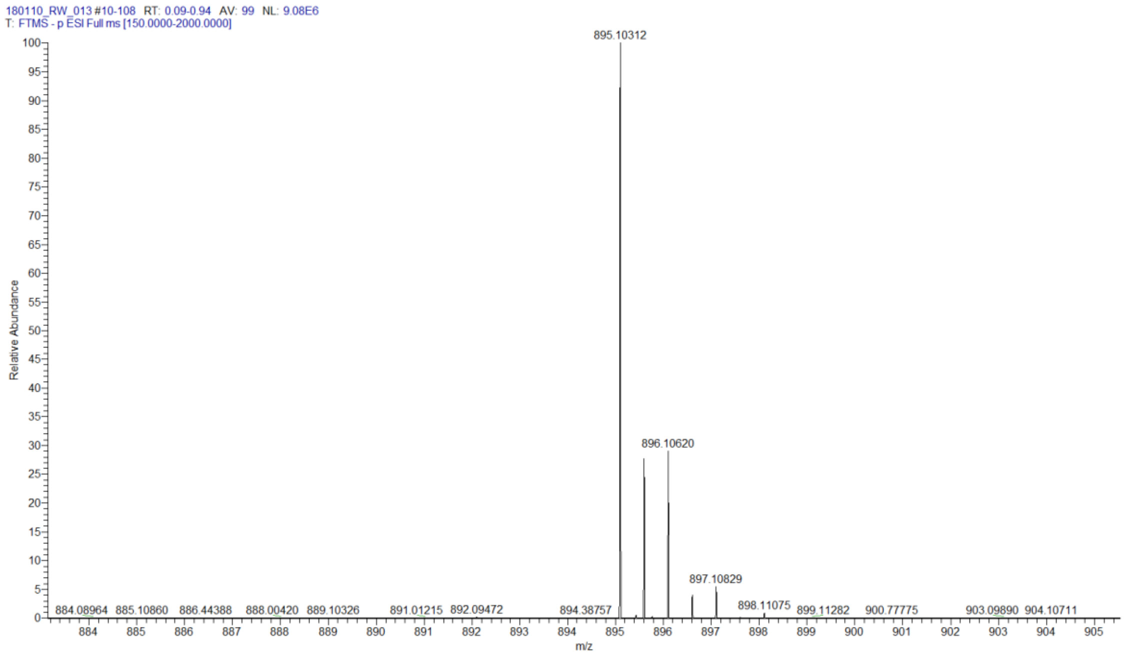

</td></tr>
</table>

^{1}H NMR (500.24 MHz)

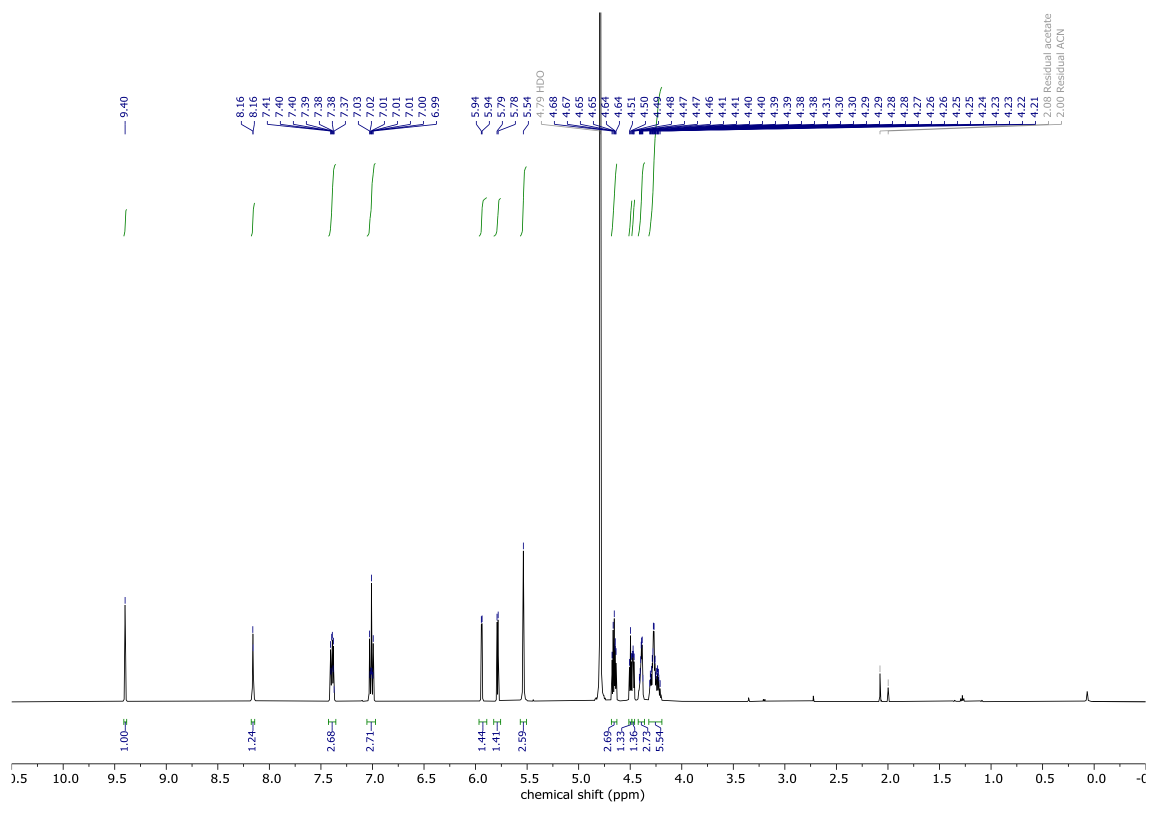

^{31}P NMR (202.49 MHz)

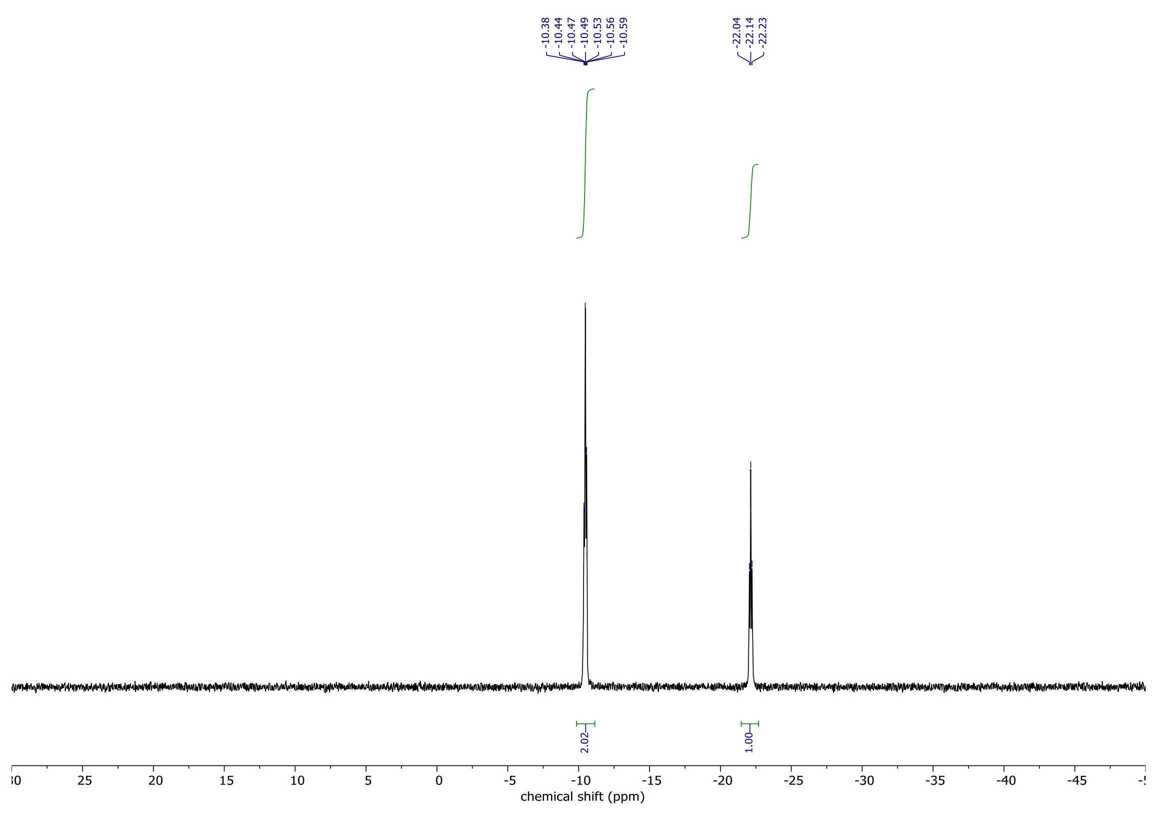

^{19}F NMR (470.65 MHz)	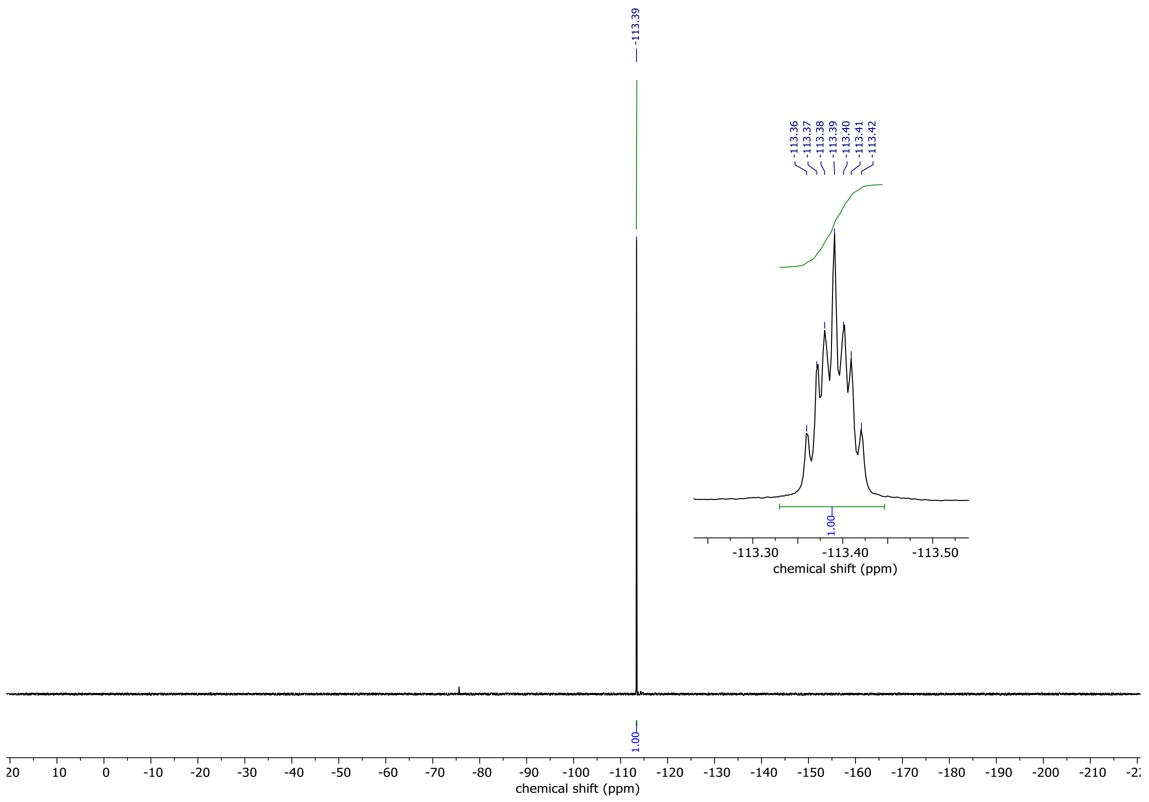
^{19}F {1H} NMR (470.65 MHz)	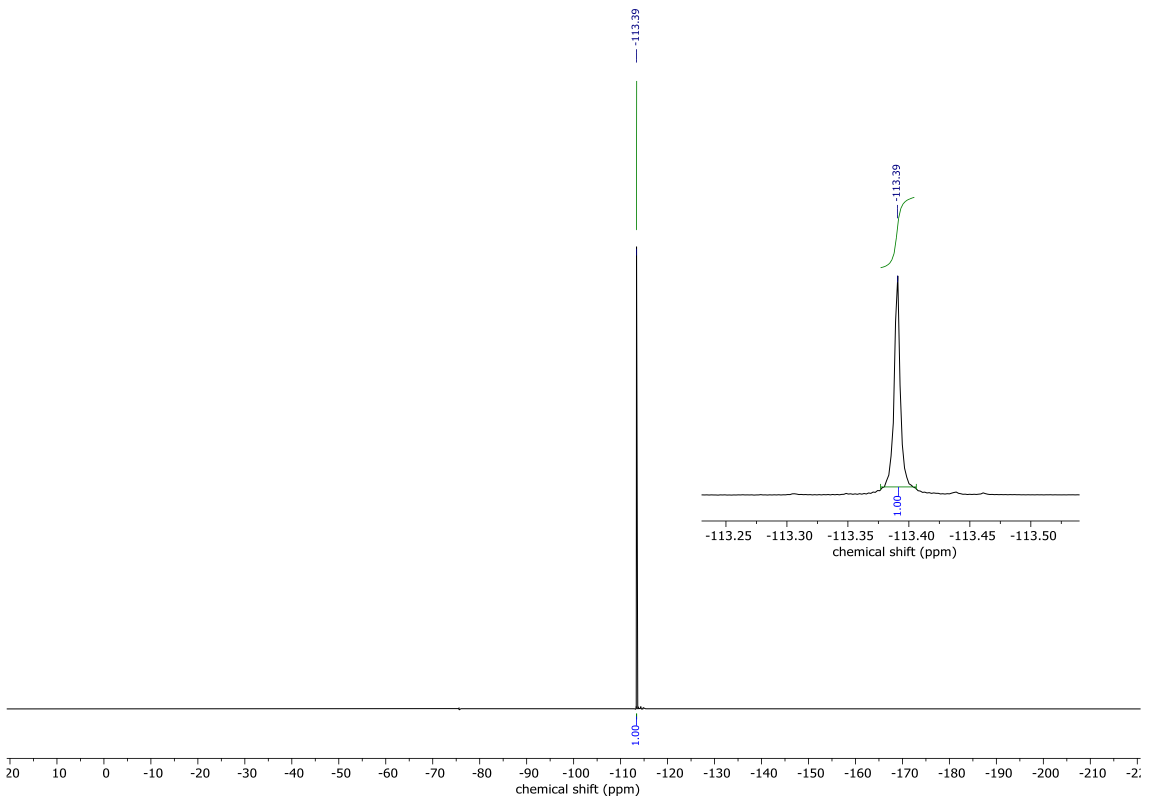

UV-VIS spectrum (recorded in 0.1M phosphate buffer pH 7.0)	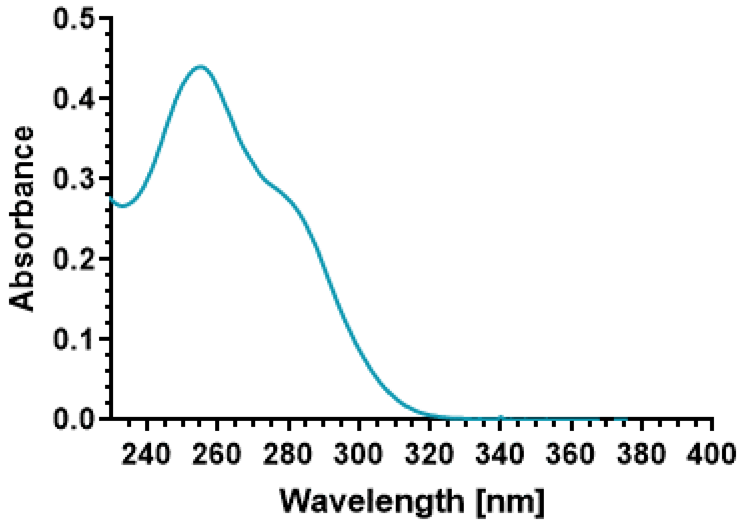

4-Cl-Bn^{7}GpppG (**1h**)	
Structure	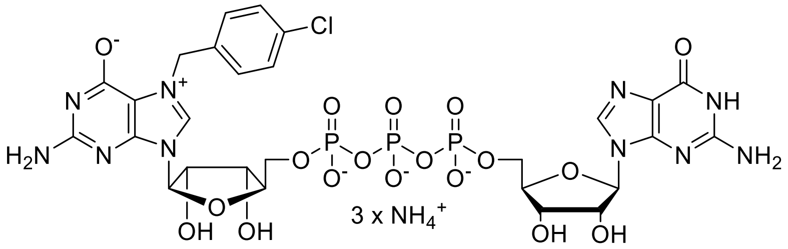
Reaction RP HPLC Profile	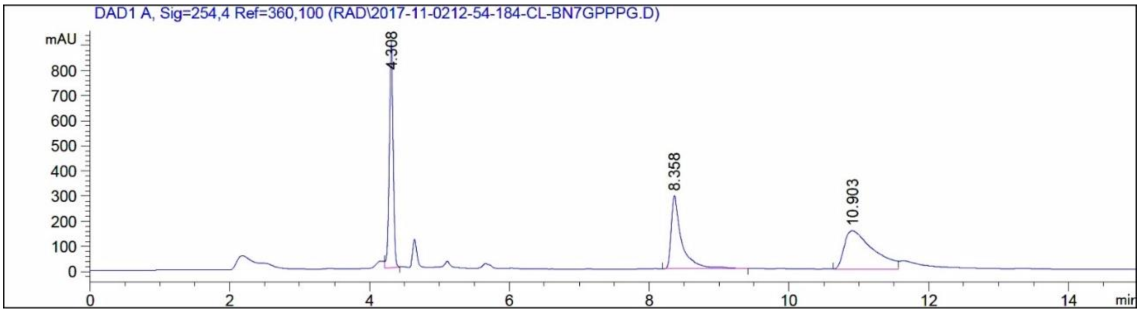
Purified Product RP HPLC Profile	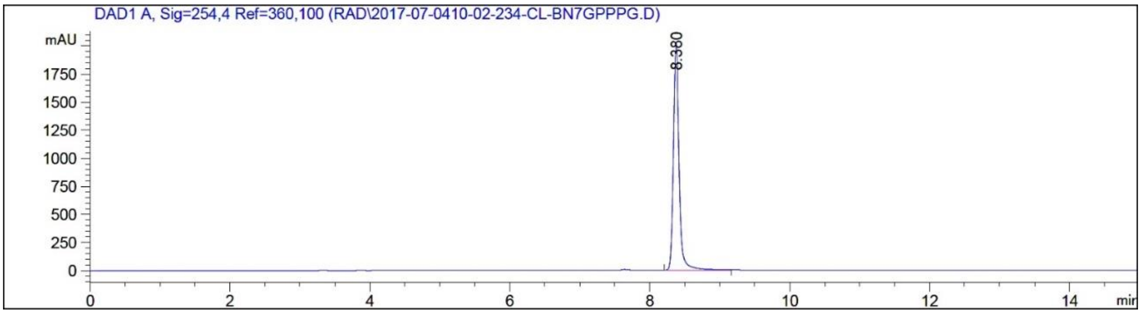
HRMS ES(-)	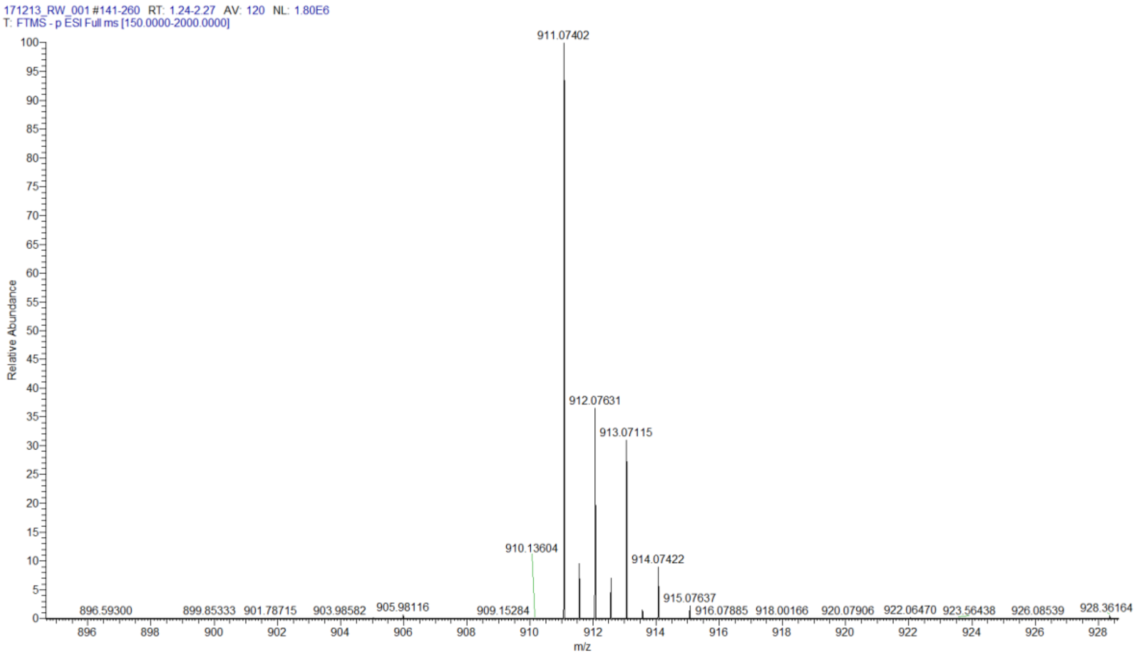

^{1}H NMR (500.24 MHz)	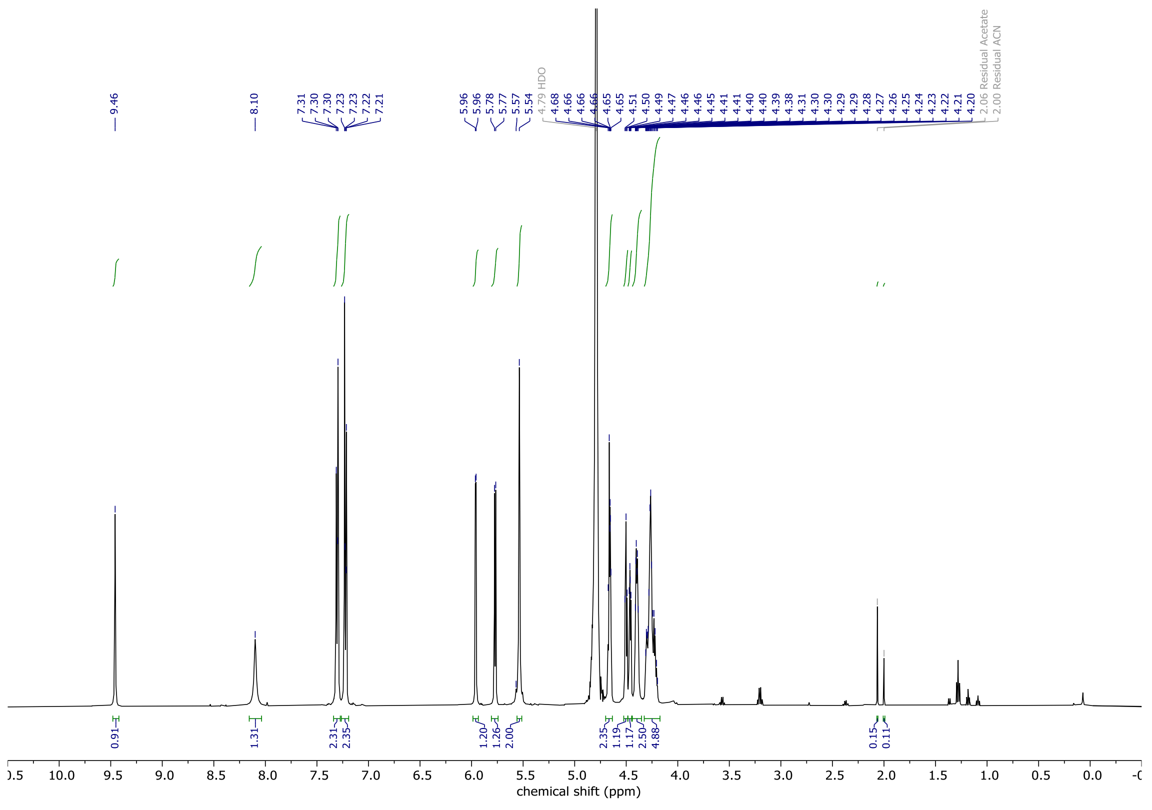
^{31}P NMR (202.49 MHz)	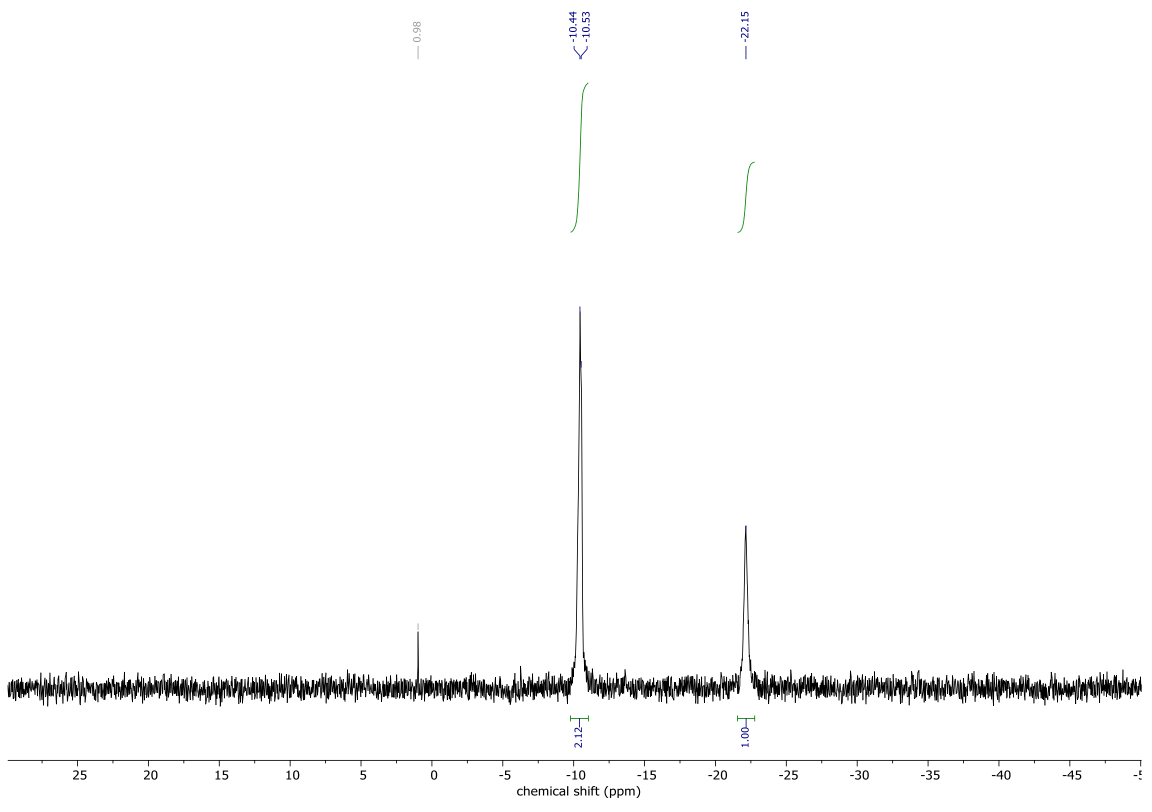

UV-VIS spectrum (recorded in 0.1M phosphate buffer pH	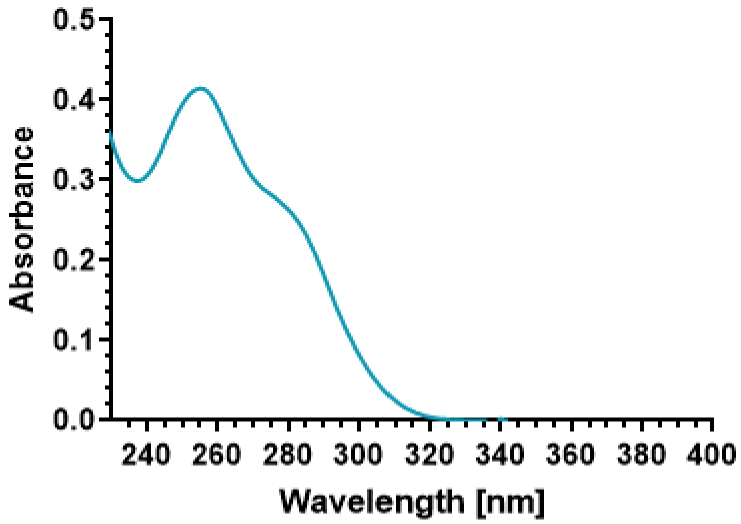

4-Br-Bn^{7}GpppG (**1i**)

Structure

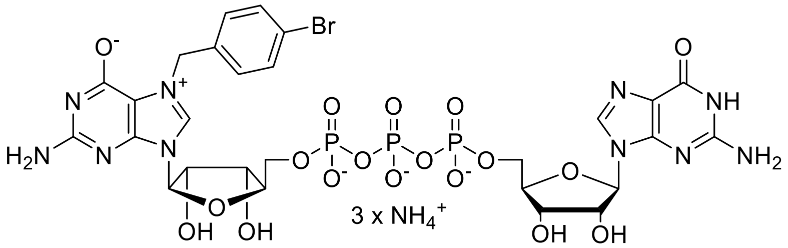

Reaction RP HPLC Profile

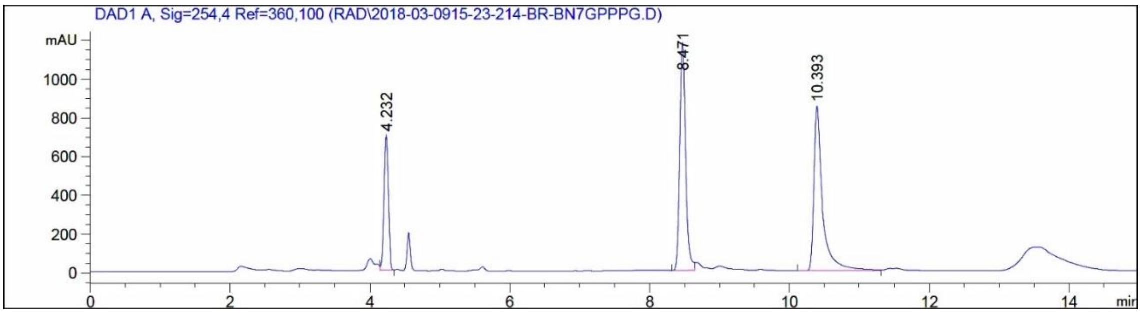

Purified Product RP HPLC Profile

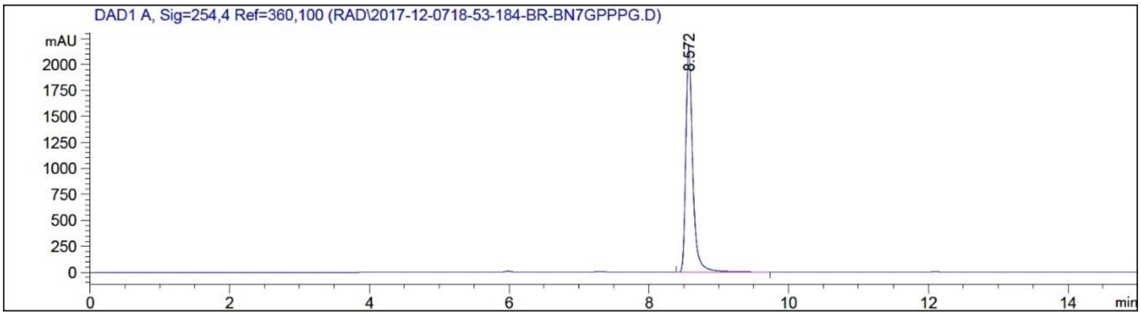

HRMS ES(-)

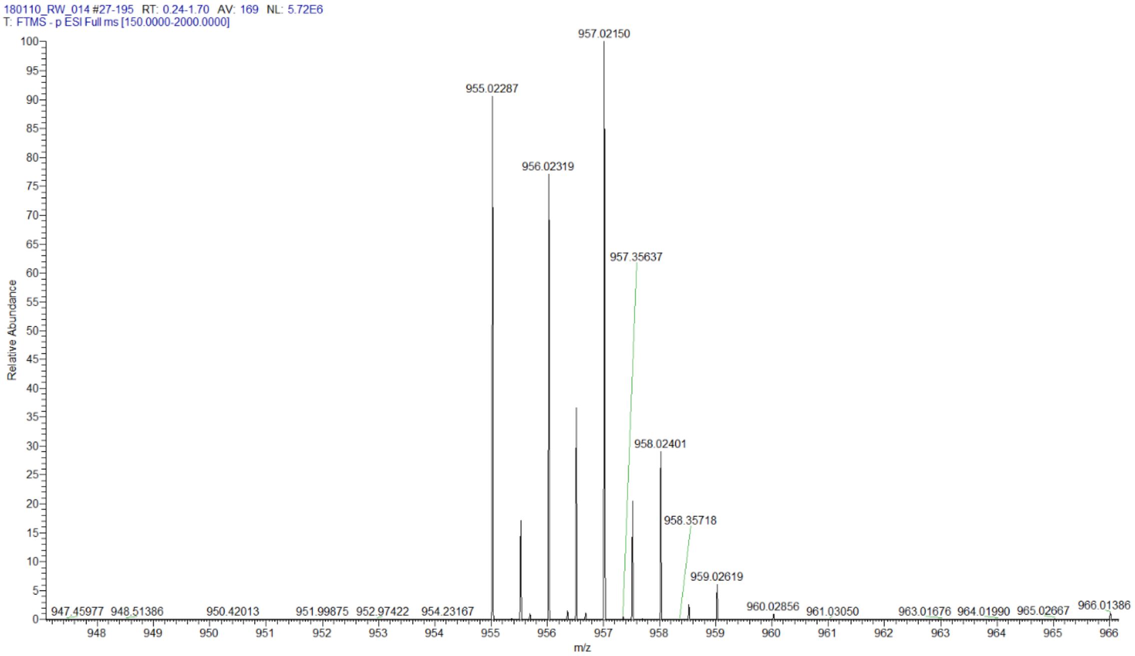

^{1}H NMR (500.24 MHz)	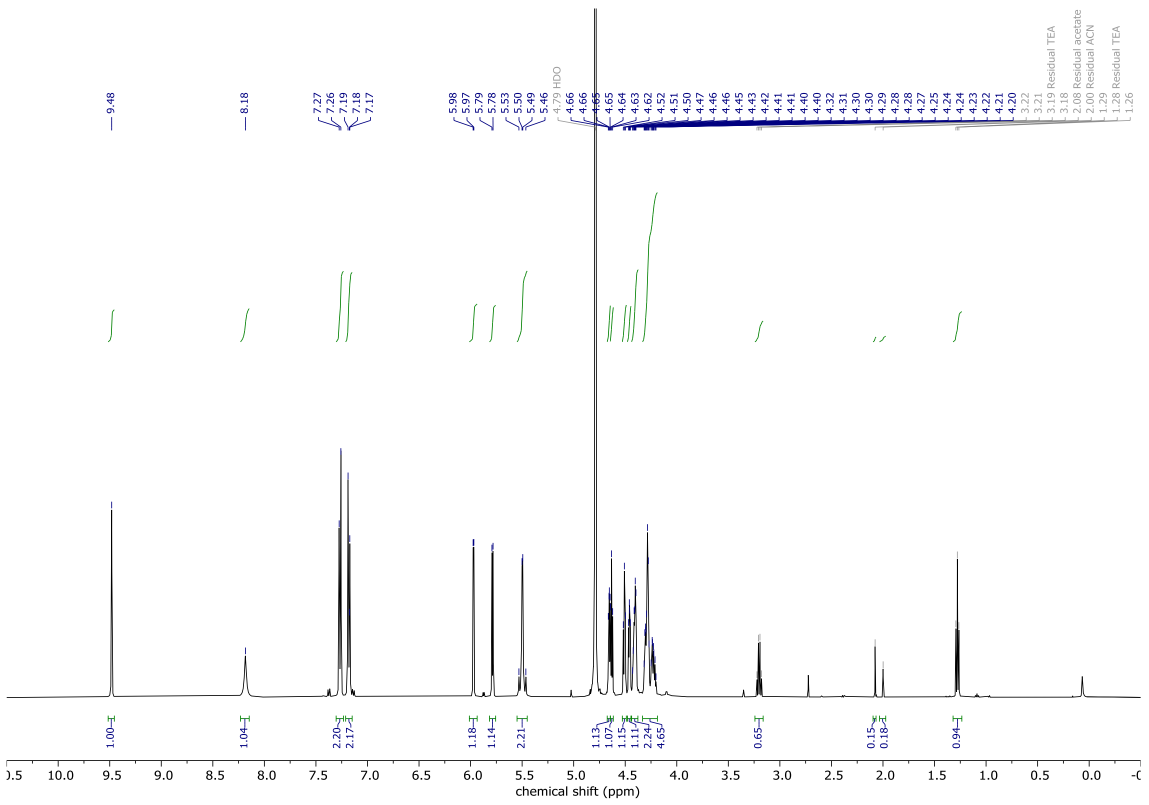
^{31}P NMR (202.49 MHz)	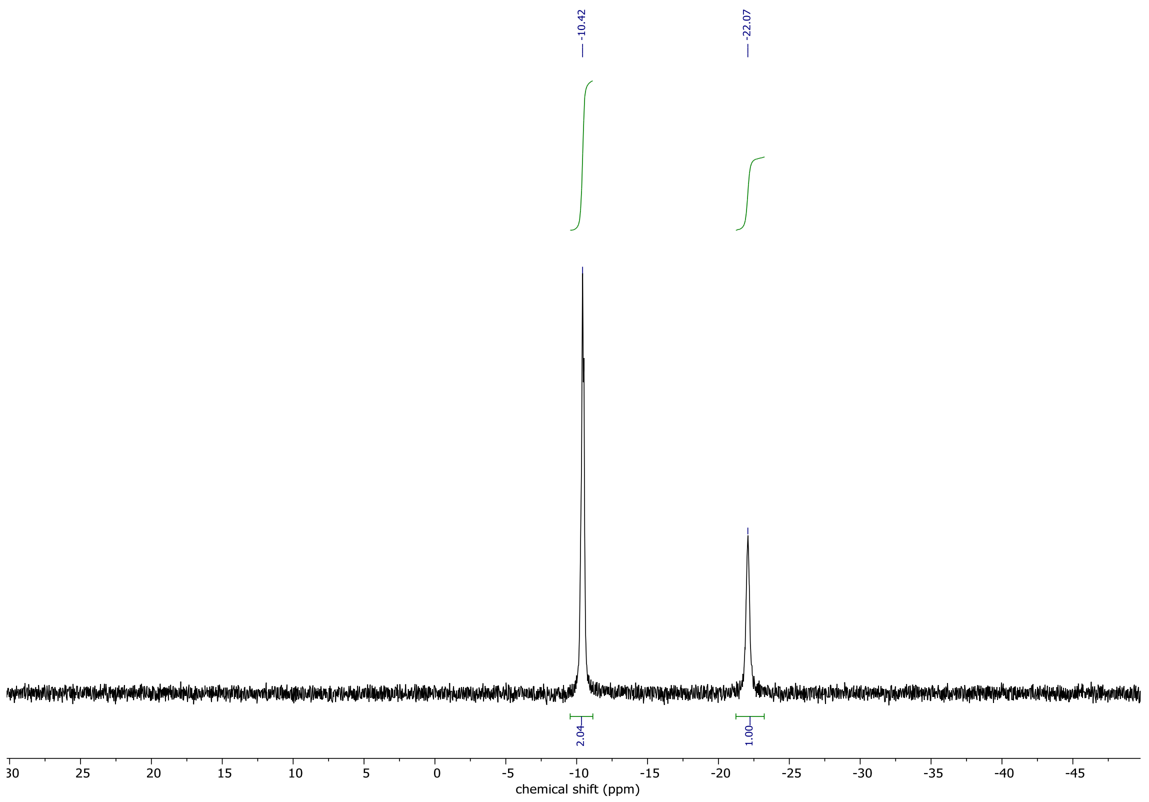

UV-VIS spectrum (recorded in 0.1M phosphate buffer pH 7.0)	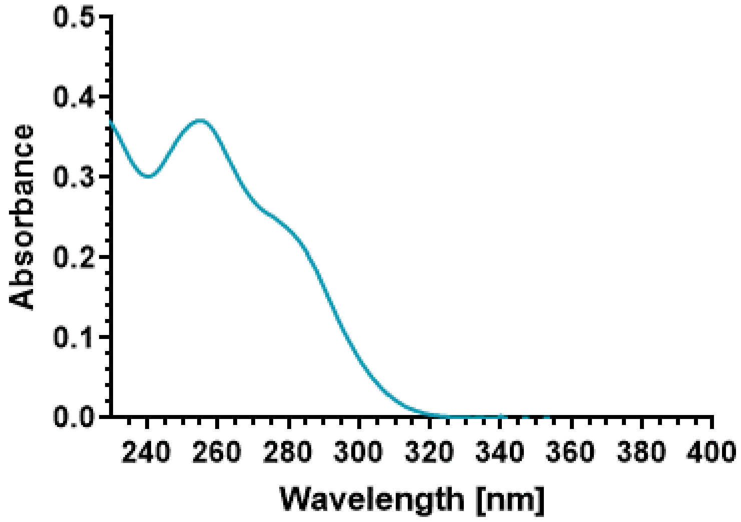

2,4-di-F-Bn⁷GpppG (**1j**)	
Structure	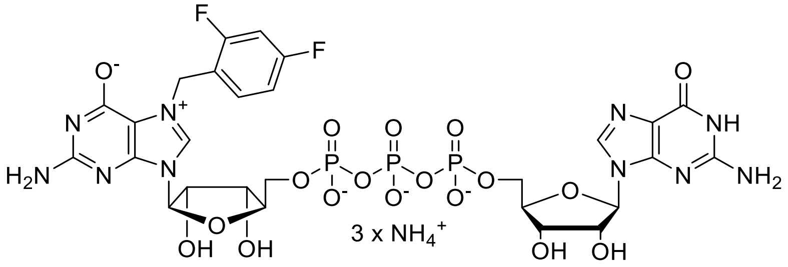
Reaction RP HPLC Profile	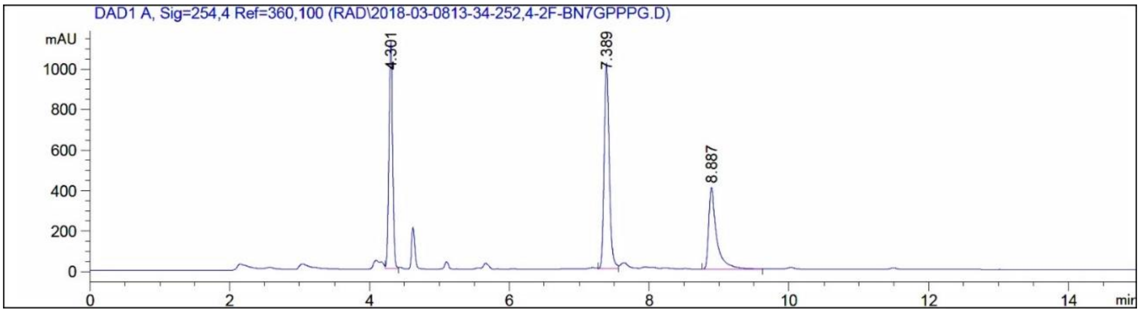
Purified Product RP HPLC Profile	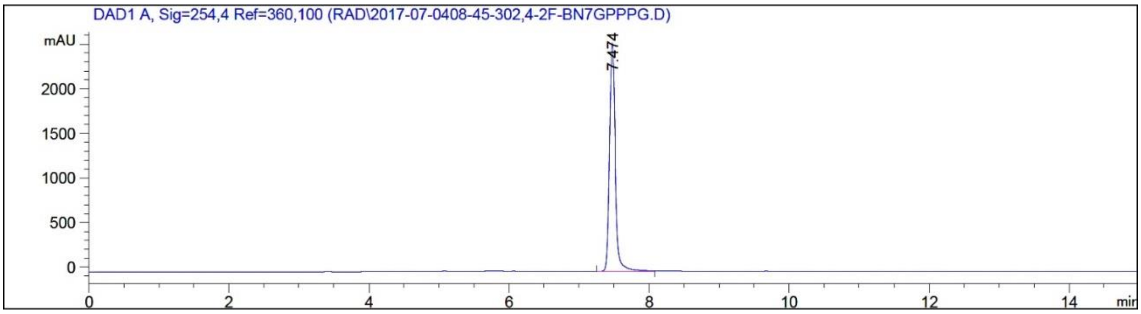
HRMS ES(-)	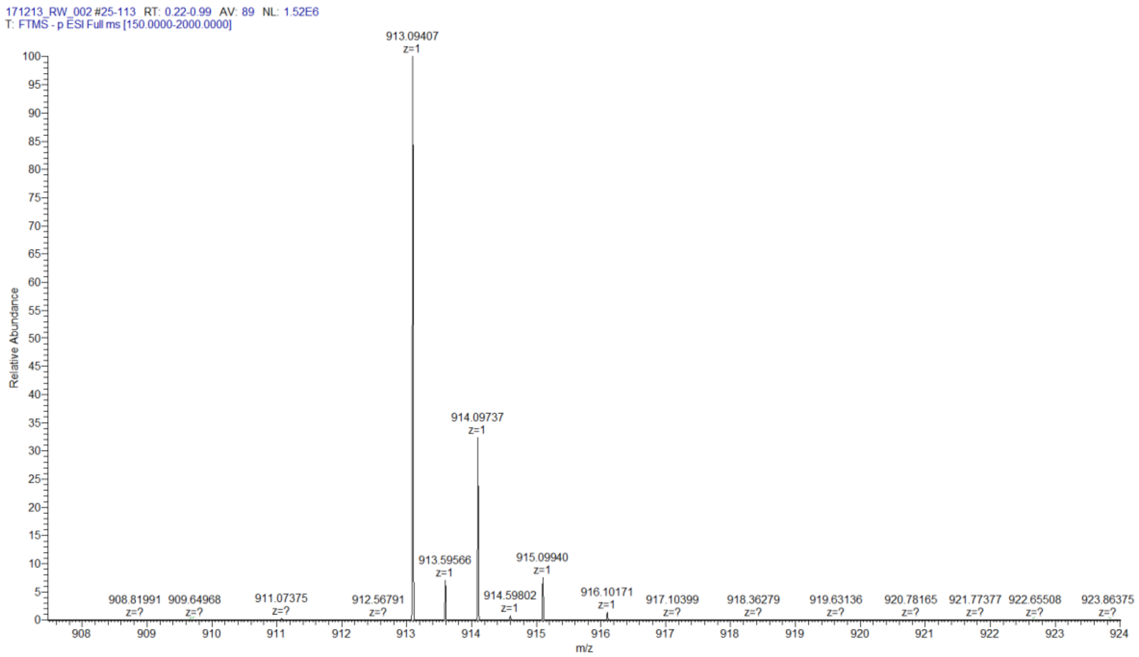

^{1}H NMR (500.24 MHz)	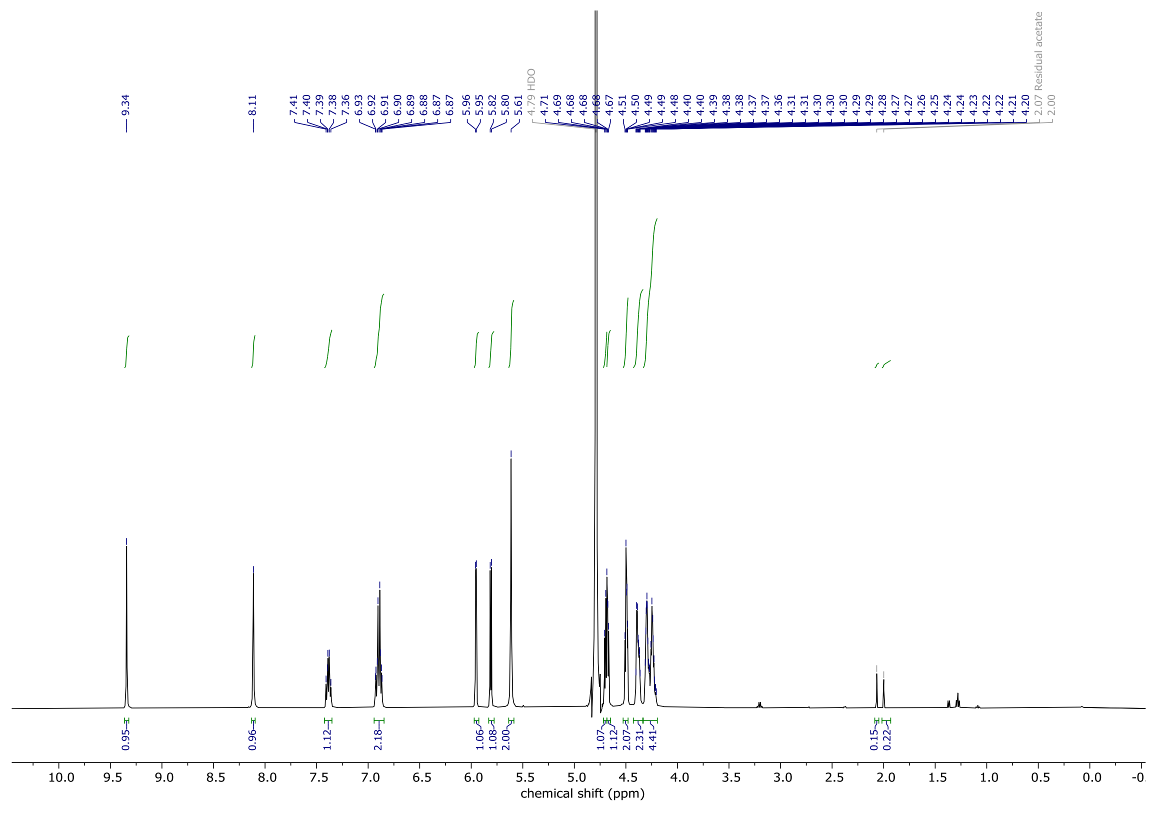
^{31}P NMR (202.49 MHz)	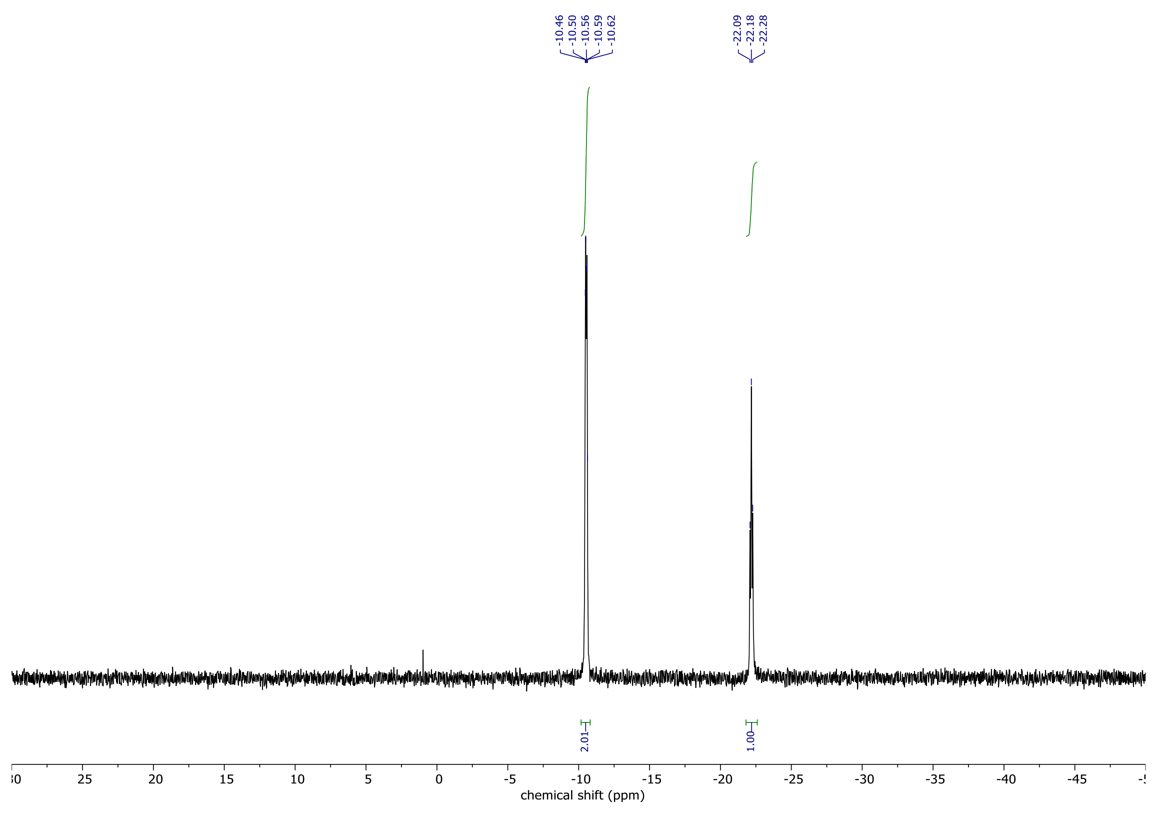

^{19}F NMR (470.65 MHz)	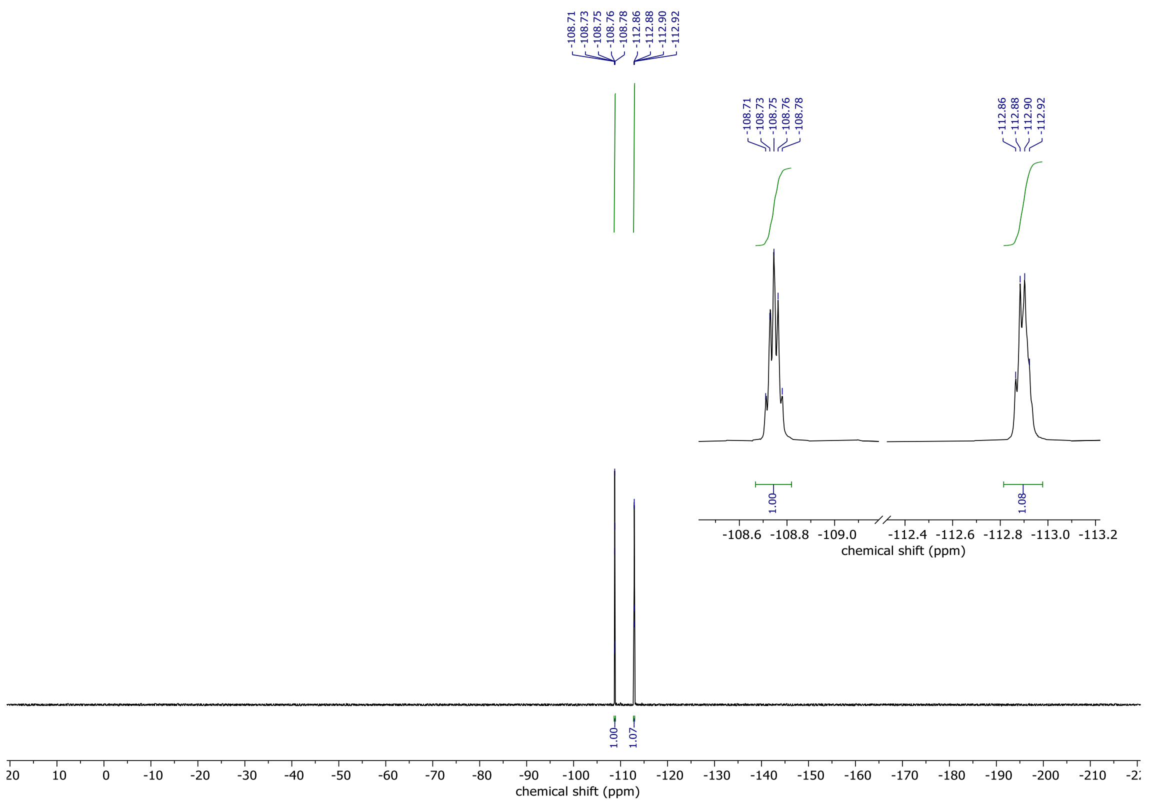
^{19}F {1H} NMR (470.65 MHz)	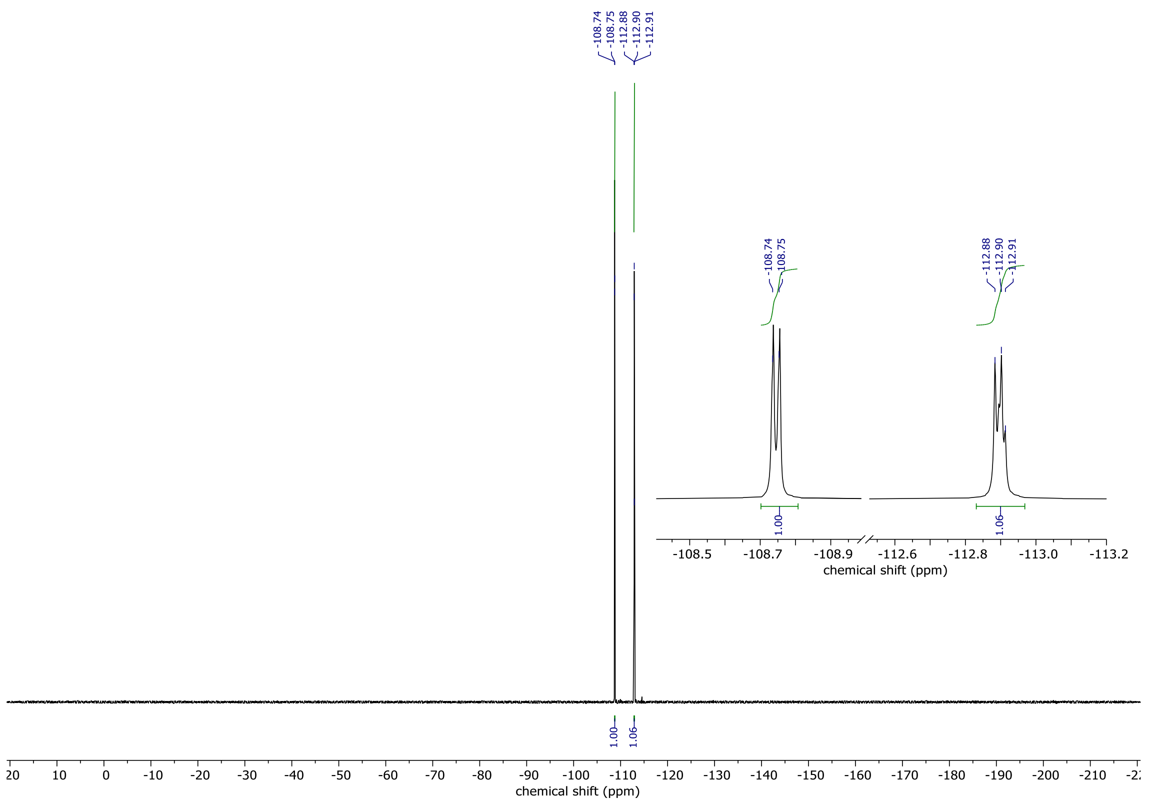

UV-VIS spectrum (recorded in 0.1M phosphate buffer pH 7.0)	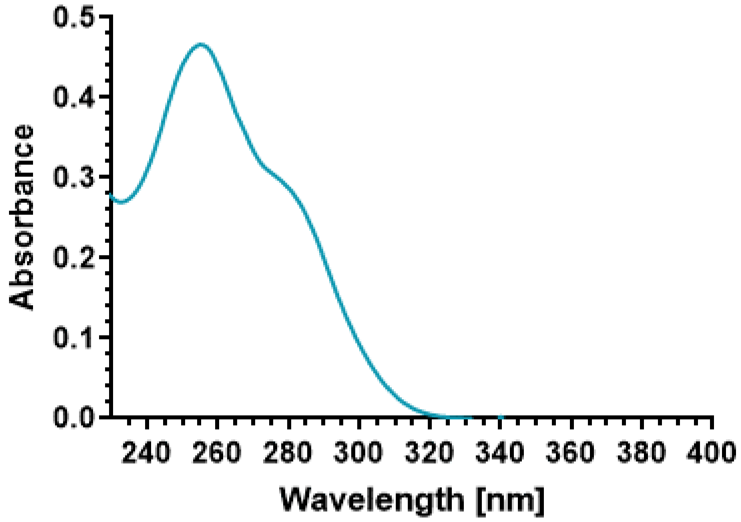

3,4-di-F-Bn^{7}GpppG (**1k**)	
Structure	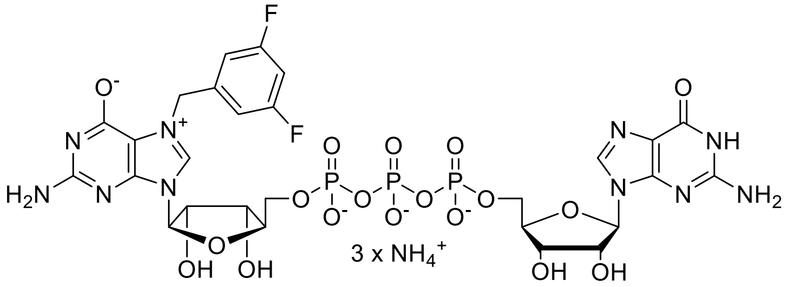
Reaction RP HPLC Profile	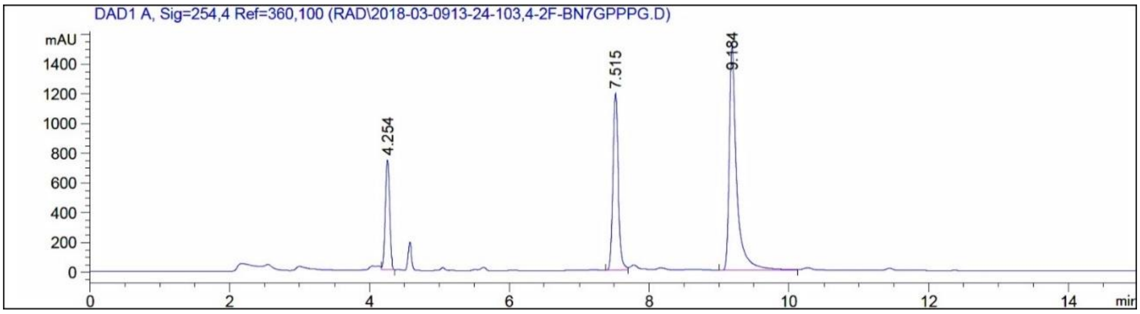
Purified Product RP HPLC Profile	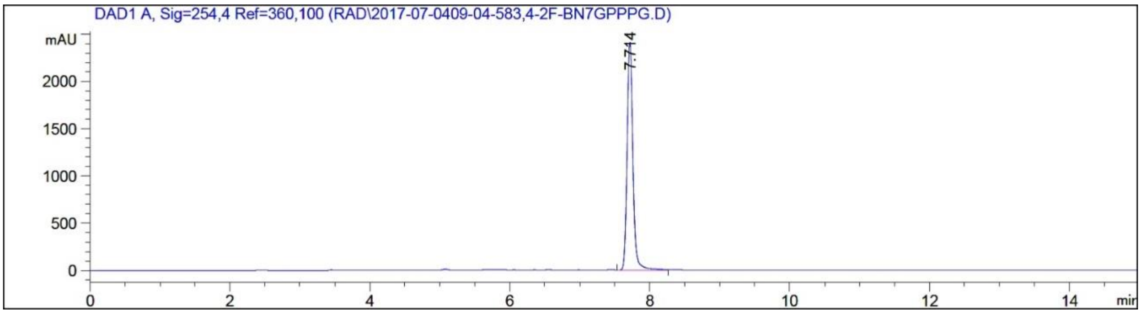
HRMS ES(-)	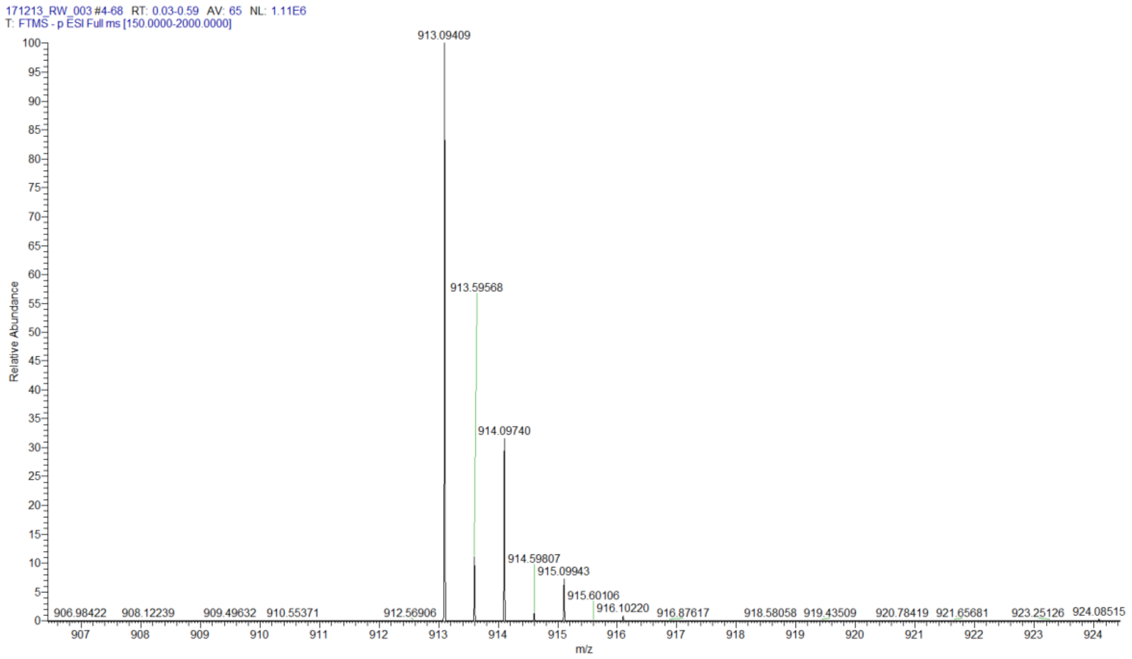

^{1}H NMR (500.24 MHz)	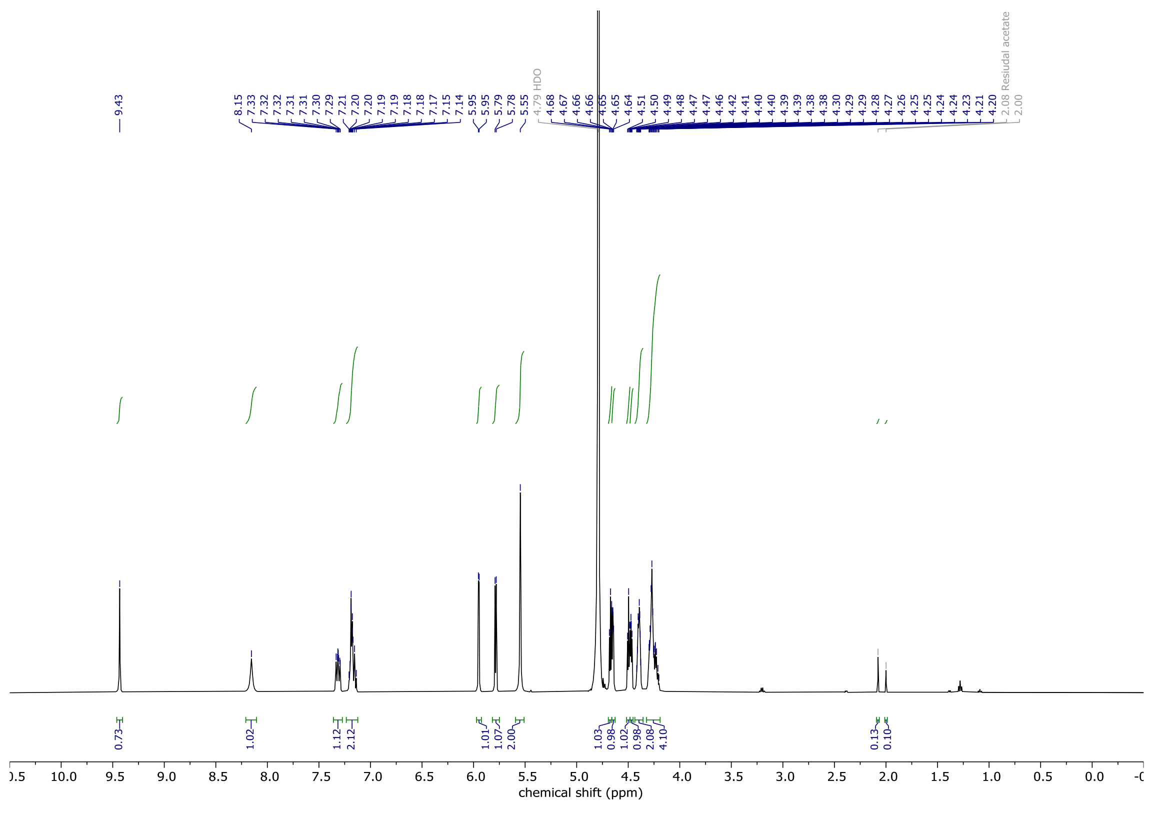
^{31}P NMR (202.49 MHz)	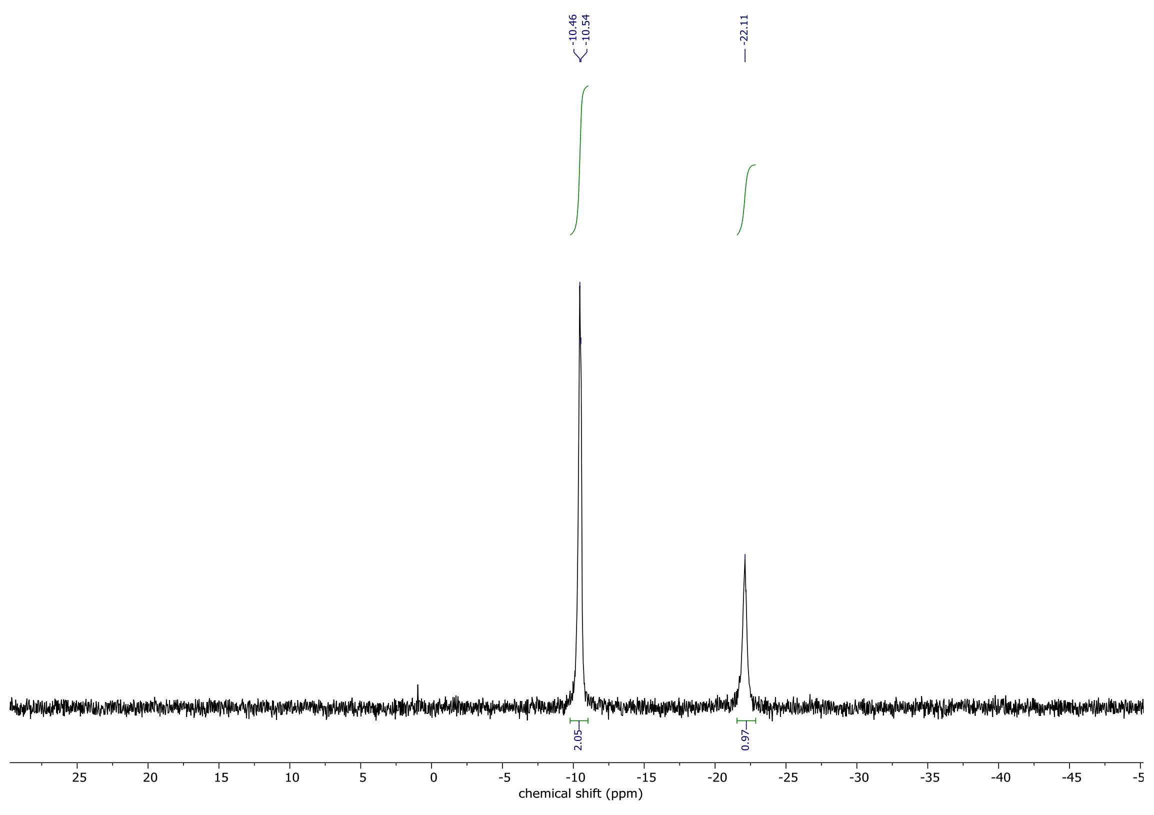

^{19}F NMR (470.65 MHz)

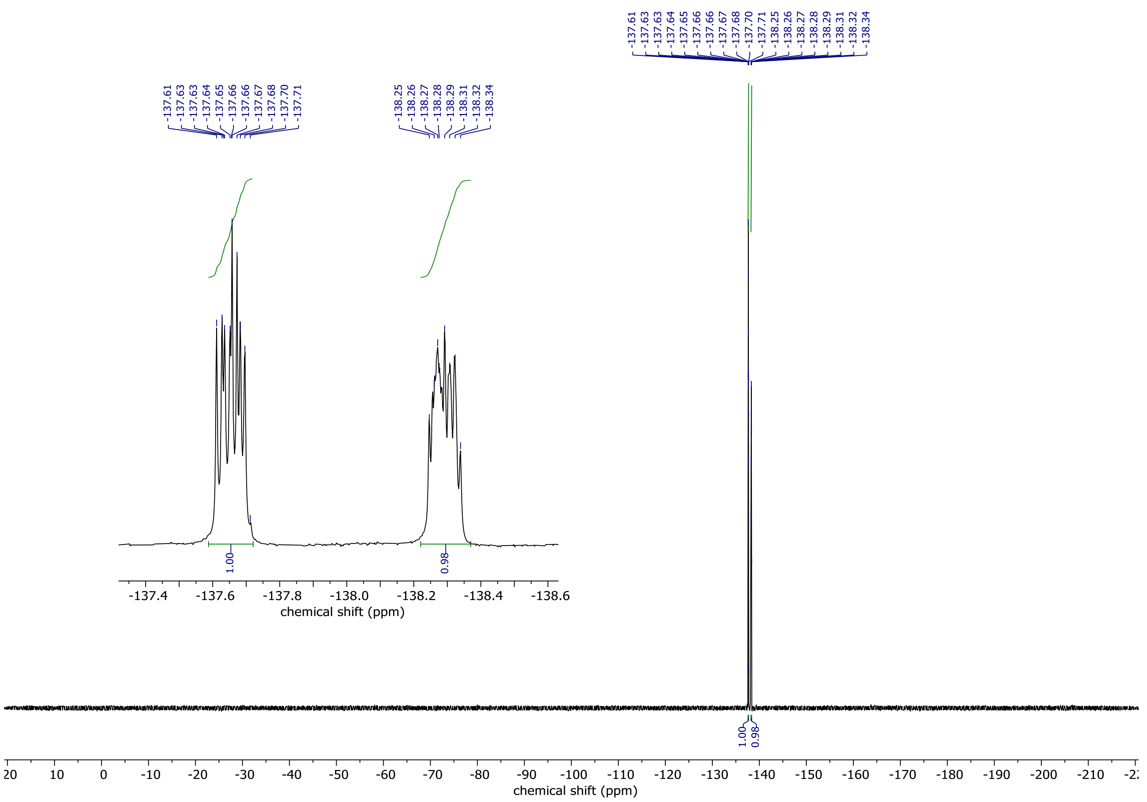

^{19}F {1H} NMR (470.65 MHz)

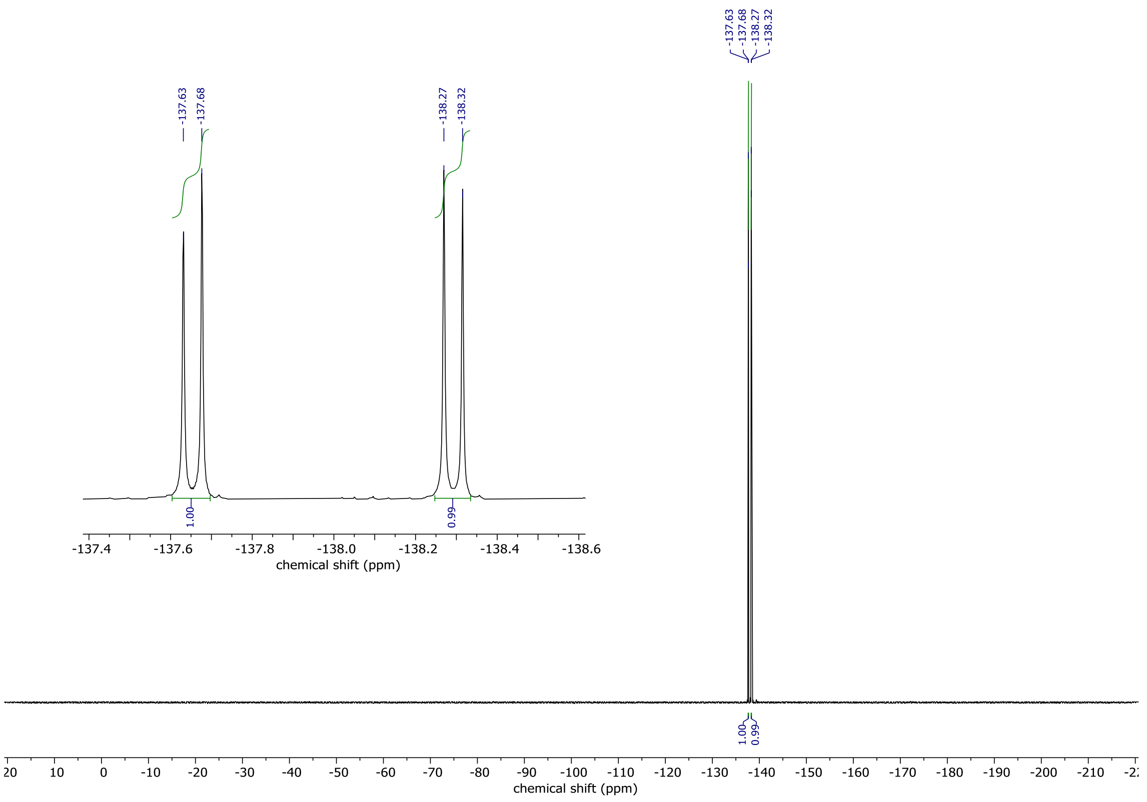

UV-VIS spectrum (recorded in 0.1M phosphate buffer pH 7.0)	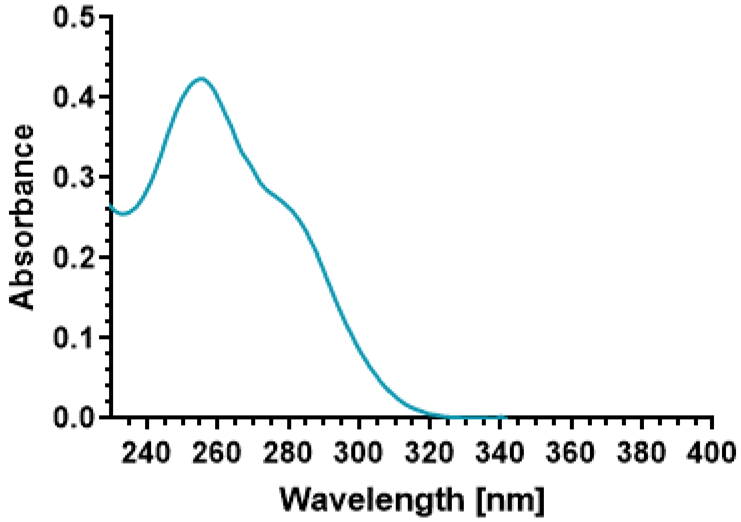

4-CF3-Bn7GpppG (**1l**)

Structure

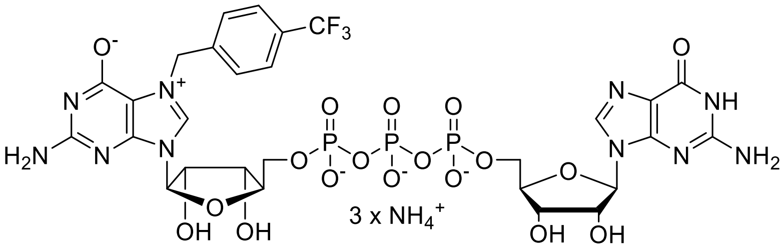

Reaction RP HPLC Profile

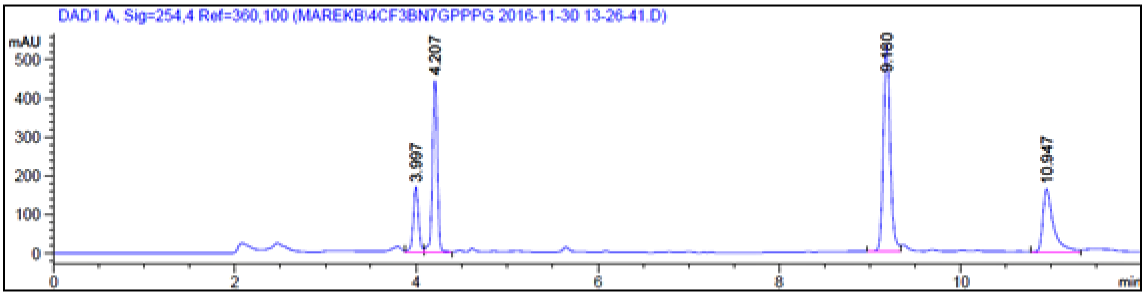

Purified Product RP HPLC Profile

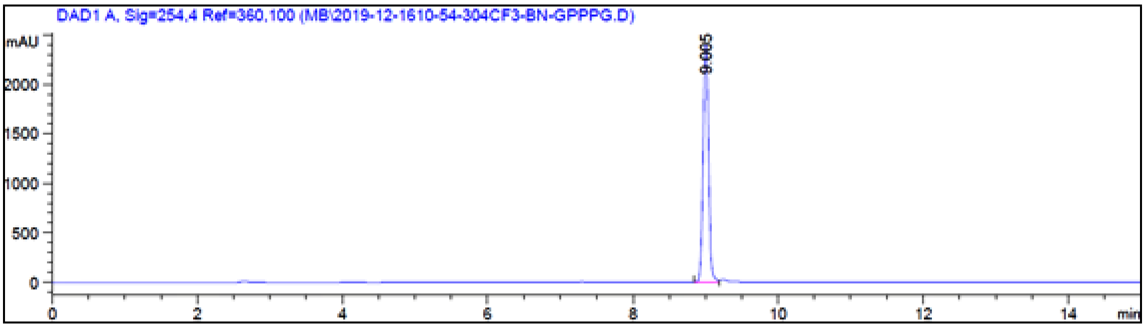

HRMS ES(-)

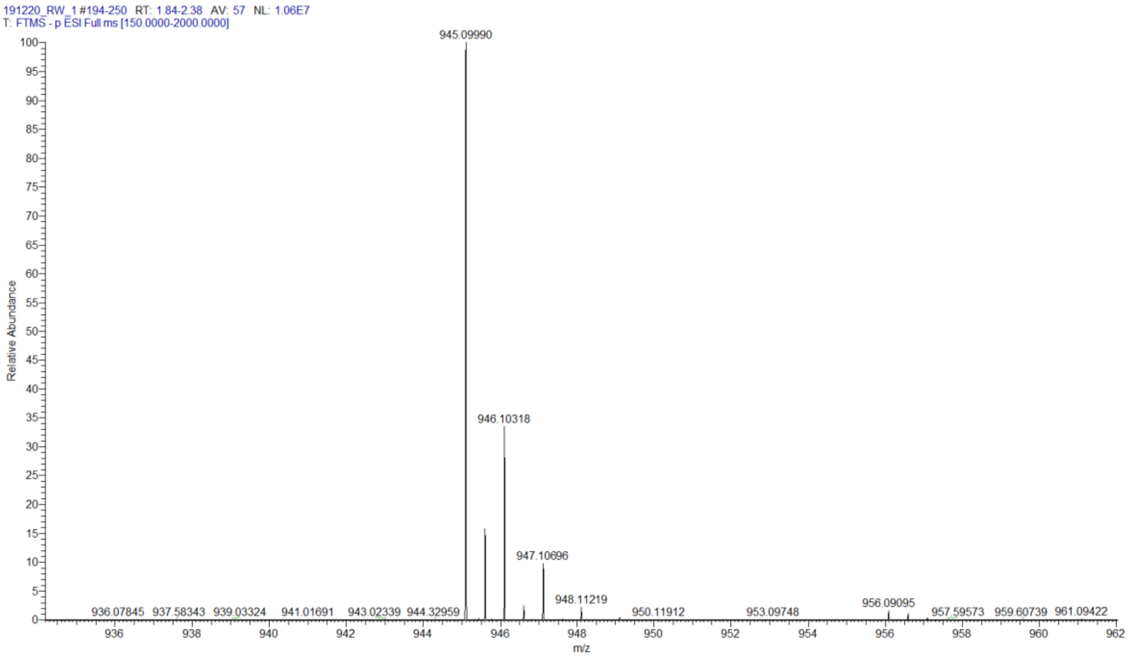

^{1}H NMR (500.24 MHz)	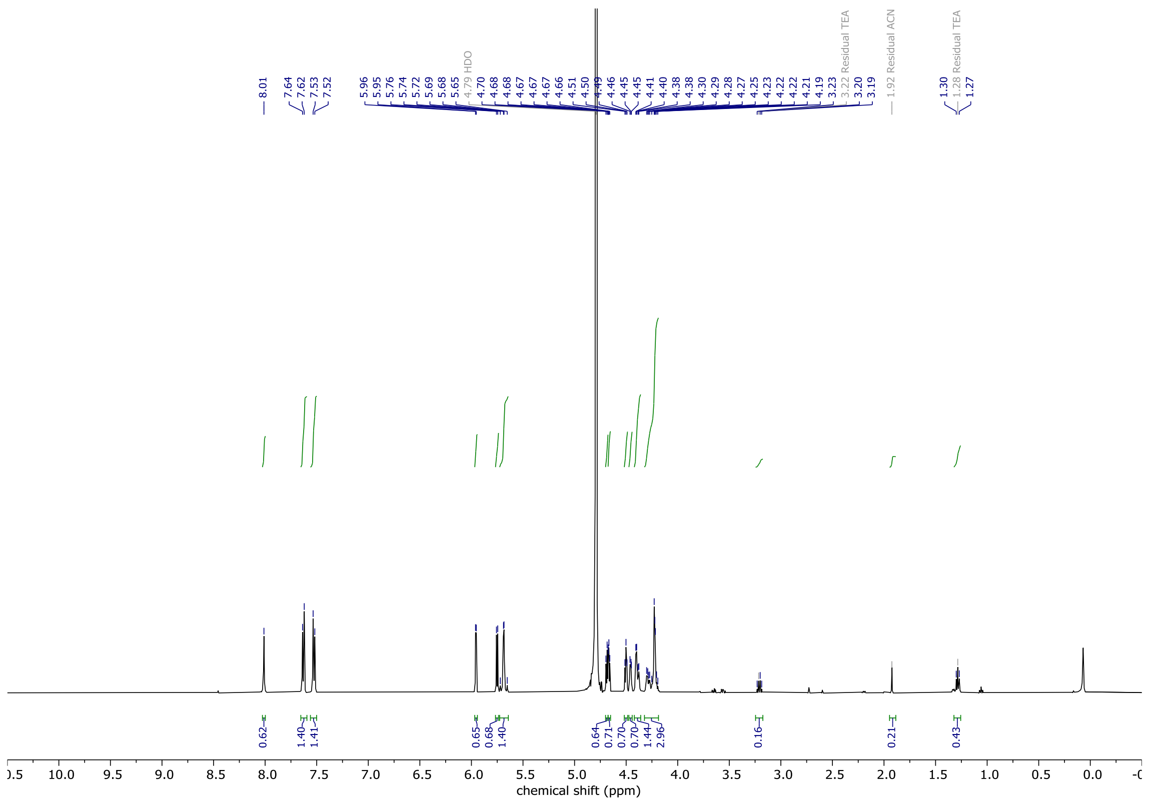
^{31}P NMR (202.49 MHz)	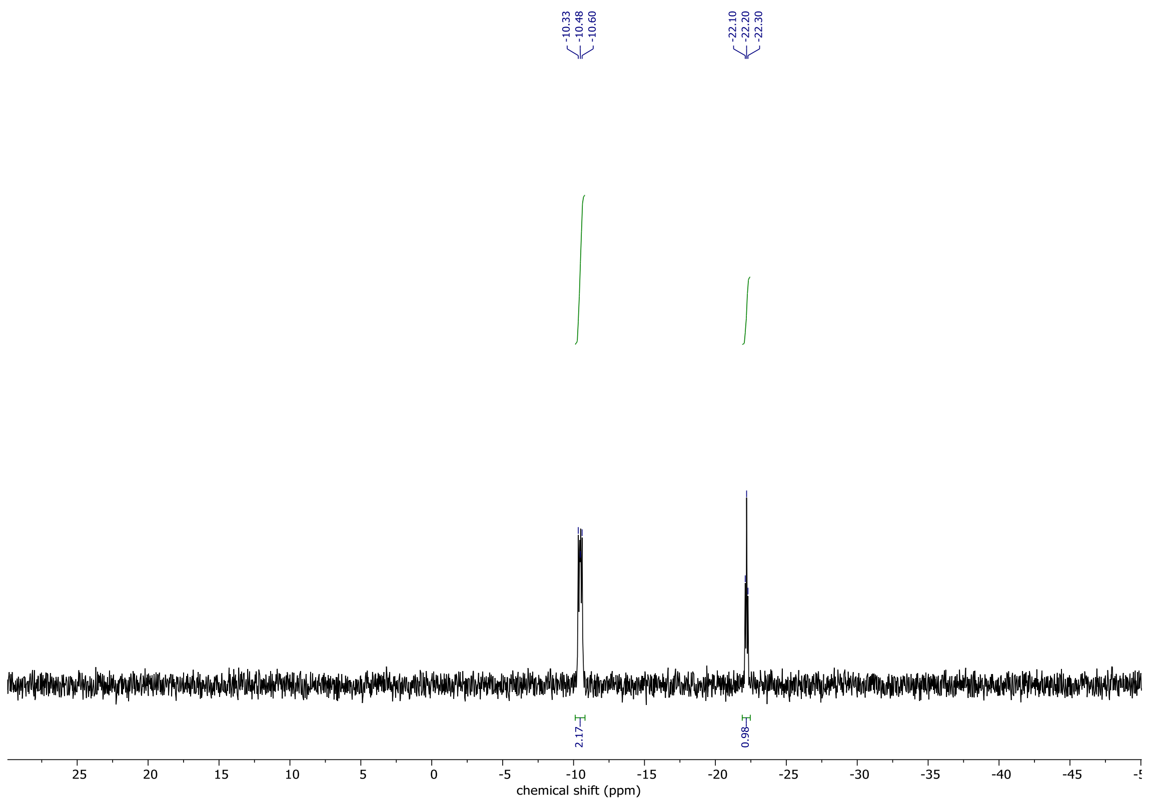

^{19}F NMR (470.65 MHz)	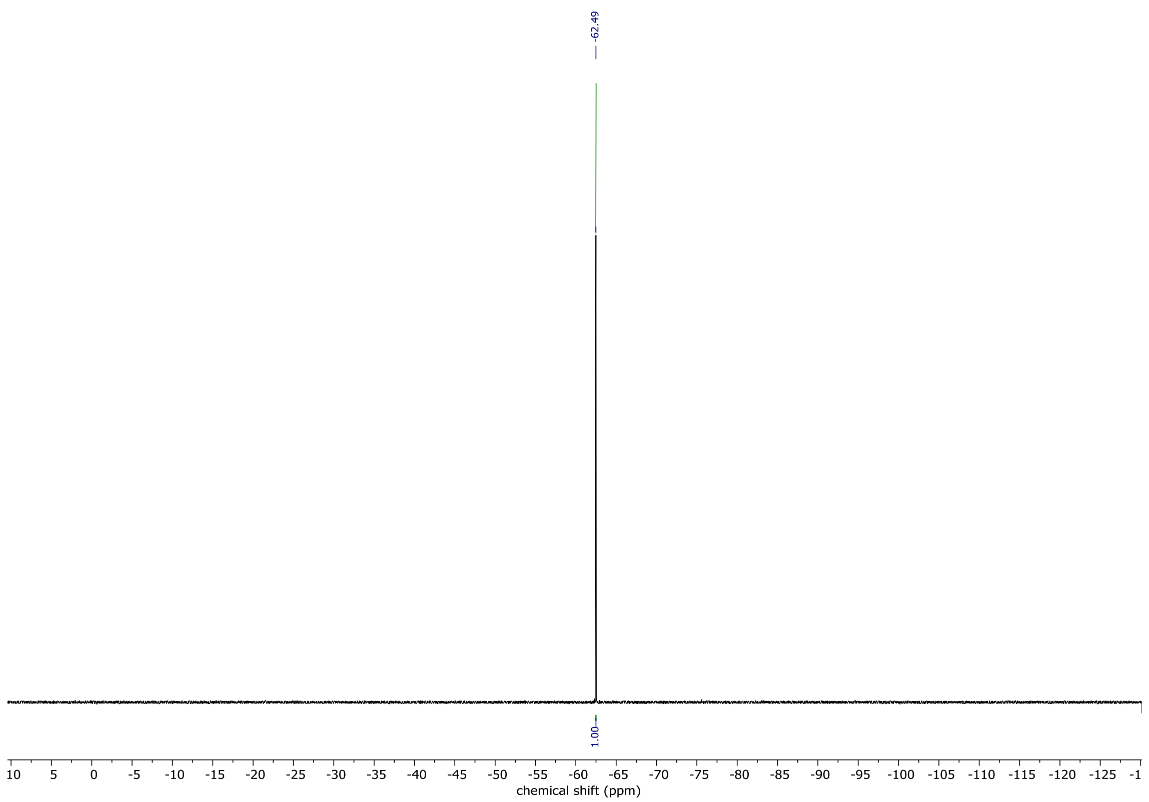
UV-VIS spectrum (recorded in 0.1M phosphate buffer pH 7.0)	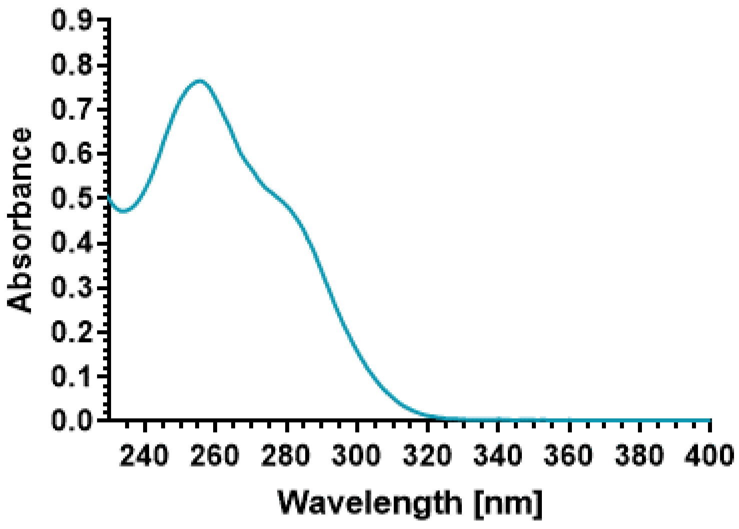

<table>
<tr><th colspan="2">4-COOH-Bn^{7}GpppG (1m)</th></tr>
<tr><td>Structure</td><td>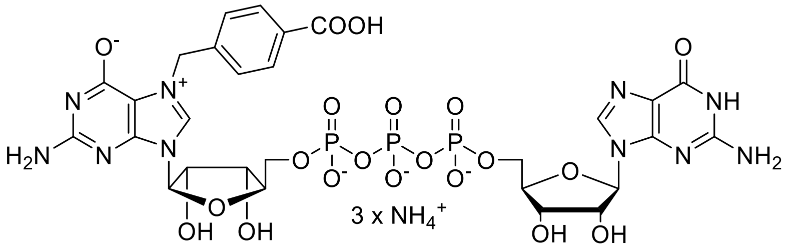
</td></tr>
<tr><td>Reaction RP HPLC Profile</td><td>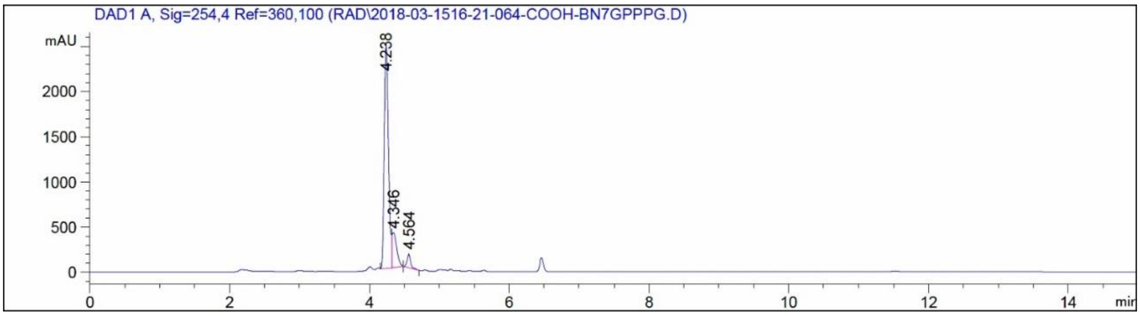
</td></tr>
<tr><td>Purified Product RP HPLC Profile</td><td>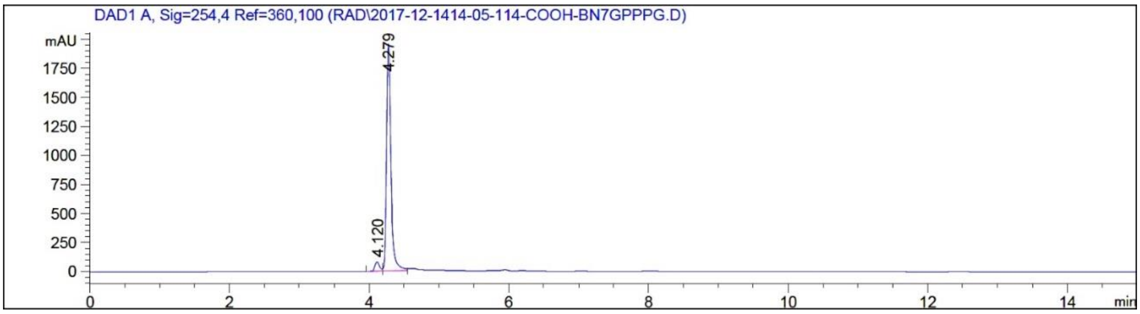
</td></tr>
<tr><td>HRMS ES(-)</td><td>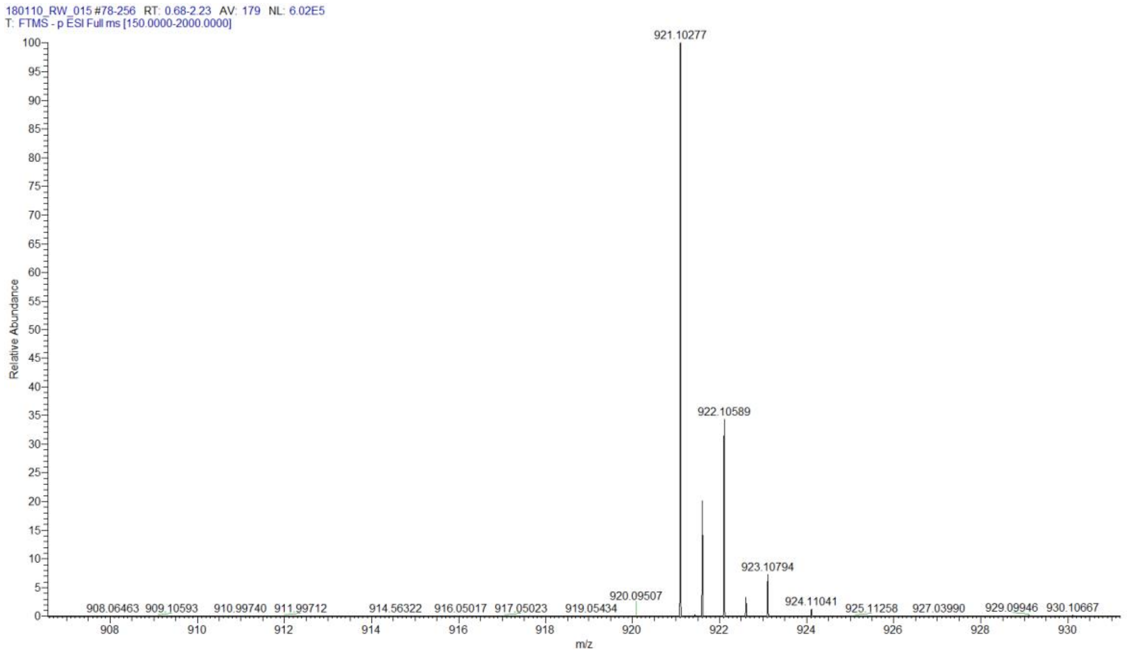
</td></tr>
</table>

^{1}H NMR (500.24 MHz)

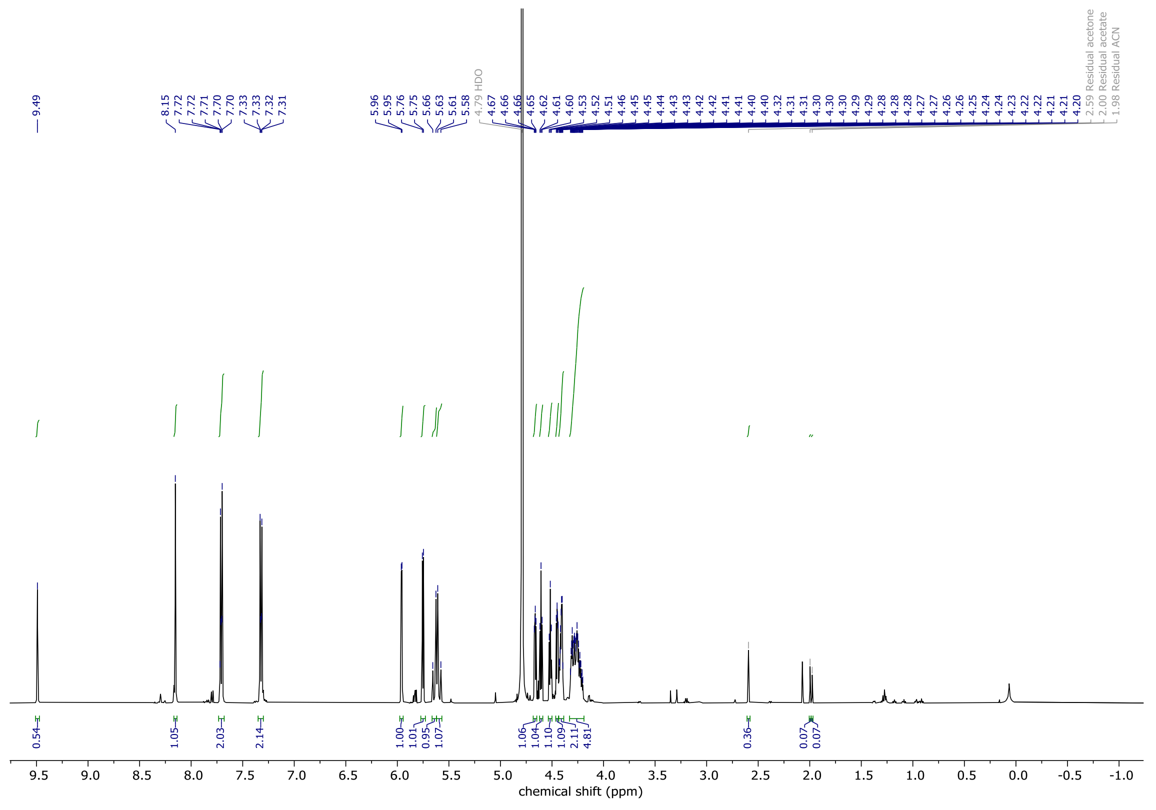

^{31}P NMR (202.49 MHz)

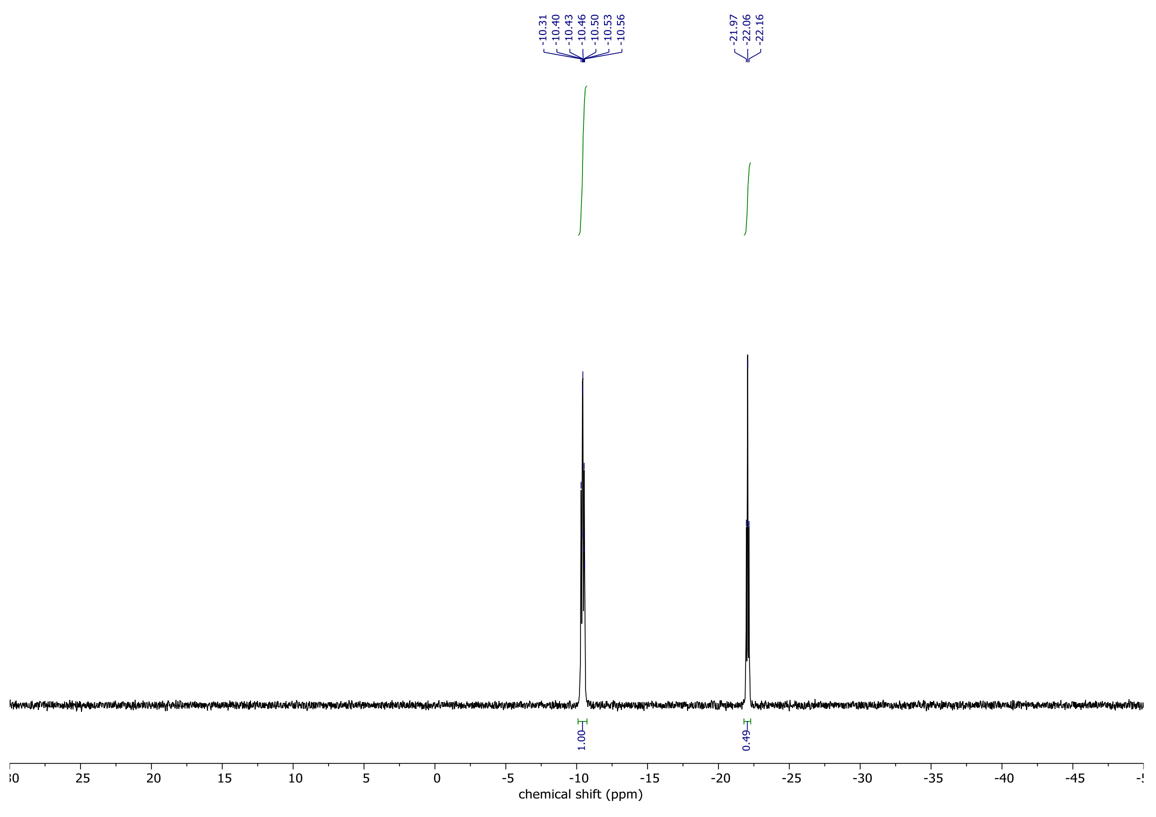

UV-VIS spectrum (recorded in 0.1M phosphate buffer pH 7.0)	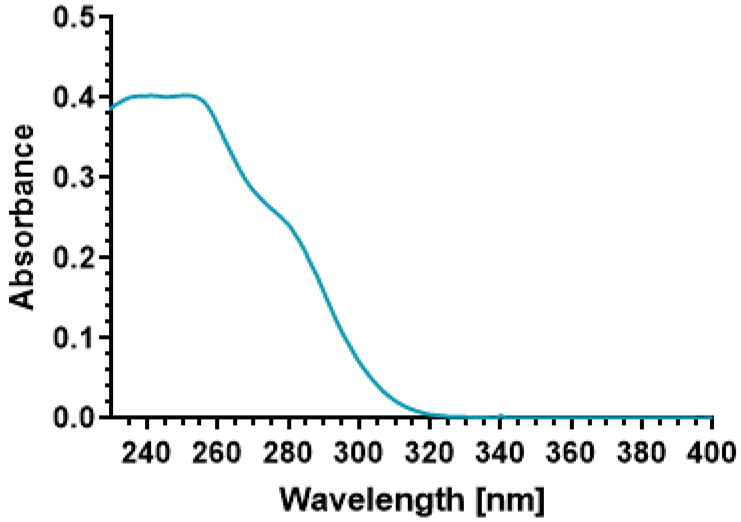

4-NO_2-Bn^7GpppG (**1n**)	
Structure	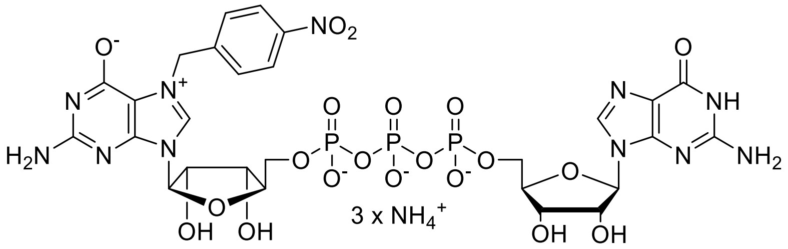
Reaction RP HPLC Profile	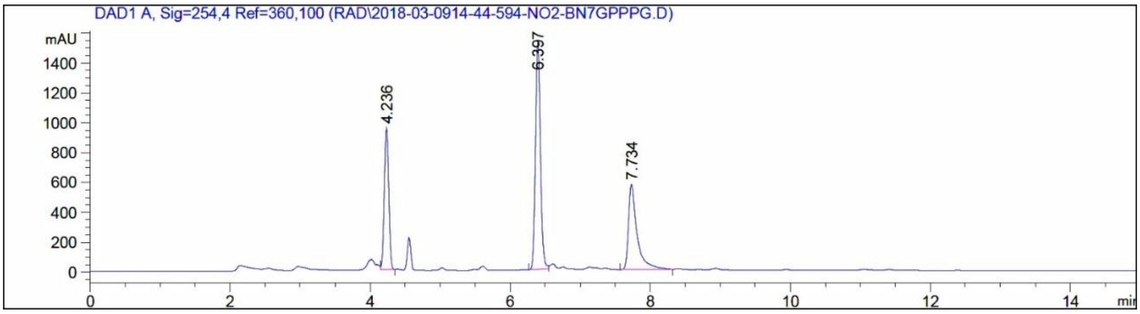
Purified Product RP HPLC Profile	’ 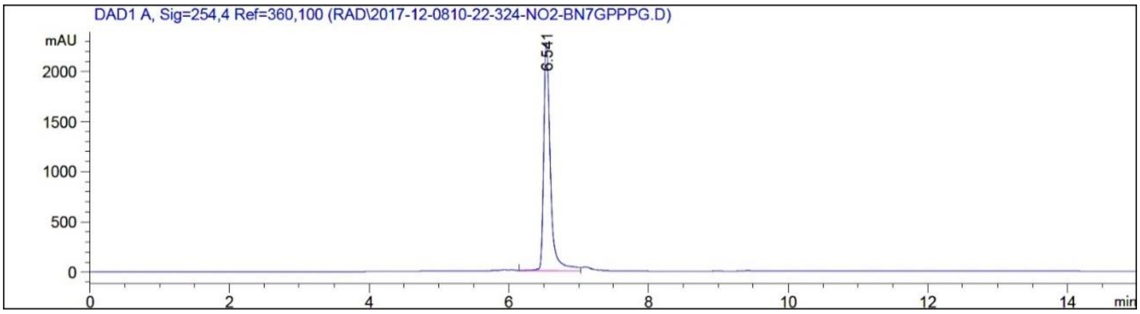
HRMS ES(-)	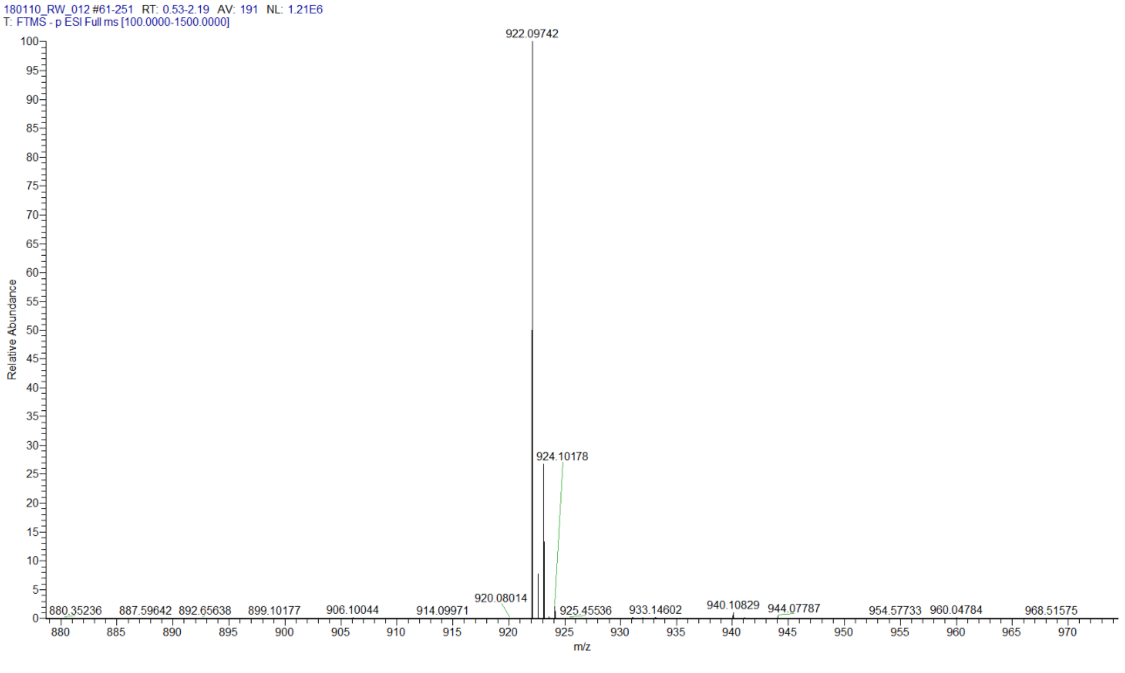

^{1}H NMR (500.24 MHz)	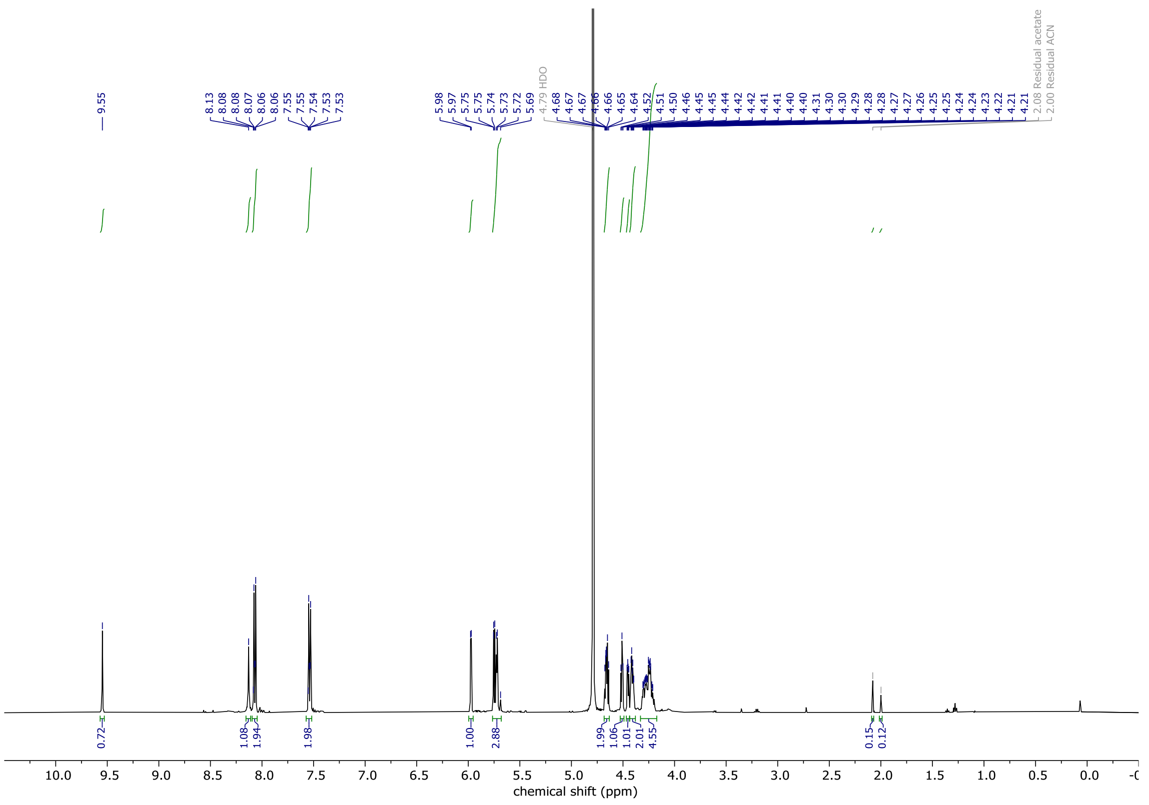
^{31}P NMR (202.49 MHz)	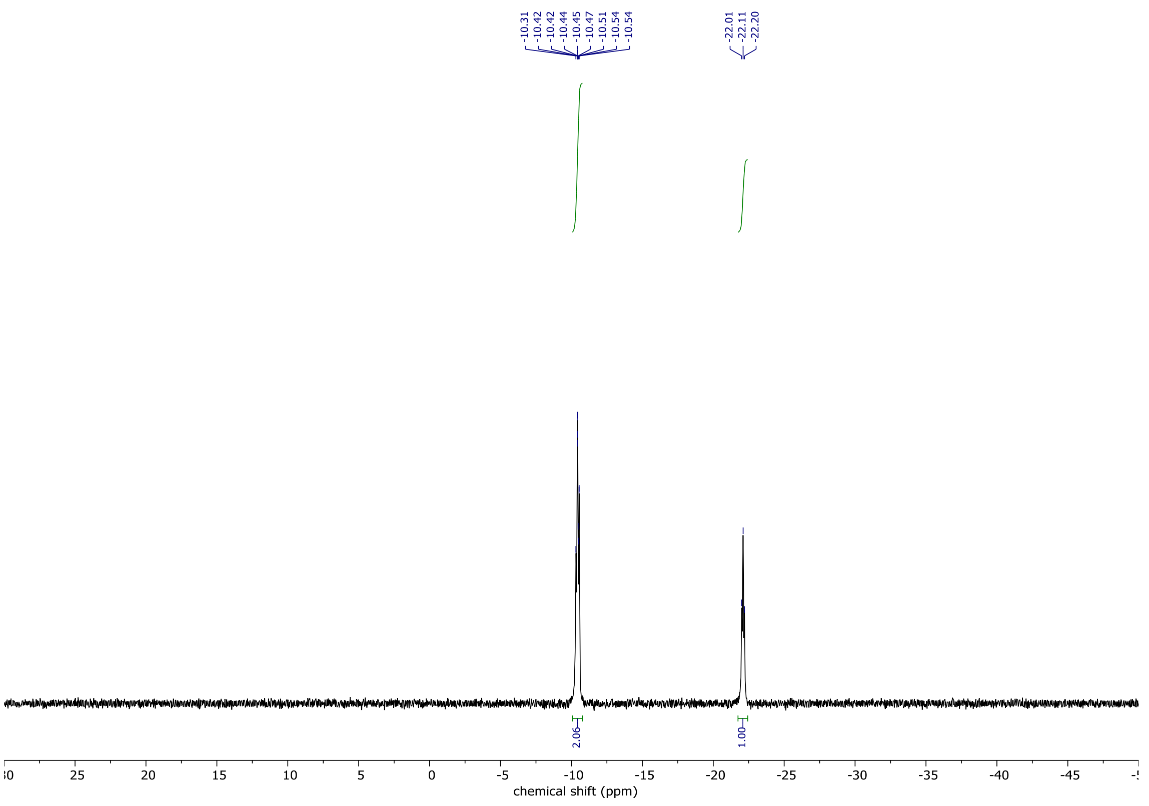

UV-VIS spectrum (recorded in 0.1M phosphate buffer pH 7.0)	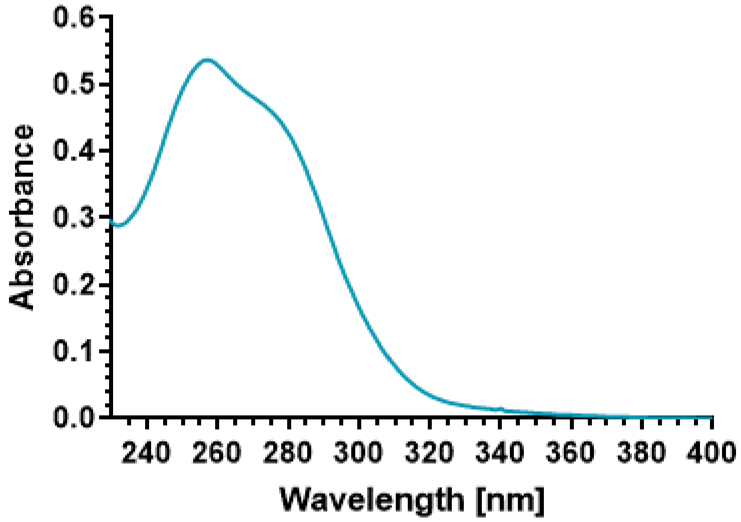

α-Naphm⁷GpppG (**1o**)	
Structure	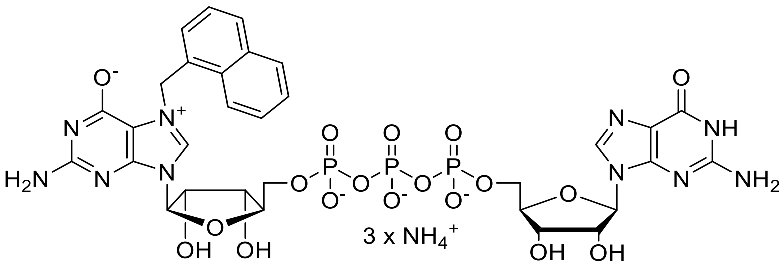
Reaction RP HPLC Profile	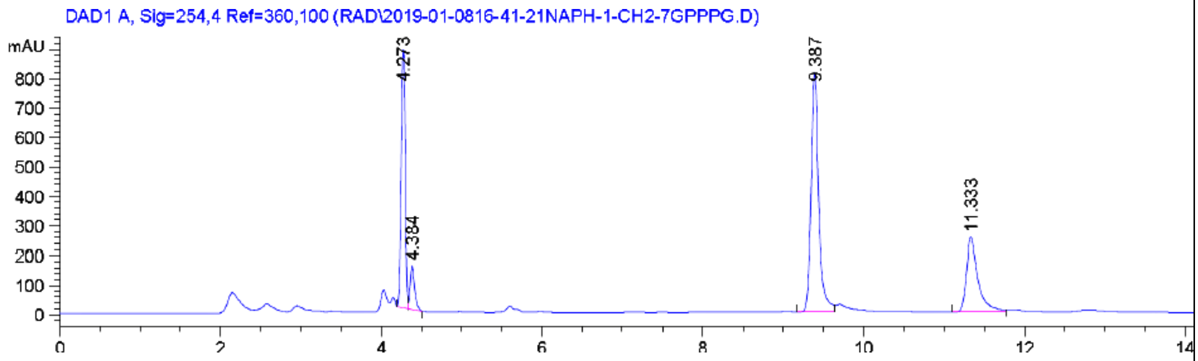
Purified Product RP HPLC Profile	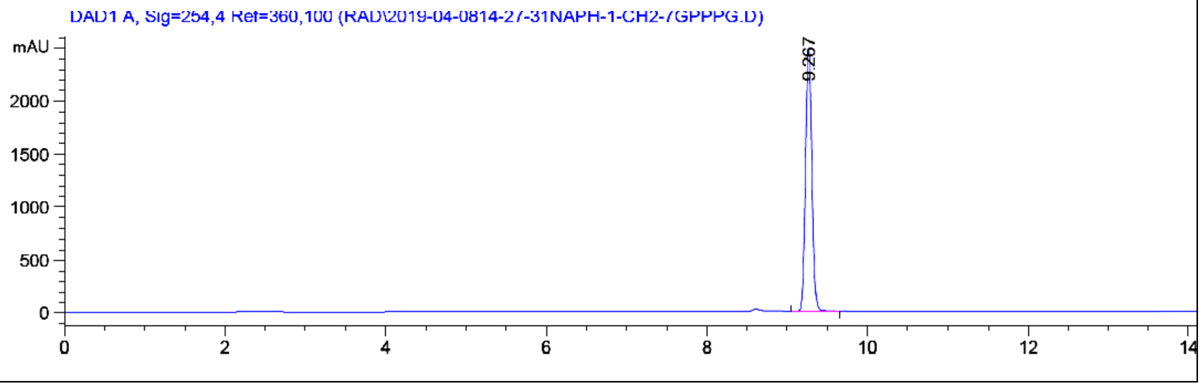

HRMS ES(-)	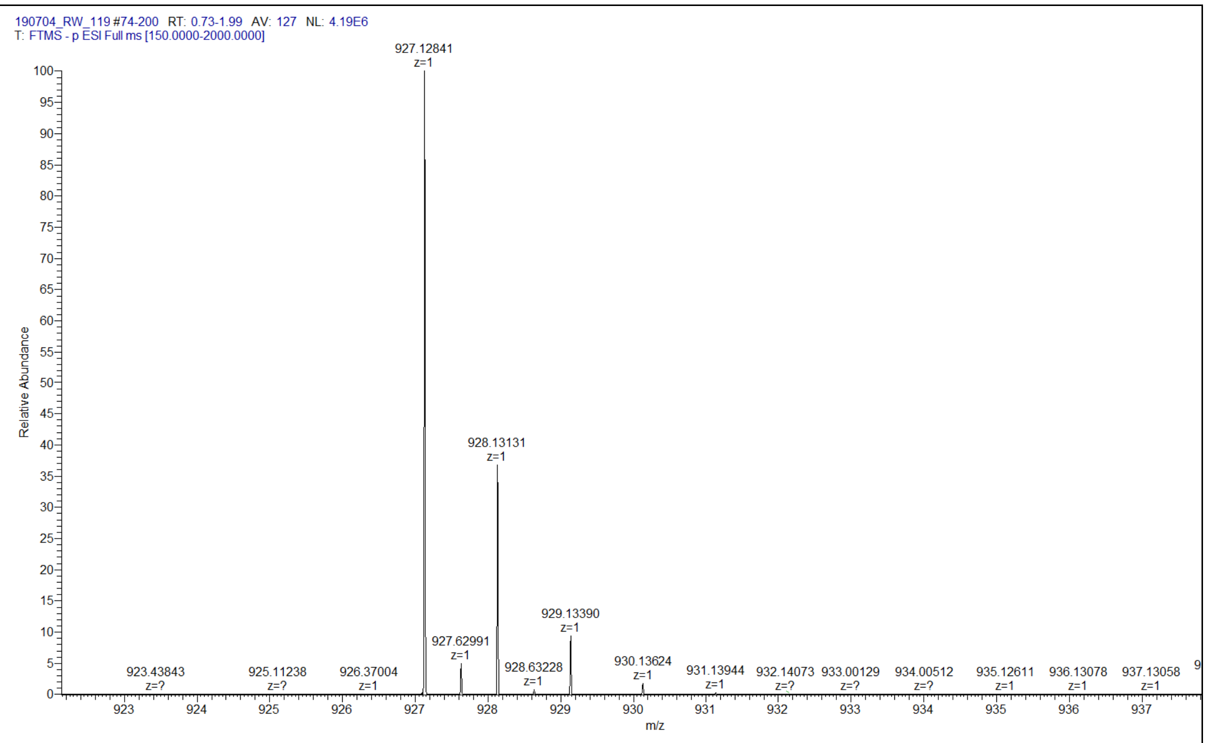
1H NMR (500.24 MHz)	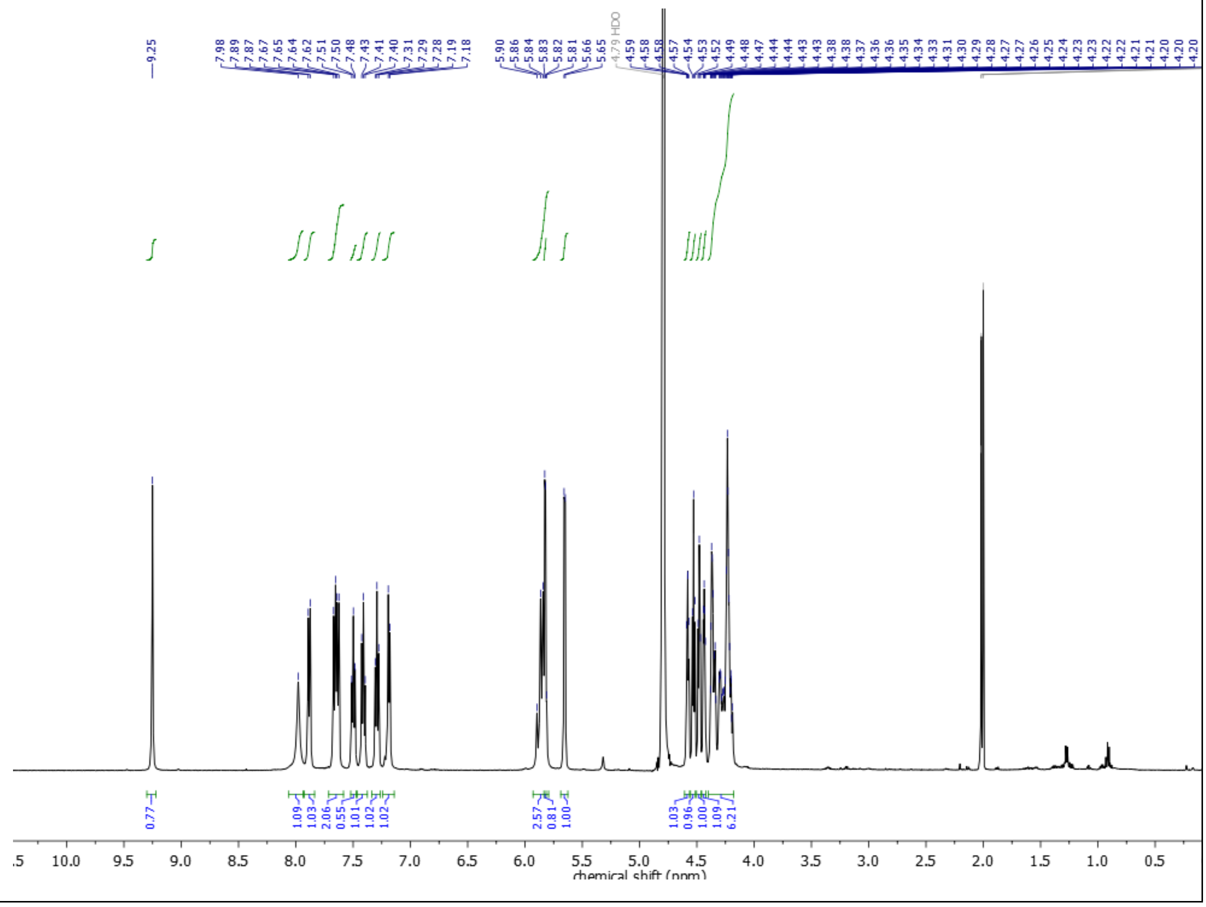

^{31}P NMR (202.49 MHz)	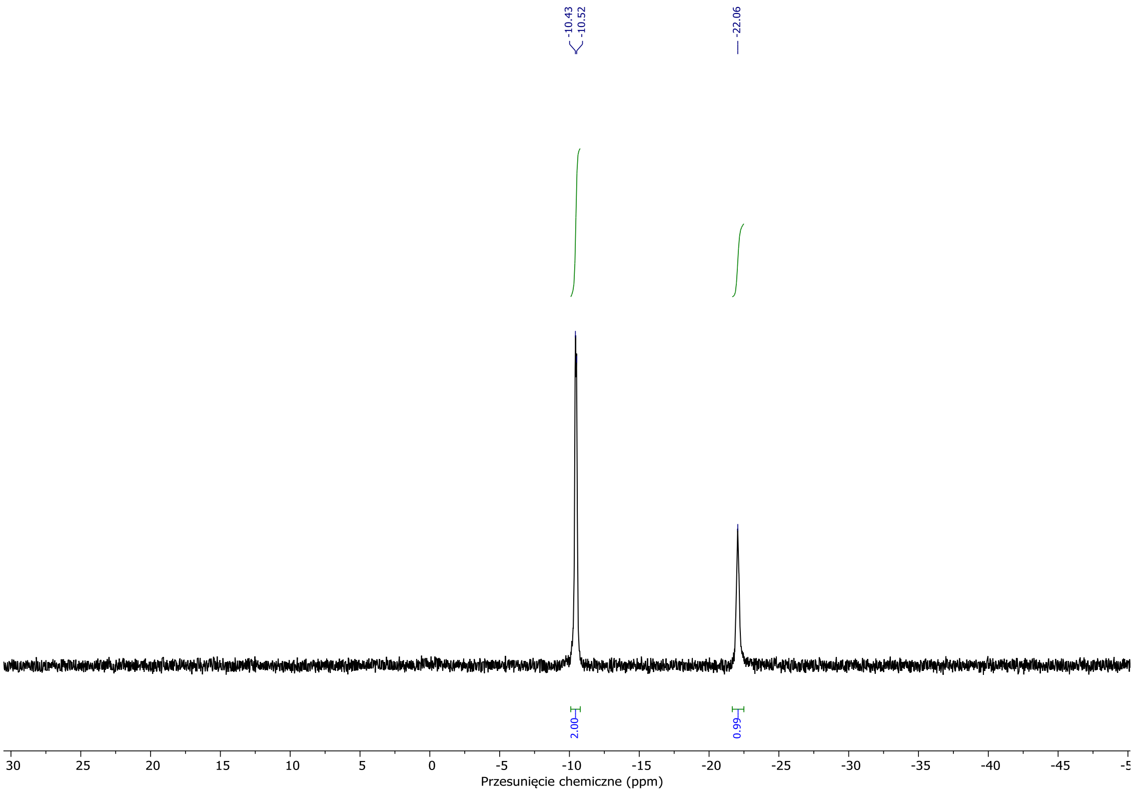
UV-VIS spectrum (recorded in 0.1M phosphate buffer pH	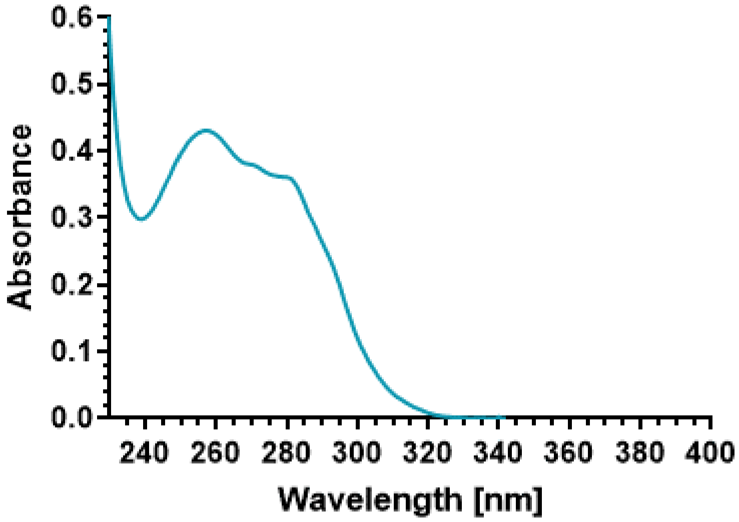

β-Naphm^{7}GpppG (**1p**)	
Structure	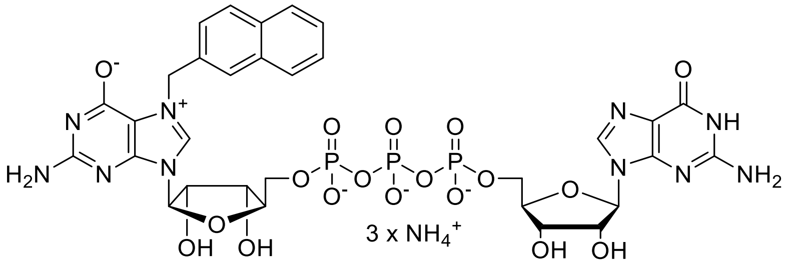
Reaction RP HPLC Profile	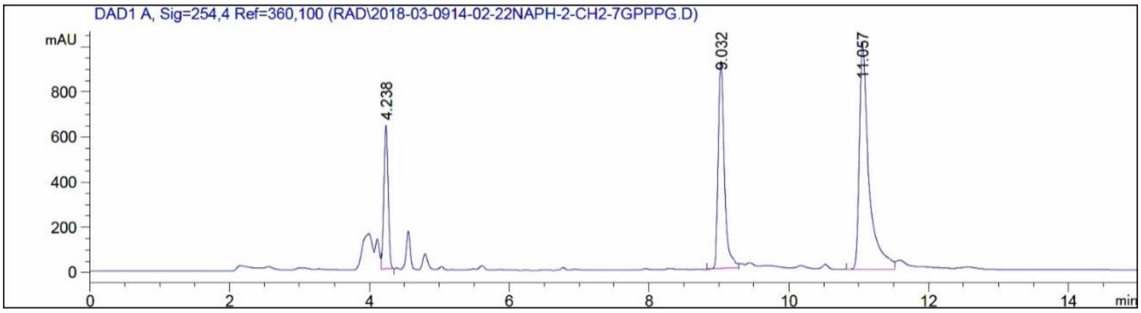
Purified Product RP HPLC Profile	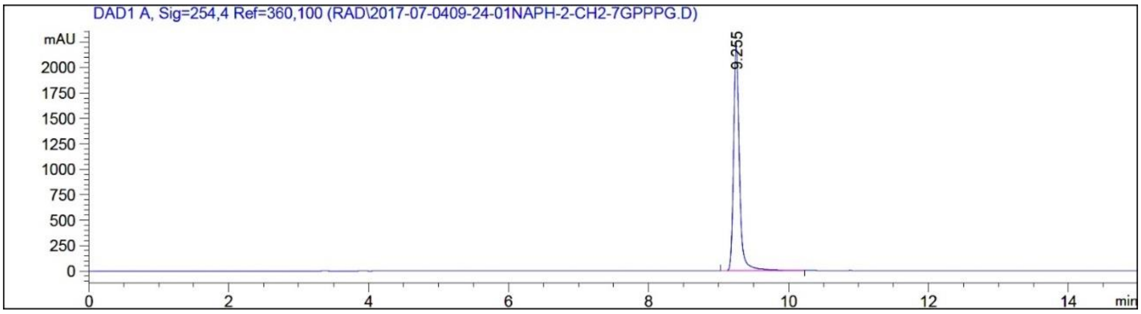
HRMS ES(-)	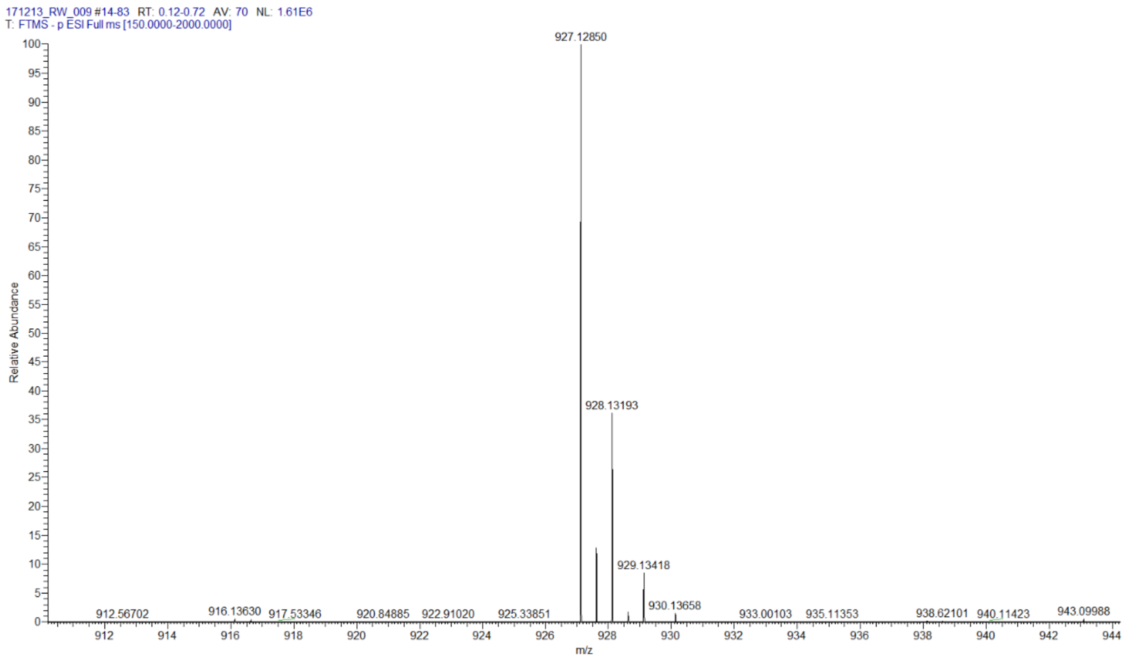

^{1}H NMR (500.24 MHz)	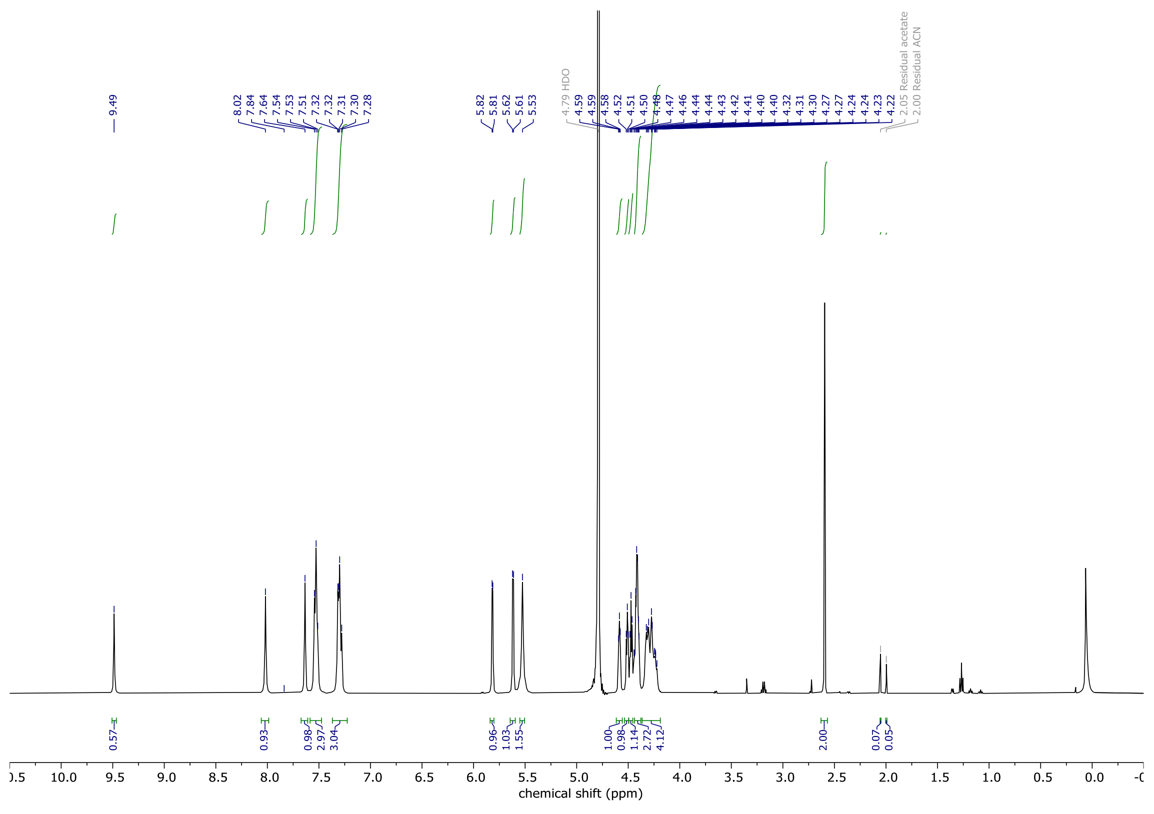
^{31}P NMR (202.49 MHz)	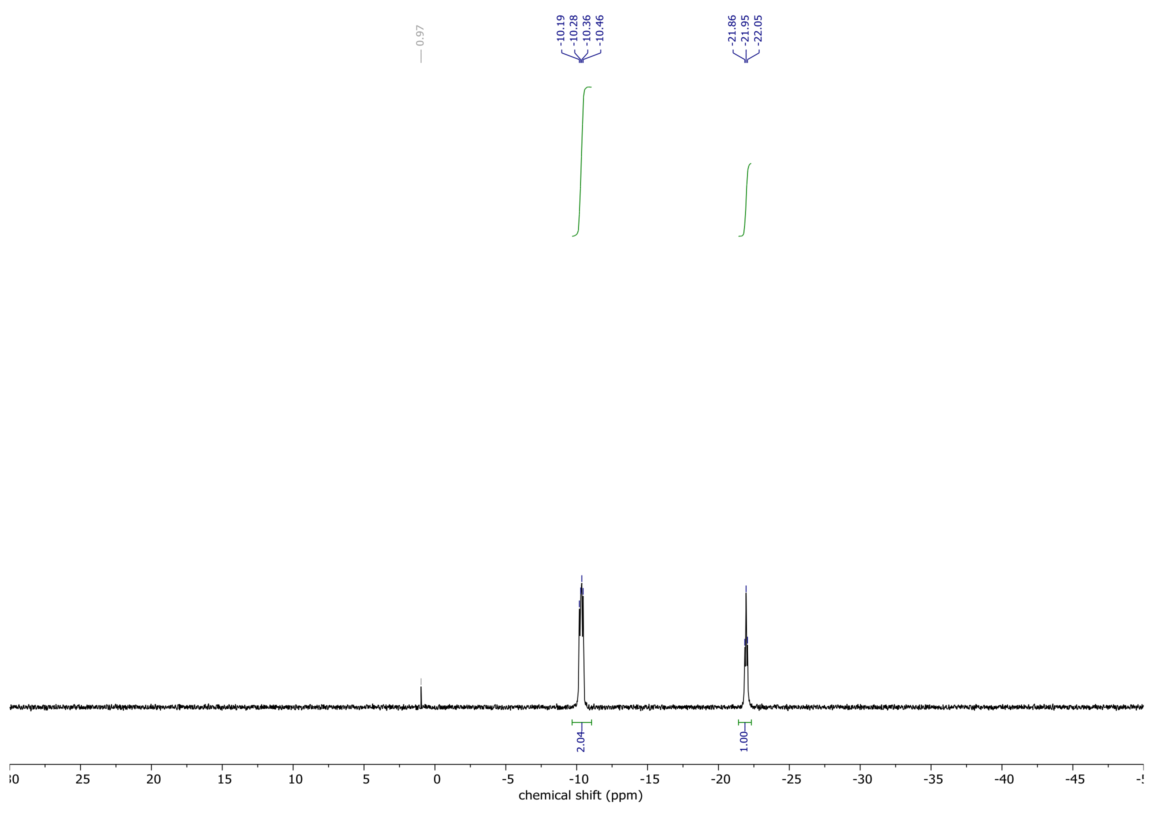

UV-VIS spectrum (recorded in 0.1M phosphate buffer pH 7.0)	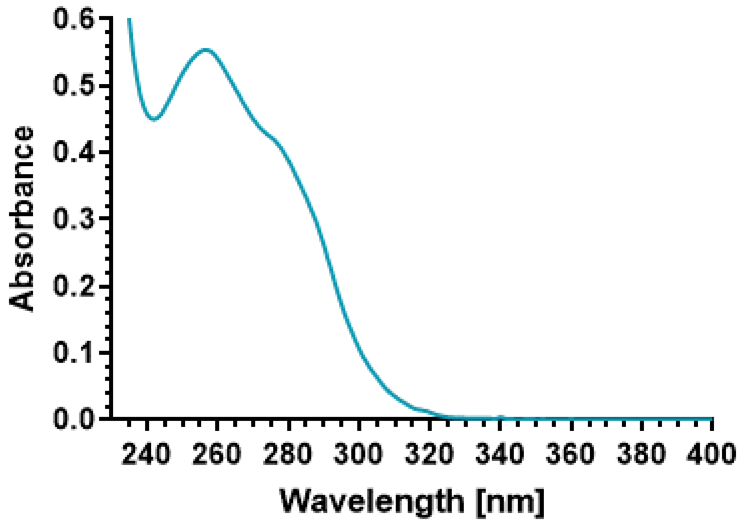

Bn^{7}G$_m$pppG (**1q**)	
Structure	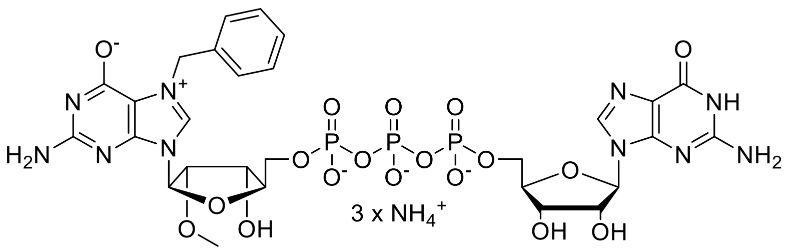
Reaction RP HPLC Profile	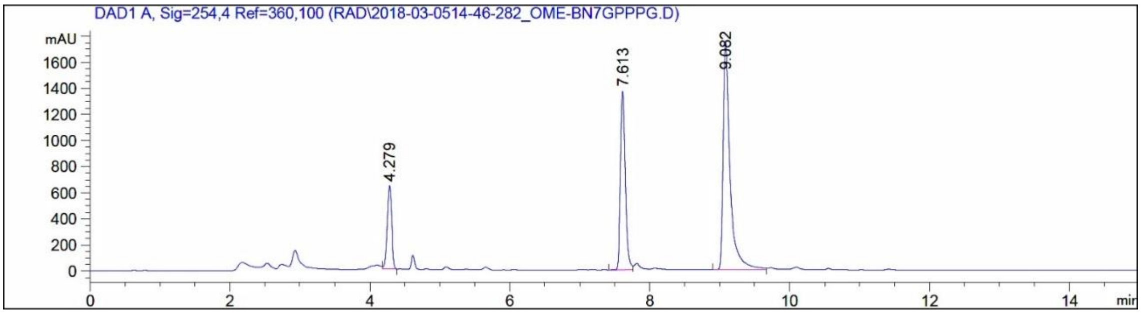
Purified Product RP HPLC Profile	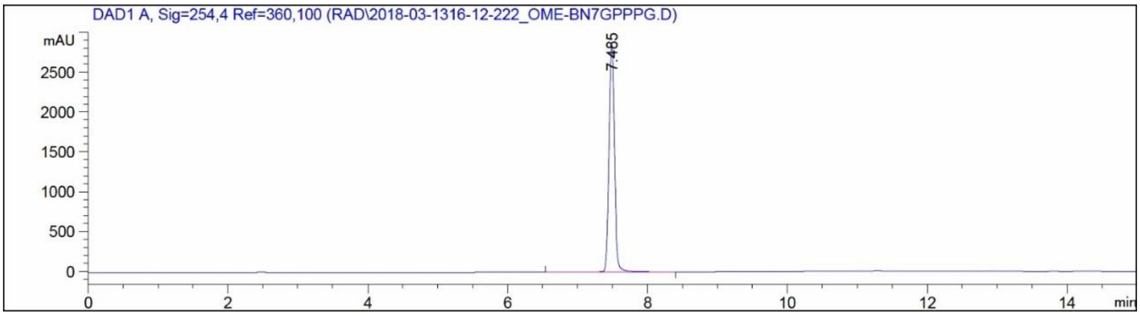
HRMS ES(-)	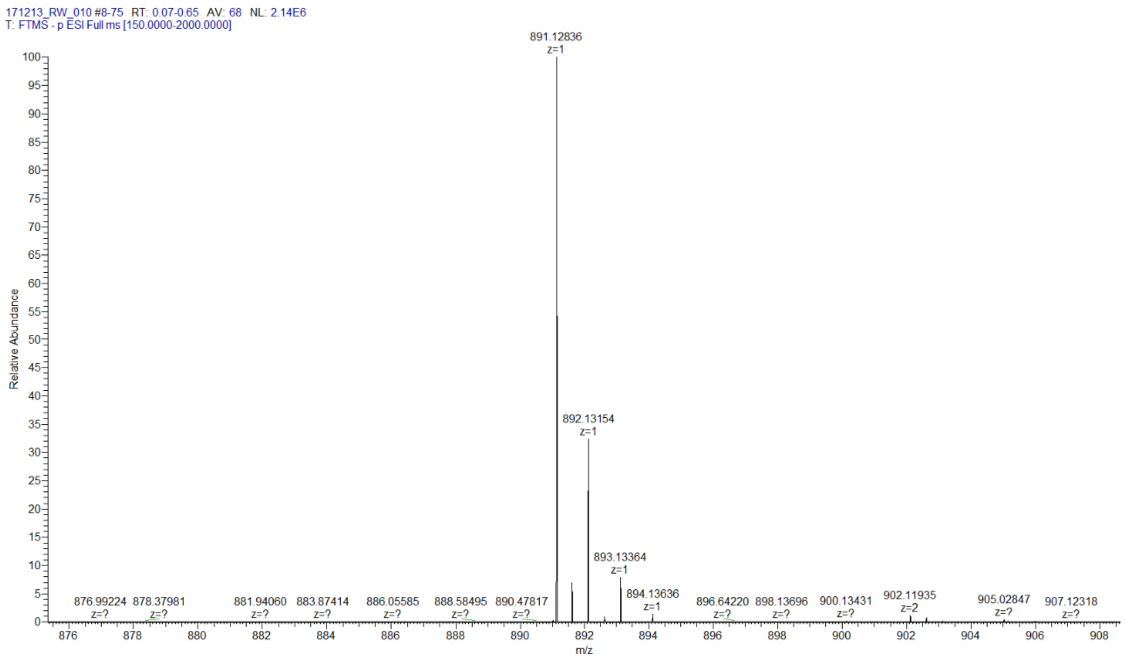

^{1}H NMR (500.24 MHz)

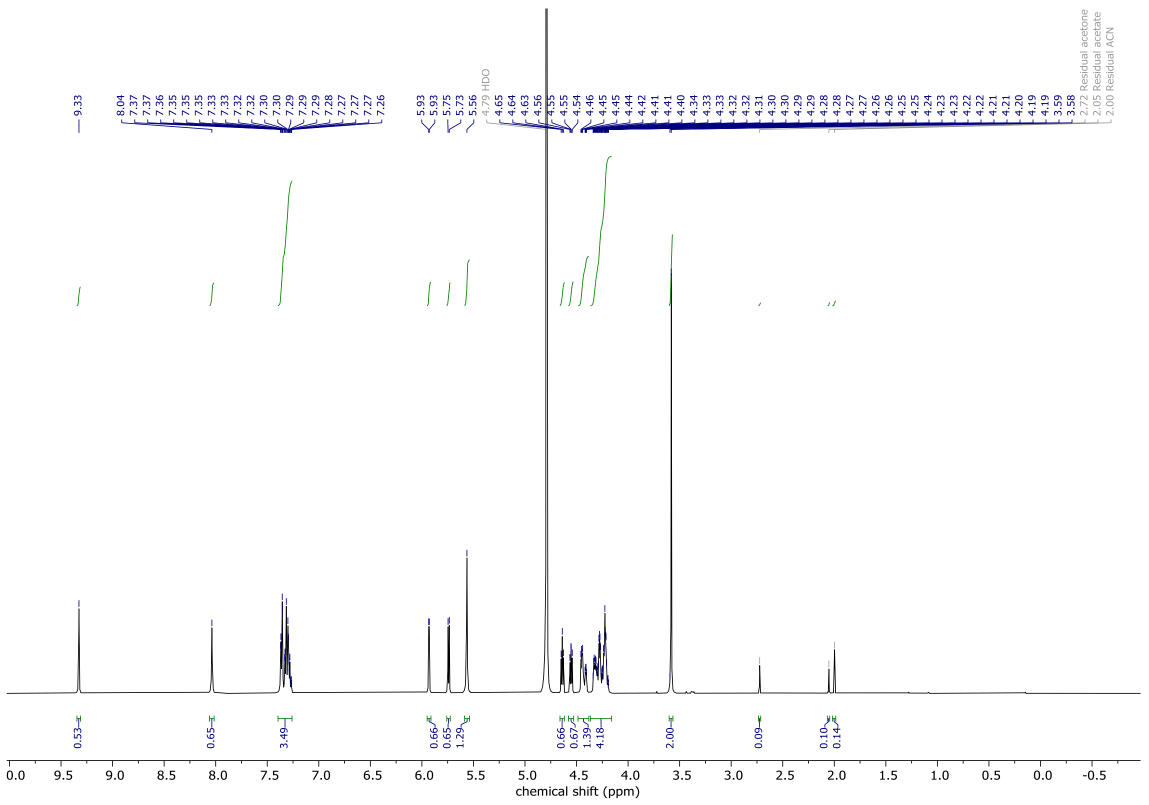

^{31}P NMR (202.49 MHz)

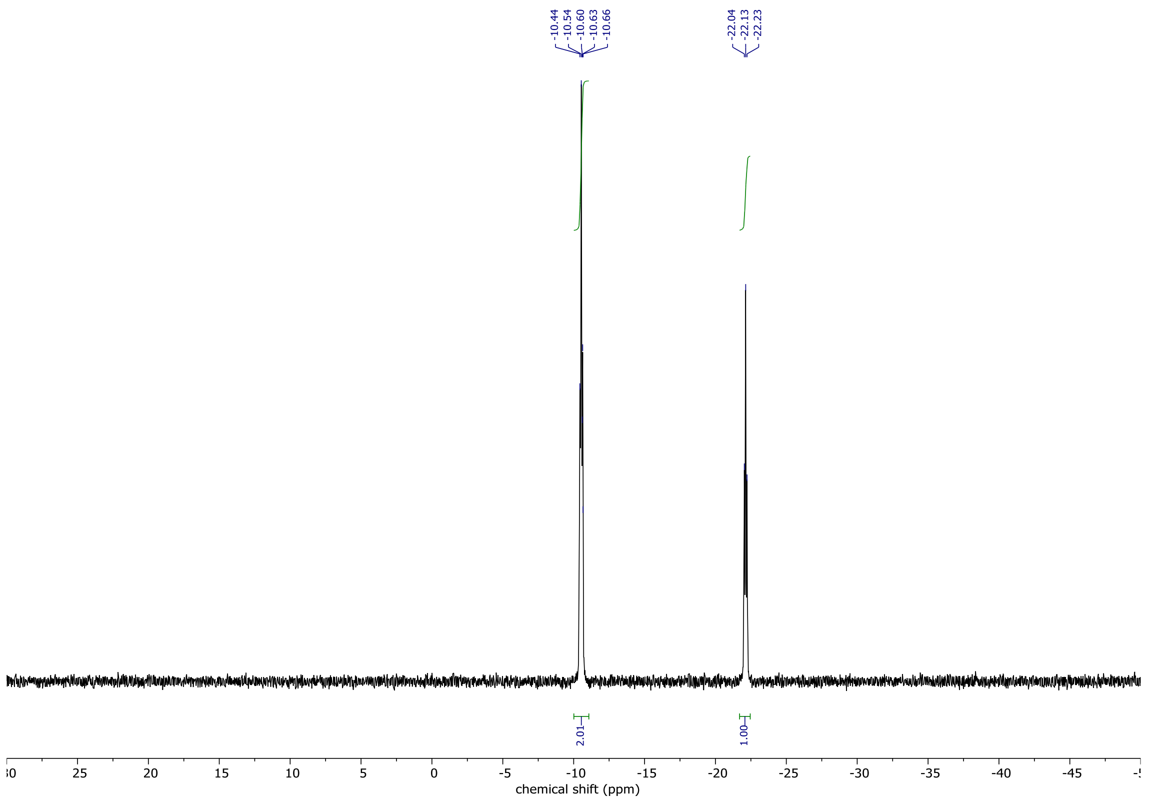

UV-VIS spectrum (recorded in 0.1M phosphate buffer pH 7.0)	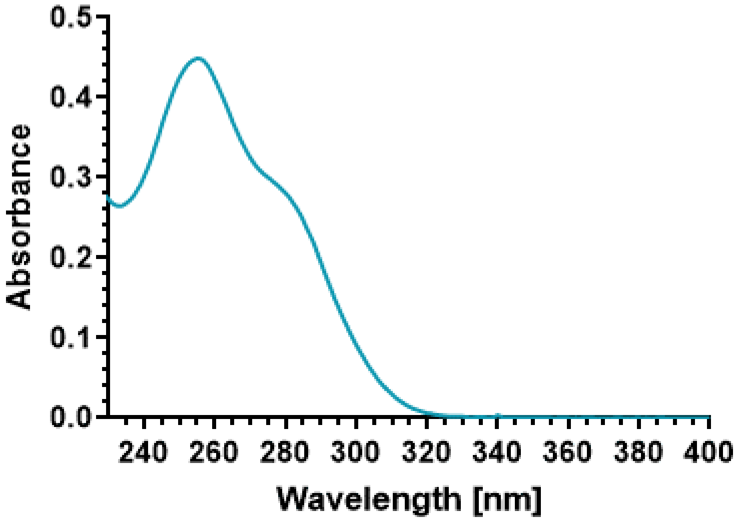

<table>
<tr><th colspan="2">4-Cl-Bn^{7}G$_{m}$pppG (1r)</th></tr>
<tr><td>Structure</td><td>
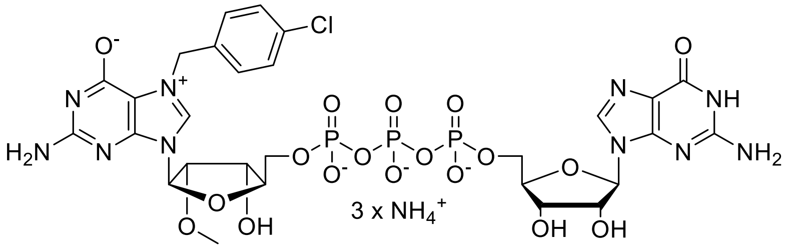

</td></tr>
<tr><td>Reaction RP HPLC Profile</td><td>
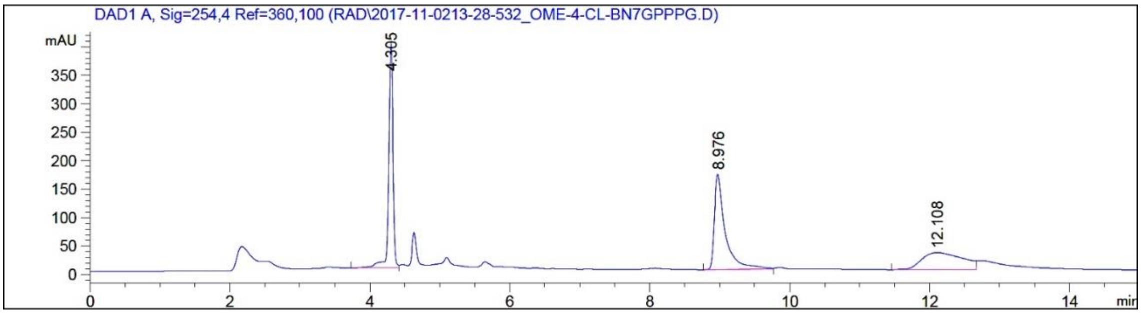

</td></tr>
<tr><td>Purified Product RP HPLC Profile</td><td>
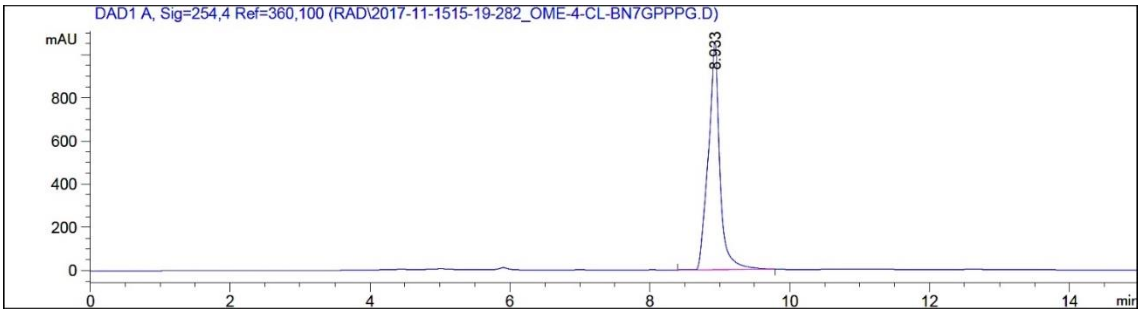

</td></tr>
<tr><td>HRMS ES(-)</td><td>
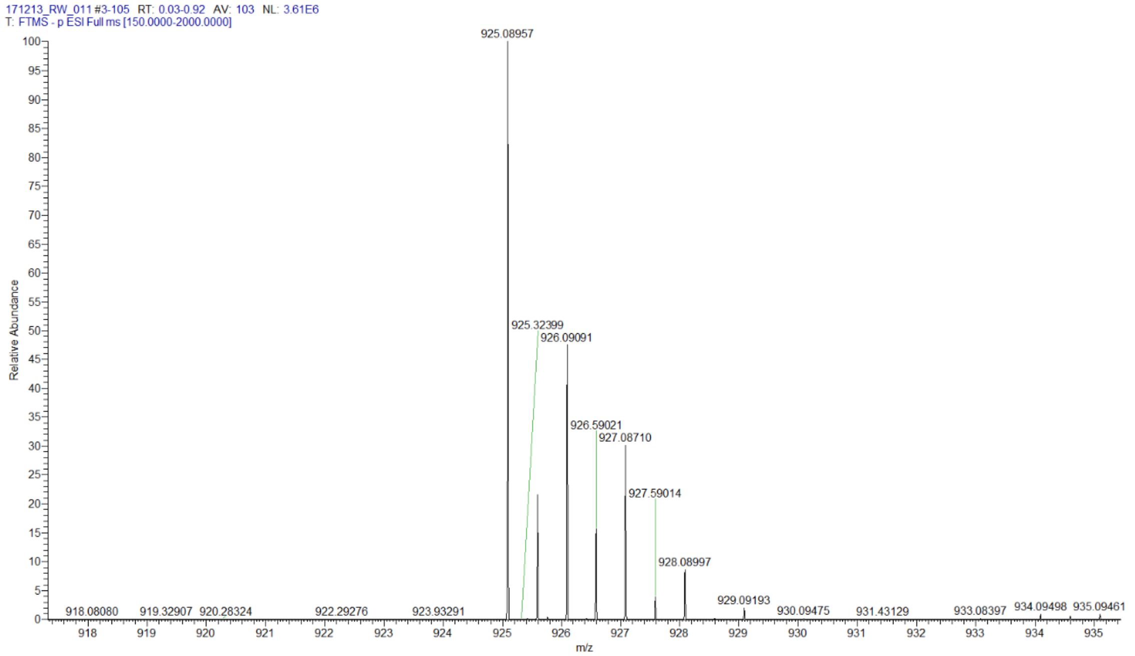

</td></tr>
</table>

^{1}H NMR (500.24 MHz)	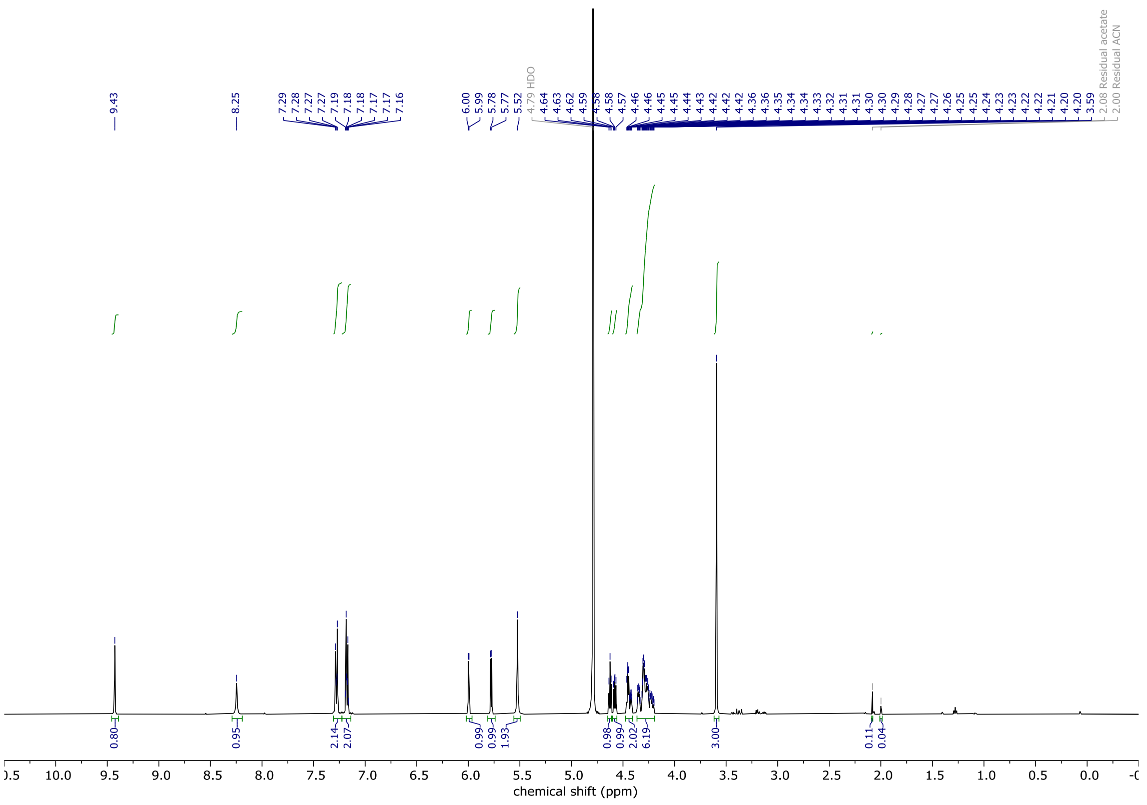
^{31}P NMR (202.49 MHz)	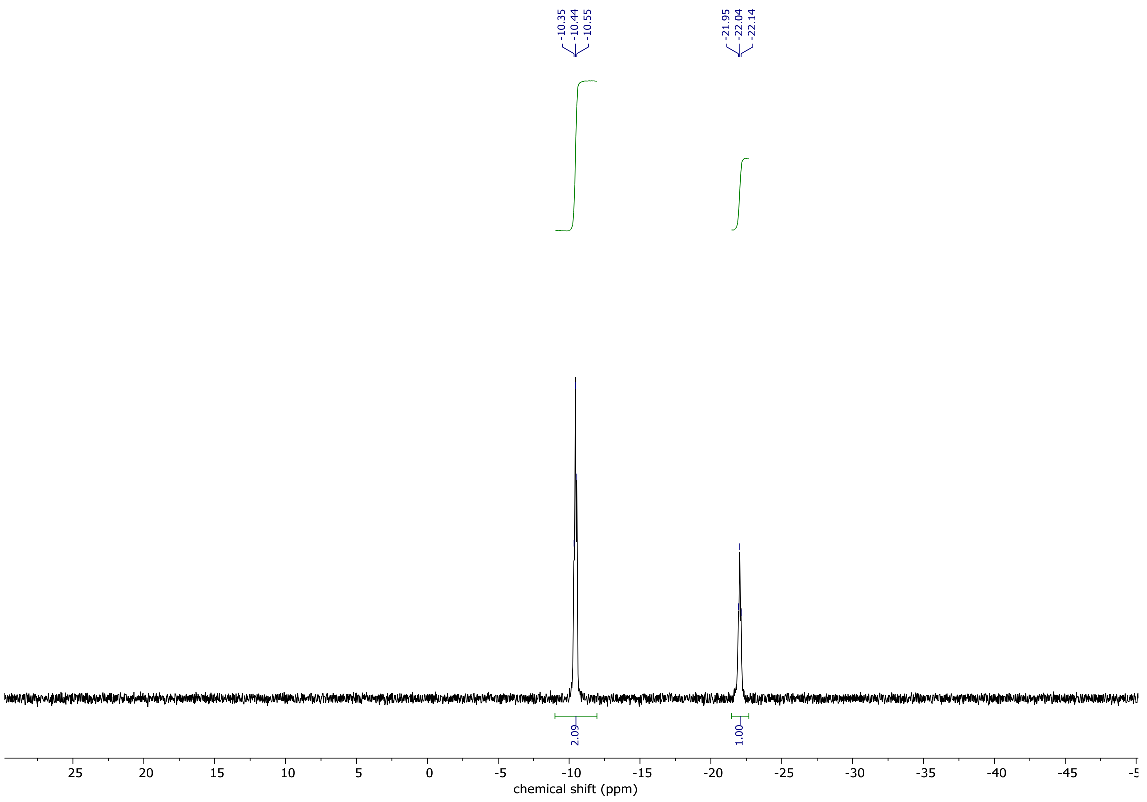

UV-VIS spectrum<br>(recorded in 0.1M phosphate buffer pH 7.0)	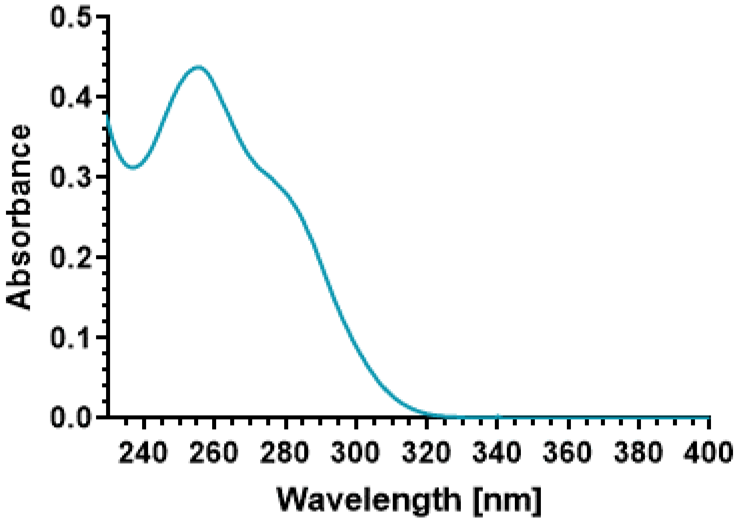
